# Insight into Genetic Characteristics of Identified SARS-CoV-2 Variants in Egypt from March 2020 to May 2021

**DOI:** 10.3390/pathogens11080834

**Published:** 2022-07-26

**Authors:** Wael H. Roshdy, Ahmed Kandeil, Rabeh El-Shesheny, Mohamed K. Khalifa, Ahmed A. Al-Karmalawy, Shymaa Showky, Amel Naguib, Nancy Elguindy, Manal Fahim, Hanaa Abu Elsood, Ahmed El Taweel, Azza Salamony, Amira Mohsen, Ghazi Kayali, Mohamed A. Ali, Amr Kandeel

**Affiliations:** 1 Central Public Health Laboratory, Ministry of Health and Population, Cairo 11613, Egypt; mohamed.kamal@omicsense.com (M.K.K.); shymaashowky@yahoo.com (S.S.); amelnaguib11@gmail.com (A.N.); nancy_el_guindy@hotmail.com (N.E.); azzasalamony@yahoo.com (A.S.); 2 Center of Scientific Excellence for Influenza Viruses, National Research Centre, Giza 12622, Egypt; ahmed.kandeil@human-link.org (A.K.); rabeh.elshesheny@human-link.org (R.E.-S.); ahmed.nageh@human-link.org (A.E.T.); 3 Department of Pharmaceutical Medicinal Chemistry, Faculty of Pharmacy, Horus University-Egypt, New Damietta 34518, Egypt; akarmalawy@horus.edu.eg; 4 Department of Surveillance and Epidemiology, Ministry of Health and Population, Cairo 11613, Egypt; fahimmanal@yahoo.com (M.F.); hanaaabuelsood@hotmail.com (H.A.E.); 5 Egypt Country Office, World Health Organization, Cairo 11613, Egypt; amahmed@who.int; 6 Human Link, Dubai 48800, United Arab Emirates; ghazi@human-link.org; 7 Preventive Sector, Ministry of Health and Population, Cairo 11613, Egypt; kandeelamr@yahoo.com

**Keywords:** SARS-CoV-2, Egypt, genetic, variants

## Abstract

Severe Acute Respiratory Syndrome Coronavirus 2 (SARS-CoV-2) was first detected in Egypt in February 2020. Data about the prevalence rates of the SARS-CoV-2 lineages are relatively scarce. To understand the genetic characteristics of SARS-CoV-2 in Egypt during several waves of the pandemic, we analyzed sequences of 1256 Egyptian SARS-CoV-2 full genomes from March 2020 to May 2021. From one wave to the next, dominant strains have been observed to be replaced by other dominant strains. We detected an emerging lineage of SARS-CoV-2 in Egypt that shares mutations with the variant of concern (VOC). The neutralizing capacity of sera collected from cases infected with C.36.3 against dominant strains detected in Egypt showed a higher cross reactivity of sera with C.36.3 compared to other strains. Using in silico tools, mutations in the spike of SARS-CoV-2 induced a difference in binding affinity to the viral receptor. The C.36 lineage is the most dominant SARS-CoV-2 lineage in Egypt, and the heterotrophic antigenicity of SARS-CoV-2 variants is asymmetric. These results highlight the value of genetic and antigenic analyses of circulating strains in regions where published sequences are limited.

## 1. Introduction

Severe Acute Respiratory Syndrome Coronavirus 2 (SARS-CoV-2) is an enveloped, single-stranded, positive-sense RNA virus from the *Coronaviridae* family. SARS-CoV-2 is prone to a multitude of recombinations and mutations in its genome that could lead to changes in viral protein structure, binding affinity, viral transmission, diagnosis, vaccine efficacy, and antiviral susceptibility [1,2,3]. The symptoms of infection with SARS-CoV-2 tend to resemble symptoms of other viral and bacterial infections that range in severity from mild respiratory and gastrointestinal illness to Acute Respiratory Distress Syndrome (ARDS).

Coronavirus disease (COVID-19) was introduced into Africa via Nigeria, Egypt, and South Africa during the first quartile of 2020 [4]. On 14 February 2020, the Egyptian Ministry of Health and Population (MOHP) announced the detection of the first human COVID-19 infection in Egypt in a Chinese tourist [5]. A few days later, the MOHP discovered another source of transmission on board of a Nile cruise ship traveling between Luxor and Aswan where COVID-19 infections were confirmed amongst 33 passengers and 12 staff members [5]. Since this first influx of cases, Egypt has ranked as one of the top five countries reporting COVID-19 cases in Africa [5]. National efforts have been exerted to control the virus spreading through a comprehensive control plan strategy set by the Egyptian government. The control strategy included applying various types of lockdowns, increasing public awareness about COVID-19 and the importance of using community protective equipment and disinfectants, screening for SARS-CoV-2 before entering the country, preparing healthcare facilities and reinforcing infection control practices among healthcare workers, ensuring vaccine availability, and establishment of COVID-19 treatment protocols. Unfortunately, these attempts and efforts to control the spread of virus have not succeeded. As of 4 March 2022, 489,000 Egyptian cases and 24,162 deaths have been reported to WHO from MOHP.

Next-generation sequencing provides high-quality, full-scale genome sequences and constitutes a rapid approach to determine genetic variations and new emerging variants of SARS-CoV-2. Analysis of genome sequences gives insight into the pattern of global distribution, genetic diversity during the pandemic, and the dynamics of newly emerging variants. Monitoring variants is essential for the control strategy plan to ensure the effectiveness of vaccination and treatment protocols. Several public databases such as The Global Initiative on Sharing All Influenza Data (GISAID) (www.gisaid.org accessed on 3 January 2022), the NCBI SARS-CoV-2 database (https://www.ncbi.nlm.nih.gov/sars-cov-2 accessed on 3 January 2022), and the Virus Pathogen Database and Analysis Resource (ViPR) (https://www.viprbrc.org accessed on 3 January 2022) share genomic information of circulating viruses and provide further insight into molecular variations among SARS-CoV-2 genomes. In the same context, several online classification and analysis tools have been developed such as GISAID, Nextstrain SARS-CoV-2, Pangolin (https://cov-lineages.org/resources/pangolin.html accessed on 3 January 2022), Nextclade (https://clades.nextstrain.org/ accessed on 3 January 2022), and others.

Several lineages and sublineages of SARS-CoV-2 have been identified with variable prevalence rates between the North and the South, depending on the source of introduction [4]. The continuous direct or indirect spreading of SARS-CoV-2 from person to person, particularly among those who are fully vaccinated, drives the virus to evolve into new Variants of Interest (VOIs) or Variants of Concern (VOCs), by escaping different hosts and immunological pressures [6]. A number of VOCs and VOIs have been documented worldwide, including the alpha variant (B.1.1.7 (Pango lineage) or GRY (GISAID clade), United Kingdom), beta variant (B.1.351 or GH/501Y.V2, South Africa), gamma variant (P.1 or GR/501Y.V3, Brazil), delta variant (B.1.617.2 or G/478K.V1, India), Lambda (C.37 or GR/452Q.V1, Peru), Mu (B.1.621 or GH, Colombia) [7,8,9], and Omicron (B.1.1.529 or GRA, South Africa). Several other variants of unknown risks have been detected globally and therefore designated as Variants Under Monitoring (VUMs) [2,4].

Although Egypt is the largest connecting point between Europe, Asia, and Africa with a remarkable number of intercontinental airlines directly linked to Europe, data about the prevalence rates of the SARS-CoV-2 lineages and sublineages including the VOIs, VOCs, and VUMs are relatively scarce. In this article, we report the distribution of SARS-CoV-2 variants, highlighting the prevalence of certain mutations and their cross reactivity, in addition to hACE2 binding affinity to the most detected strains in Egypt between March 2020 and May 2021. We present data on the genetic characteristics of the evolution of SARS-CoV-2 variants in the first year of pandemic in Egypt, highlighting the characteristics of spike protein mutations. To achieve this, we measured neutralizing antibody titers in sera collected from confirmed cases of SARS-CoV-2 and performed protein–protein docking experiments to illustrate the characteristics of virus variants circulating in Egypt.

## 2. Results

### 2.1. Epidemiology of SARS-CoV-2 in Egypt

**Figure 1:**
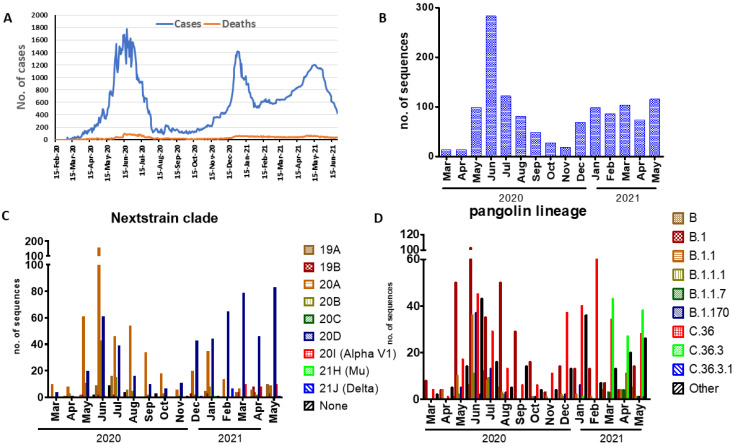
Prevalence rates of SARS-CoV-2 lineages and sublineages in Egyptian isolates from March to May 2021. (**A**) Waves of SARS-CoV-2 were recorded in Egypt based on the available epidemiological data. (**B**) Distribution of full-genome sequences were submitted to the database. (**C**,**D**) The prevalence ratio of the detected SARS-CoV-2 lineages using Nextclade and Pangolin analysis.

Three SARS-CoV-2 waves were recorded in Egypt based on the available epidemiological data (Figure 1A). The first wave began in April 2020 and receded by August 2020. It was associated with the religious and cultural celebrations of the holy month of Ramadan as well as the yearly wedding celebration season. The second wave coincided with the onset of winter weather. The third reported wave followed the mass gatherings associated with the start of the following year’s religious and cultural celebrations in 2021.

### 2.2. Clades and Lineage Distribution of SARS-CoV-2

The analysis included 1256 whole-genome sequences of SARS-CoV-2 strains submitted to the GISAID until 31 May 2021 from Egypt. The number of submitted sequences by month during the duration of our study (*n* = 15 months) is shown in Figure 1B. We noted that the number of submitted sequences was closely correlated with the number of cases shown in the epidemiological curve (Figure 1A). Full-genome sequences (*n* = 778) were submitted to the database in 2020 and 478 were submitted (*n* = 478) by May 2021. Seven clades according to GISAID classification for SARS-CoV-2 were identified in Egypt: G (*n* = 164), GH (*n* = 347), GR (*n* = 580), GRY (*n* = 3), L (*n* = 29), O (*n* = 105), and S (*n* = 28), of which GR was the predominant clade. Six clades were detected in 2020: G (*n* = 130), GH (*n* = 305), GR (*n* = 255), L (*n* = 16), O (*n* = 64), and S (*n* = 8), and in 2021, seven clades were detected. At the beginning of the pandemic in March 2020, the three clades G, GH, and O were identified. However, clade G was not detected in March and April, but in May 2021. The GRY clade (*n* = 3) was reported in April and May 2021.

According to Nextclade analysis, 529 of the identified genomes belonged to Nextstrain clade 20D, 484 belonged to clade 20A, 94 belonged to clade 20B, 59 belonged to clade 19A, 28 belonged to clade 19B, and 3 belonged to clade 20C. The remaining sequences were classified as variants, 29 belonged to clade 20I (Alpha, V1) which was identified in March—May 2021, nine sequences belonged to 21J (Delta), and one strain belonged to clade 21H(Mu) (Figure 1C).

Furthermore, analysis of sequences using Pangolin COVID-19 platform showed the presence of 63 lineages. Lineages B (*n* = 37), B.1 (*n* = 363), B.1.1 (*n* = 72), B.1.1.1 (*n* = 27), B.1.1.7 (*n* = 8), B.1.170 (*n* = 68), C.36 (*n* = 347), and C.36.3 (*n* = 109) were found to be the predominant lineages (Figure 1D). The lineage C.36 was identified in the beginning of the pandemic in March 2020 and continued to circulate till May 2021 and divided into two other lineages C.36.3 and C.36.3.1 in January 2021 and May 2021, respectively (Figure 1D).

### 2.3. Phylogenetic Reconstruction of SARS-CoV-2 in Egypt

**Figure 2:**
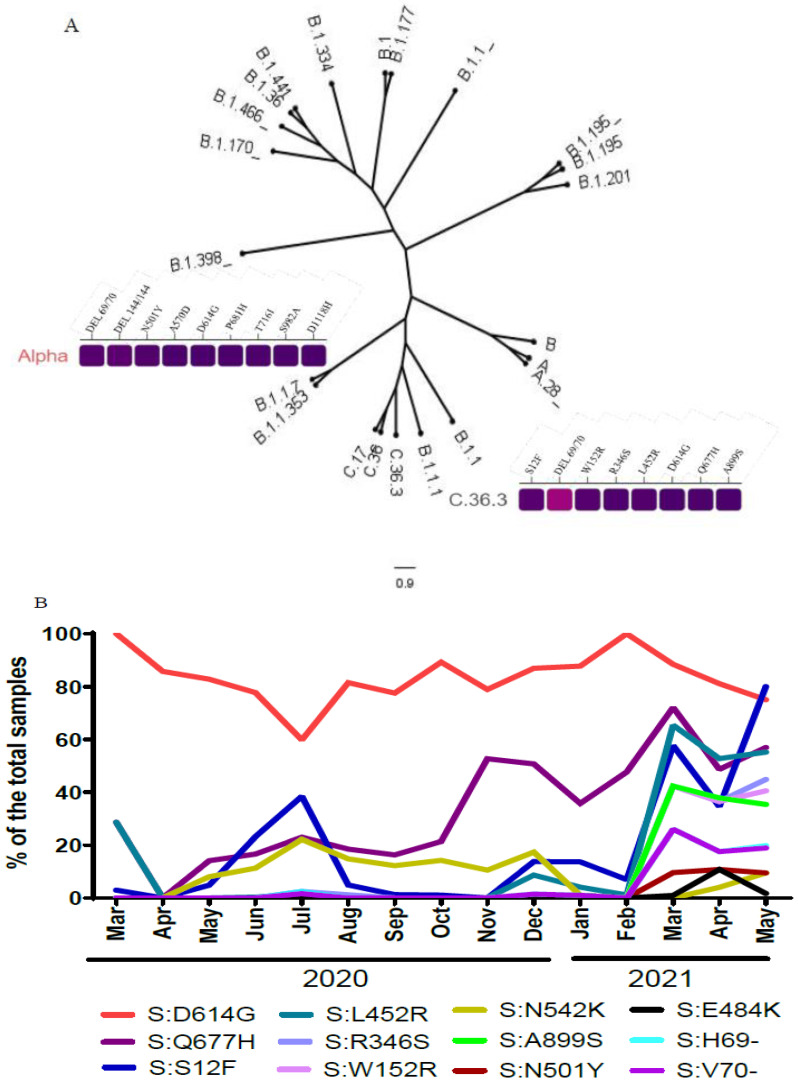
(**A**) Maximum likelihood (ML) phylogeny of Egyptian SARS-CoV-2 whole-genome sequences with heat map of spike amino acid mutations associated with the VOC (Alpha) and C.36.3 lineage. (**B**) Distribution of SARS-CoV-2 spike mutations in Egypt over time.

Phylogenetic analysis of the full genome sequences showed that Egyptian strains diversified into several lineages (Figure 2). Among VOCs, only B.1.1.7 was detected in limited number of sequences. The most dominant is the C.36 that is subsequently subdivided into C.36.1, C.36.2, and C.36.3 (Figure 2).

The variant C.36.3 was identified first in Egypt in March 2020 and continued to be prevalent until May 2021. Meanwhile, it acquired several mutations (Table 1) including the S12F, W152R, R346S, L452R, D614G, Q677H, A899S substitutions, and 69–70 Deletion (Del 69–70) in spike protein. R346S, L452R, and Q677H mutations were associated with better receptor binding affinities and consequently increased transmissibility and reduced ability to neutralize convalescent or vaccinated sera [10]. The D614G mutation was also associated with increased SARS-CoV-2 transmissibility [11]. Mutations detected in spike protein of C.36.3 (e.g., W152R and ΔH69-V70) are probably associated with lower sensitivity to monoclonal antibodies (mAbs) and vaccine sera [10,11,12].

### 2.4. Mutational Pattern Analysis

We performed a comprehensive mutational pattern analysis of the SARS-CoV-2 strains from this study in Egypt. In total, we identified mutations in S, N, M, ORF1a, ORF1b, ORF3a, ORf8, and ORF7b genes as compared with the reference strain Wuhan/WIV04/2019.

The D614G mutation is an amino acid substitution in the spike protein that was discovered early in the pandemic and has since become the most common SARS-CoV-2 mutation worldwide. In our study, 1016 samples had D614G and 79 samples from GISAID clade L, O, and S did not have this mutation (Figure 2B). The VOCs (Alpha, Beta, and Gamma) have a common mutation N501Y in spike gene, we show that N501Y mutation has been identified in VOCs Alpha (B.1.1.7) and two strains (B.1 and B.1.1.10 Lineages). The N501T was detected in A.28 (*n* = 20), B.1.1 (*n* = 4), and C.36 (*n* = 3). E484K spike mutation was detected in B.1.44.1 (*n* = 1) and C.38 (*n* = 11). Spike protein H69/V70 deletion was observed in 68 and 66 (5.4%, and 5.2%) sequences, respectively. Most deletions of H69/V70 were detected in C.36.3 Lineage (*n* = 49) (Table 2, Figure 2B).

The genetic diversity of SARS-CoV-2 has increased rapidly, we observed other mutations in other genes of SARS-CoV-2 (Supplementary Materials Table S1). Most mutations were detected in N, ORF1a, and ORF1b and few mutations in M and E genes, indicating that these proteins are highly conserved among these viruses.

### 2.5. Microneutralization Assay of Infected Cases with C.36.3 Lineage

**Figure 3:**
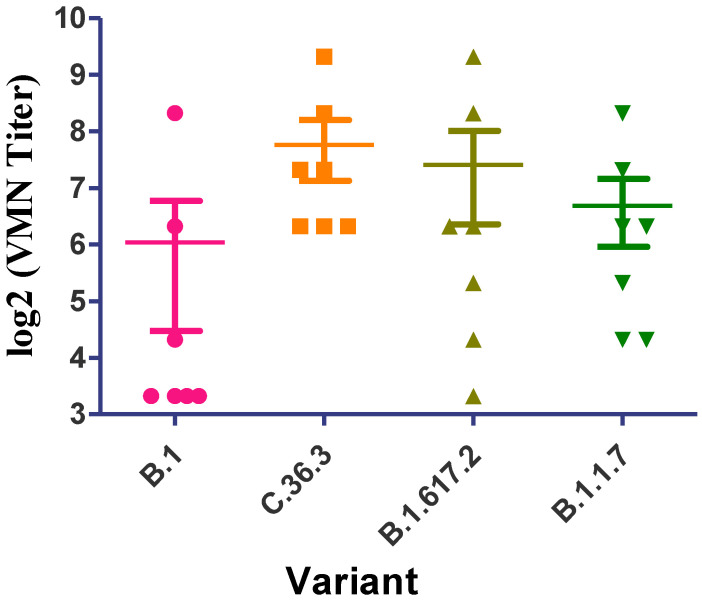
Neutralizing antibody responses against the B.1, C.36.3, B.1.617.2, and B.1.1.7 variants.

We collected sera samples from seven convalescent patients who were infected with the SARS-CoV-2 C.36 strain and exhibited mild or moderate symptoms from May–June 2021. Using an in vitro neutralization assay with authentic viral isolates of B.1, C.36.3, B.1.1.7, and B.1.617.2, we measured the neutralizing capacity of the collected sera from infected patients with C.36 (Figure 3). All sera had detectable neutralizing antibody titers against C.36.3 (7.7 ± 1.1 log2 VMN titer). The cross reactivity of sera against B.1, B.1.1.7, and B.1617.2 was 6, 7.4, and 6.6 log2 VMN titer, respectively (Figure 3). The high prevalence of circulating C.36 and C.36.3 variants and their ability to continuously acquire crucial new mutations to escape increased selective pressures in vaccinated populations emphasize the importance of their close monitoring and inclusion in the VUM/VOCs list.

### 2.6. Molecular Docking and Protein Simulation

#### 2.6.1. Protein–Ligand Interaction Fingerprints (PLIF) of B.1, C.36, and C.36.3 Lineage Strains with the Target hACE2

The protein–ligand interaction fingerprints (PLIF) were studied for each selected pose from the three applied docking processes. Regarding the hCoV-19/Egypt/NRC-03/2020 (B.1 lineage)-hACE2 docking process, it was noted that Glu231 and Val604 of the RBD of the hCoV-19/Egypt/NRC-03/2020 (B.1 lineage) S protein (12.4%) were responsible for the interactions. Moreover, Gln24, Asp136, Glu197, Glu224, and Asn601 of the RBD formed the major binding interactions (Supplementary Materials Figure S1A).

On the other hand, the PLIF of the hCoV-19/Egypt/NRC-369/2021 (C.36.3 lineage)-hACE2 docking process showed that both Asn601 and Val604 of the RBD of the hCoV-19/Egypt/NRC-369/2021 (C.36.3 lineage) S protein represented the most essential amino acids involved in the interactions (13.1%). Furthermore, Asp615, Lys465, Glu231, Asp216, and Glu197 amino acids were found to form many other binding interactions as well (Supplementary Materials Figure S1B).

Furthermore, the PLIF of the hCoV-19/Egypt/NRC-6071/2021 (C.36 lineage)-hACE2 docking process showed that Glu197 represented the most essential amino acid from the RBD of the hCoV-19/Egypt/NRC-6071/2021 (C.36 lineage) S protein responsible for the interactions (11.2%). Additionally, Tyr255, Lys596, Asn601, and Val604 amino acids formed other interactions with the hACE2 (Supplementary Materials Figure S1C).

#### 2.6.2. Visualization and Description of the Selected Docking Poses

**Figure 4:**
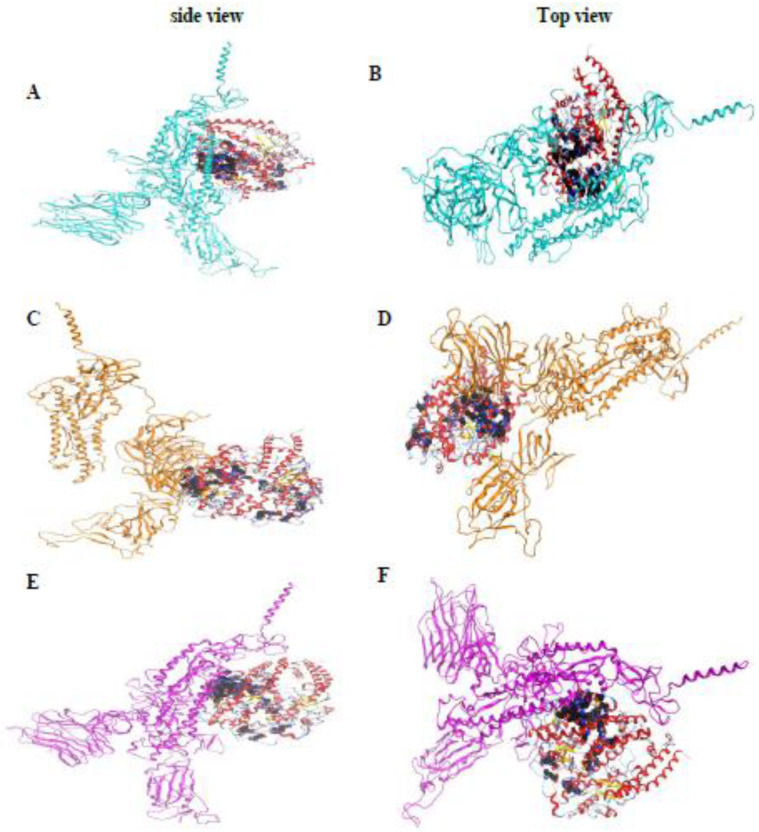
Protein–ligand interactions of the three SARS-CoV-2 Egyptian strains towards the hACE2 receptor. (**A**,**B**) The large area of binding interactions between the hCoV-19/Egypt/NRC-03/2020 S protein (represented in turquoise color) and the RBD region of the hACE2 receptor (represented in black color). (**C**,**D**) The large area of binding interactions between the hCoV-19/Egypt/NRC-369/2021 S protein (represented in orange color) and the RBD region of the hACE2 receptor (represented in black color). (**E**,**F**) The large area of binding interactions between the hCoV-19/Egypt/NRC-6071/2021 S protein (represented in purple color) and the RBD region of the hACE2 receptor (represented in black color).

The best pose in each docking process was selected depending on the score, rmsd_refine value, and the binding interactions for further studies. For the hCoV-19/Egypt/NRC-03/2020 (B.1 lineage)-hACE2 docking process, pose number 2 was selected with a binding score of −79.82 kcal/mol and an rmsd_refine value of 0.68. It was obvious that the hCoV-19/Egypt/NRC-03/2020 (B.1 lineage) S protein (represented in turquoise color) fitted with the RBD region of the hACE2 receptor (represented in black color), which explains largely the expected intrinsic binding affinity as represented in Figure 4A,B.

Furthermore, regarding the hCoV-19/Egypt/NRC-369/2021 (C.36.3 lineage)-hACE2 docking process, pose number 3 was selected with a binding score of -91.47 kcal/mol and an rmsd_refine value of 1.66. Moreover, the hCoV-19/Egypt/NRC-369/2021 (C.36.3 lineage) S protein (represented in orange color) bound the RBD region of the hACE2 receptor (represented in black color) which indicates the expected superior binding affinity, as depicted in Figure 4C,D.

However, for the hCoV-19/Egypt/NRC-6071/2021 (C.36 lineage)-hACE2 docking process, pose number 3 was selected with a binding score of -96.00 kcal/mol and an rmsd_refine value of 0.31. Notably, the hCoV-19/Egypt/NRC-6071/2021 (C.36 lineage) S protein (represented in purple color) achieved the best binding score with the RBD region of the hACE2 receptor (represented in black color) which explains its great binding affinity, as represented in Figure 4E,F.

## 3. Discussion

Since its detection in Egypt in March 2020, Severe Acute Respiratory Syndrome Coronavirus 2 (SARS-CoV-2) strains have varied. The number of COVID-19 cases in Egypt has risen steadily over the last two years, with the first recorded case registered on 14 February 2020, according to the World Health Organization (WHO) and Egyptian officials. The initial wave of the pandemic in Egypt was dominated by lineage B viruses (mainly B.1), while the second and third waves were dominated by C-like lineages (especially C.36).

Genomic epidemiology allows for the detection of new variants as well as a better understanding of how individual virus lineages’ mutations contribute to their relative adaptive advantages. This information can then be utilized to inform the development of vaccines and treatments, as well as establish effective pandemic control measures. In this study, we analyzed 1256 SARS-CoV-2 full genome sequences from Egypt. We have identified a novel SARS-CoV-2 cluster in Egypt, descended from the VUM in lineage C.36, L452R with an additional spike protein substitution at amino acid position 677, namely Q677H. The cluster of cases carries the core substitutions of the C.36 lineage, and was first found in the UK, USA, and United Arab Emirates in January 2021 and has risen in frequency to comprise the majority of sequenced cases in Egypt. They also contain mutation L452R from VOI substitutions which is present in B.1.526.1, B.1.427, and B.1.429 lineages, as well as B.1.617 lineage and sublineages. The novel variant strains also contain del69–70 with or without 142-143-144 del, which represents an important mutation in a VOC that has been previously observed in the Alpha variant, raising interest in potential convergent evolution. Independently, both C.36 lineages with L452R substitutions and 69–70 del substitutions have increased in prevalence. The impact of the detected VOC on viral transmission, vaccine effectiveness, and viral pathogenicity requires further investigation.

We examined 1256 SARS-CoV-2 sequences for mutation countryside tracking and observed that the most common subclade of SARS-CoV-2 in Egypt was D614G/Q57H/V5F/G823S, consistent with the findings of Lamptey et al. [13]. There was only one Egyptian sequence where four mutations occurred at the same time [13]. SARS-CoV-2 sequences examined in the current study are all identical and closely related to Wuhan-Hu-1. A previous study found >99.8 percent identity with the reference isolate (NC 045512) [14]. Based on the spike amino acid sequence, the Pangolin lineage C.36 can be separated into three sublineages. The C.36.3 sublineage, which was discovered in January 2021 and has a similar global distribution with highest prevalence in Egypt, is the focus of this research. With the common spike mutation L452R and deletion 69/70HV, C.36 sublineages reached the pinnacle of their outbreak in May 2021. Sublineage 3 was initially identified in late January 2021, and its prevalence has been slowly increasing since then, while the global incidence has remained modest. It has a distinct geographic distribution from the other C.36 sublineages, being found primarily in Europe and the United States, as well as a greater number of spike variants that are preserved among samples belonging to it.

The following mutations and deletions have been identified: S12F, H69-V70, W152R, R346S, L452R, D614G, Q677H, and A899S. It is worth noting that VOC B.1.427/B.1.429 (Epsilon) has analogous mutations (S13I and W152C) in the N-terminal domain (NTD) that significantly reduce the neutralizing potency of antibodies specific for this domain. The NTD-specific neutralization escape of H69-V70, which is found in VOC B.1.1.7 (Alpha) and VOI B.1.525, is also well described (Eta). The R346S and L452R mutations are found in the RBD of SARS-CoV-2 isolates of VOI B.1.427/B.1.429, VOI B.1.526.1 (Iota), and VOI B.1.617.2 (Delta) [15]. Resistance to the therapeutically approved monoclonal antibody (mAb) LY-CoV555 [16], as well as a significant fraction of other anti-RBD mAbs in the setting of VOC B.1.427/B.1.429 pseudoviruses have been reported [12,17,18]. Finally, the spike contains the D614G mutation, which promotes SARS-CoV-2 transmissibility, the Q677H mutation, which is found in VOI B.1.525, and the rare A899S mutation. Overall, C.36.3 has a spike structure similar to VOI B.1.427/B.1.429, which has been reported as an important amino acid of reactivity to mAbs [19], as well as other NTD and RBD mutations/deletions. Until the end of May 2021, approximately 21% of the Egyptian population had received at least one dose of vaccination, while only 6% of the total population had been fully vaccinated.

Based on the above, we can conclude that Glu231, Asn601, Val604, and Glu197 amino acids are the binding domains responsible for the binding interactions of the different studied strains of SARS-CoV-2, hCoV-19/Egypt/NRC-03/2020 (B.1 lineage), hCoV-19/Egypt/NRC-369/2021 (C.36.3 lineage), and hCoV-19/Egypt/NRC-6071/2021 (C.36 lineage) strains with the target hACE2 receptor.

We can also conclude that the hCoV-19/Egypt/NRC-6071/2021 (C.36 lineage) strain of SARS-CoV-2 shows superior binding affinity towards the RBD of the hACE2 with expected intrinsic activity as well. This was followed by the hCoV-19/Egypt/NRC-369/2021 (C.36.3 lineage) and then the hCoV-19/Egypt/NRC-03/2020 (B.1 lineage) strains in descending order. Finally, these observations indicate the severity level of the hCoV-19/Egypt/NRC-6071/2021 (C.36 lineage) strain of SARS-CoV-2 to infect humans, but at the same time, not very distant from the hCoV-19/Egypt/NRC-369/2021 (C.36.3 lineage) and hCoV-19/Egypt/NRC-03/2020 (B.1 lineage) strains severity as well. However, more advanced in vitro and in vivo tests are highly recommended to confirm the aforementioned docking results.

In this article, we have shed light on the viral dynamics underlying Egypt’s COVID-19 outbreak by combining genomic and epidemiological data. We have shown, for instance, that non-VOC lineages can evolve rapidly within a particular country to acquire adaptive mutations required to maintain community transmission and change the prognosis of SARS-CoV-2 infections from mild to severe if they are not properly managed.

## 4. Materials and Methods

### 4.1. Sample Collection

Oropharyngeal or nasopharyngeal swabs were obtained from visa applicants, travelers from Egypt, and patients in different locations all over Egypt between 1 March 2020 and 1 June 2021. Laboratory technicians collected all clinical specimens from the Central Public Health Laboratory, Ministry of Health and Population, Egypt and the Centre of Scientific Excellence for Influenza Viruses, National Research Centre, Egypt. This study was approved by the Research Ethics Committee of the National Research Centre (Egypt) (protocol number 14 155, dated 22 March 2020).

The collected samples were processed and subjected to nucleic acid extraction using the Chemagic 360 machine (PerkinElmer Inc., TURKU, Finland). A VIASURE SARS-CoV-2 Real-Time PCR Detection Kit was used to identify SARS-CoV-2 RNA (ORF1ab and N gene) (Certest Biotec SL, Zaragoza, Spain). Positive samples were verified for SARS-CoV-2 using a Cobas 6800 system, whereby the RT-PCR runs were performed in duplicate and according to the manufacturer′s instructions (Roche Holding AG). The cycle threshold (Ct) of SARS-CoV-2 quantitative real-time PCR (RT-qPCR) in the samples selected for sequencing was less than 30.

### 4.2. SARS-CoV-2 Whole Genome Sequencing

The extracted total RNA was subjected to ribosomal RNA removal using the Ribo-Zero rRNA Removal Kit, followed by 1st and 2nd strand cDNA synthesis using the truSeq whole RNA (Illumina, San Diego, CA, USA) according to the manufacturer’s instructions and sequenced on the Miseq sequencer (Illumina). Genomic analysis and strain typing of SARS-CoV-2 variants is one of the sequence analysis techniques available with the truSeq assay. Adequate sequencing requires depth coverage of at least 100 strain-distinguishing regions of the open reading frame 1a (ORF1a), S, N, and ORF8 genes. With the exception of two sequences, all samples produced sufficient strain-typeable sequences, and no samples had a mixed population of viruses. The reads were aligned with the reference genome (NC_045512.2) using CLC Genomics Workbench version 20 (CLC Bio, Qiagen, Aarhus, Denmark) through workflow, and whole genome sequences were submitted to the GISAID platform.

### 4.3. Sequence Data Lineage Classification

The complete Egyptian genomes and their metadata, available on the GISAID platform, with collection dates between 17 February 2020 and 28 February 2021, were downloaded. Pangolin (https://cov-lineages.org/ accessed on 3 January 2022) and NextStrain (https://nextstrain.org/blog/2021-01-06-updated-SARS-CoV-2-clade-naming (accessed on 3 January 2022)) criteria were used to classify the lineage and sublineage of the collected samples. For comparable viruses, novel mutation combinations were examined using the entire Egypt sequences database (March 2021–May 2021), and/or the GISAID database (https://www.gisaid.org/ accessed on 3 January 2022).

### 4.4. Phylogenetic Analysis

To infer the phylogeny of SARS-CoV-2 complete genomes, high-coverage sequences of Egyptian genomes were obtained from GISAID platform. Consensus sequences of whole genomes of SARS-CoV-2 strains and reference sequences were aligned using the MAFFT web server (https://mafft.cbrc.jp/alignment/server 3 January 2022). An inference maximum-likelihood tree was constructed using a sequence alignment file and IQ-TREE 1.6.1. For better visualization, the phylogenetic tree was modified using FigTree v1.4.2 software (http://tree.bio.ed.ac.uk/softw are/figtree 3 January 2022).

### 4.5. Microneutralization Assay

Vero E6 cells were seeded into 96-well plates 24 h before the experiment. Collected sera from vaccinated animals were used as positive controls. Human sera samples were decomplemented at 56 °C for 30 min. Serial 2-fold dilutions of sera starting with a dilution of 1:10 were mixed with equal volumes of 100 TCID50/mL of SARS-CoV-2 viruses. After 1 h of incubation at 37 °C, 35 μL of the virus–serum mixture was applied in duplicate to Vero-E6 cell monolayers in 96-well microtiter plates after washing cells with 1X PBS. After 1 h of adsorption, the inoculums were aspirated. The plates were then incubated for three more days at 37 °C in 5% CO_2_ in a humidified incubator. The highest serum dilution that completely protected the cells from Cytopathic Effect (CPE) was recorded as the neutralizing antibody titer.

### 4.6. Molecular Docking and Protein Simulation

#### 4.6.1. Protein–Protein Docking Studies

Three protein–protein docking studies were conducted to describe the possible interactions of three SARS-CoV-2 Egyptian strains, hCoV-19/Egypt/NRC-03/2020 (B.1 lineage), hCoV-19/Egypt/NRC-369/2021 (C.36.3 lineage), and hCoV-19/Egypt/NRC-6071/2021 (C.36 lineage), respectively, with the hACE2 receptor. The MOE 2019.0102 drug design suite [20] was used to carry out the docking studies to examine the binding affinities and modes for each of the three SARS-CoV-2 Egyptian strains with the hACE2 receptor extracted from the spike receptor-binding domain (PDB ID: 7DDN). We confirmed inhibitory potentials as well.

#### 4.6.2. Preparation of the Three Examined SARS-CoV-2 Egyptian Strains

Each of the three Egyptian SARS-CoV-2 strains were modeled using the protein builder of Molecular Operating Environment (MOE) 2019. The amino acids of the protein builder window were selected to draw the three SARS-CoV-2 Egyptian strains hCoV-19/Egypt/NRC-03/2020 (B.1 lineage), hCoV-19/Egypt/NRC-369/2021 (C.36.3 lineage), and hCoV-19/Egypt/NRC-6071/2021 (C.36 lineage) in three different processes.

#### 4.6.3. Preparation of the Target hACE2

The X-ray structure of the S protein of SARS-CoV-2 (at its open state) was extracted from the Protein Data Bank (PDB code: 7DDN) [21]. The open state of the S protein of SARS-CoV-2 provides the receptor-binding domain (RBD) in its opened orientation which allows the simulation of the possible epitope binding and interactions. Then, the hACE2 receptor was extracted from the SARS-CoV-2 spike receptor-binding domain. It was prepared for docking using the Quickprep protocol by applying the automatic correction step for all the predicted errors in the amino acids’ connections and types, followed by the addition of hydrogen atoms in the 3D geometry to their atoms. Additionally, the system atoms were selected to be free during the energy minimization as a final step for preparation.

#### 4.6.4. Protein–Protein Docking Processes

The protein–protein docking protocol was selected to evaluate the possible interactions between each of the three SARS-CoV-2 Egyptian strains, hCoV-19/Egypt/NRC-03/2020 (B.1 lineage), hCoV-19/Egypt/NRC-369/2021 (C.36.3 lineage), and hCoV-19/Egypt/NRC-6071/2021 (C.36 lineage)) against the hACE2 receptor in three successive different docking processes, respectively. The applied methodology is described as follows: the previously prepared hACE2 was selected in the three docking processes to be the receptor, then each prepared SARS-CoV-2 Egyptian strain (hCoV-19/Egypt/NRC-03/2020 (B.1 lineage), hCoV-19/Egypt/NRC-369/2021 (C.36.3 lineage), and hCoV-19/Egypt/NRC-6071/2021 (C.36 lineage), respectively) was inserted in the ligand place in each docking process. Each docking process was initiated separately using a hydrophobic patch potential as described earlier [22,23]. Additionally, the scoring tools were selected as default, 10,000 for the number of pre-placement poses, 1000 for the number of placement poses, and 100 for the number of refinement poses. Finally, after the end of each docking process, the obtained 100 poses were studied to select the most appropriate one having the best score, binding interactions, and rmsd_refine value to be studied further and described in detail.

## Figures and Tables

**Table 1:** Characteristic mutations of C.36.3 linage.

Gene	Amino Acid Mutations
ORF1a	E102K, A859V, T1246I, D1639N, P2287S, D2980N, D3222N, G3278S, S3687L, L3691S, T4090I, P314L, D1028Y
S	S12F, del69/70, W152R, R346S, L452R, D614G, Q677H, A899S
M	I82T
ORF7b	A43S
ORF8	S84L
N	R203K, G204R, G212V

**Table 2:** Spike mutations investigated and their proportion in our study.

Mutations	No	(%)	Nextclade	Pangolin	GISAID
S:Q677H	420	33.4	19A, 20A, 20B, 20D, 20I (Alpha, V1)	A, B, B.1, B.1.1, B.1.1.1, B.1.1.10, B.1.170, B.1.177, B.1.36, B.1.371, B.1.516, C.17, C.36, C.36.3, C.36.3.1	G, GH, GR, L, O, S
S:S12F	224	17.8	19A, 20A, 20B, 20D, 20I (Alpha, V1)	B.1, B.1.1, B.1.1.1, B.1.1.10, B.1.170, B.1.180, B.1.187, B.1.221, B.1.417, B.1.516, B.1.575, C.17, C.36, C.36.3, C.36.3.1	G, GH, GR, L, O
S:L452R	189	15.04	19A, 20A, 20D	B.1, B.1.1, B.1.1.1, B.1.1.10, B.1.187, B.1.516, C.36, C.36.3	G, GH, GR, O
S:R346S	128	10.19	19A, 20B, 20D	B.1.1, B.1.1.1, B.1.1.10, B.1.187, B.1.516, C.17, C.36.3, C.36.3.1	G, GH, GR, L, O
S:W152R	119	9.47	20D	B.1, B.1.1, B.1.1.10, B.1.187, B.1.516, C.36.3,	G, GR, O
S:N542K	118	9.39	20A, 20B, 20D	B.1, B.1.1, B.1.1.1, B.1.1.117, B.1.1.184, B.1.1.192, B.1.1.51, B.1.170, B.1.180, B.1.187, B.1.293, B.1.36.31, B.1.36.39, B.1.371, B.1.417, B.1.456, B.1.517, B.1.533, B.1.575, C.36, C.36.3, C.36.3.1, C.38	G, GH, GR, O
S:A899S	114	9.07	19A, 20D, 20I (Alpha, V1)	B.1.1, B.1.1.10, B.1.516, C.36.3	G, GR, O
S:N501Y	29	2.3	19A, 20A, 20I (Alpha, V1)	B.1, B.1.1.10, B.1.1.7	G, GR, GRY
S:E484K	12	0.95	20A, 20D	B.1.441, C.38	G, GR, O
S:H69-	68	5.41	19B, 20A, 20D, 20I (Alpha, V1)	A.28, B.1, B.1.1, B.1.1.1, B.1.1.7, B.1.187, B.1.516, C.17, C.36.3,	G, GH, GR, GRY, S
S:V70-	66	5.25	19B, 20D, 20I (Alpha, V1)	A.28, B.1, B.1.1, B.1.1.1, B.1.1.7, B.1.187, B.1.516, C.17, C.36.3,	G, GH, GR, GRY, S

## Data Availability

All data used in this study are available on request from the corresponding author.

## Supplementary Materials

The following supporting information can be downloaded at: https://www.mdpi.com/article/10.3390/pathogens11080834/s1, Figure S1: Maximum likelihood (ML) phylogeny of Egyptian SARS-CoV-2 whole-genome sequences. Figure S2: cumulative of C.36.3 prevalence worldwide. Figure S3: PLIF for the Egyptian strains-hACE2 docking (in population form). Table S1: sequences from GISAID used in this analysis. Table S2: shows the most common amino acid mutations among different lineages occurring in Egypt between March 2020 and May 2021.
