# Supplementary material for: Insight into Genetic Characteristics of Identified SARS-CoV-2 Variants in Egypt from March 2020 to May 2021
Source: Pathogens. 2022 Jul 26;11(8):834. doi: 10.3390/pathogens11080834 (PMC9330621; doi:10.3390/pathogens11080834)
Supplement: Supplementary file 1 [file pathogens-11-00834-s001.zip › Supplement Table S1.pdf]

Table S1: sequences from GISAID used in this analysis.

strain	qc.overallScore	qc.overallStatus	gisaid_epi_isl	length	Nextstrain_clade	pangolin_lineage	GISAID_clade
hCoV-19/Egypt/CCHE57357-A-54/2020	0	good	EPI_ISL_812827	29903	19A	B	O
hCoV-19/Egypt/ARMY-47/2021	0	good	EPI_ISL_1936223	29898	19A	B	L
hCoV-19/Egypt/ARMY-282/2021	0	good	EPI_ISL_1936133	29886	19A	B	L
hCoV-19/Egypt/ARMY-48/2021	0	good	EPI_ISL_1936224	29891	19A	B	L
hCoV-19/Egypt/MASRI-C4-022/2020	56.42361	mediocre	EPI_ISL_1165082	29796	20A	B.1	GH
hCoV-19/Egypt/CCHE57357_Wave_3_A022/2021	0	good	EPI_ISL_2566482	29903	19A	B	O
hCoV-19/Egypt/CCHE57357-P-25/2020	0	good	EPI_ISL_812859	29903	19A	B	L
hCoV-19/Egypt/ARMY-352/2021	0	good	EPI_ISL_1936282	29884	19A	B	O
hCoV-19/Egypt/ARMY-404/2021	0	good	EPI_ISL_1969081	29783	20I (Alpha, V1)	B.1.1.7	GR
hCoV-19/Egypt/EGY-S033/2020	1.5625	good	EPI_ISL_526988	29845	20A	B.1	GH
hCoV-19/Egypt/CCHE57357-A-55/2020	0	good	EPI_ISL_812828	29903	19A	B	O
hCoV-19/Egypt/ARMY-280/2021	0	good	EPI_ISL_1936131	29898	19A	B	L
hCoV-19/Egypt/CUNCI-HGC5I025/2020	529	bad	EPI_ISL_479716	29861	19A	A	O
hCoV-19/Egypt/MASRI-004/2020			EPI_ISL_1097025	24076	None	None	O
hCoV-19/Egypt/CPHL-S5/2021	723.4108	bad	EPI_ISL_3274152	29853	20D	C.36.3	GR
hCoV-19/Egypt/ARMY-49/2021	0	good	EPI_ISL_1936225	29886	19A	B	L
hCoV-19/Egypt/NRC-6128/2020	1369.749	bad	EPI_ISL_8189343	29873	19A	C.36	GR
hCoV-19/Egypt/NRC-6354/2020	77.25694	mediocre	EPI_ISL_8189308	29871	20A	B.1	GH
hCoV-19/Egypt/NRC-6363/2020	11.11111	good	EPI_ISL_8189307	29903	20A	B.1	GH
hCoV-19/Egypt/NRC-5548OP/2020	32.08114	mediocre	EPI_ISL_8189341	29873	20B	B.1.1.1	G
hCoV-19/Egypt/NRC-5552NS/2020	740.2778	bad	EPI_ISL_8189339	29879	20D	B.1.1.1	G
hCoV-19/Egypt/NRC-5556OP/2020	0	good	EPI_ISL_8189346	29885	20D	C.36	GR
hCoV-19/Egypt/NRC-5530/2020	0	good	EPI_ISL_8189543	29871	20A	B.1.170	GH
hCoV-19/Egypt/NRC-7302/2020	12459.76	bad	EPI_ISL_8189345	29566	20D	B.1.1.51	GR
hCoV-19/Egypt/NRC-5574OP/2020	0	good	EPI_ISL_8189342	29903	20D	C.36	GR
hCoV-19/Egypt/NRC-7332/2020	1027.778	bad	EPI_ISL_8189344	29903	20A	B.1	GH
hCoV-19/Egypt/NRC-5551NS/2020	13948.13	bad	EPI_ISL_8189340	29867	20A	B.1.371	GH
hCoV-19/Egypt/CUNCI-HGC4I023/2020	0	good	EPI_ISL_479729	29796	20B	B.1.1	GR
hCoV-19/Egypt/CCHE57357_Wave_3_A_005/2021	177.7778	bad	EPI_ISL_2510688	29894	20I (Alpha, V1)	None	GR
hCoV-19/Egypt/C-VSVRI-SERVAC/2020	0	good	EPI_ISL_528386	29793	20A	B.1	GH
hCoV-19/Egypt/CPHL-NRC-20/2020	2.595679	good	EPI_ISL_794593	29741	20A	B.1	GH
hCoV-19/Egypt/CPHL-NRC-15/2020	11.40938	good	EPI_ISL_794597	29815	20A	B.1	G
hCoV-19/Egypt/CCHE57357-A-01/2020	0	good	EPI_ISL_812783	29903	20D	C.36	GR
hCoV-19/Egypt/CCHE57357-A-02/2020	2.777778	good	EPI_ISL_812784	29903	20A	B.1	G
hCoV-19/Egypt/CCHE57357-A-05/2020	0	good	EPI_ISL_812786	29903	20D	B.1.1.1	GR
hCoV-19/Egypt/CCHE57357-A-07/2020	2.777778	good	EPI_ISL_812788	29903	19A	B.1	O
hCoV-19/Egypt/CCHE57357-A-08/2020	0	good	EPI_ISL_812789	29903	20A	B.1	GH
hCoV-19/Egypt/CCHE57357-A-10/2020	0	good	EPI_ISL_812791	29903	20A	B.1	G
hCoV-19/Egypt/CCHE57357-A-13/2020	6.25	good	EPI_ISL_812793	29903	19A	B.1.1	O

hCoV-19/Egypt/CCHE57357-A-15/2020	1.5625	good	EPI_ISL_812795	29903	20D	C.36	GR
hCoV-19/Egypt/CCHE57357-A-18/2020	0	good	EPI_ISL_812796	29903	20D	C.36	GR
hCoV-19/Egypt/CCHE57357-A-27/2020	21.00694	good	EPI_ISL_812803	29903	19A	B.1.1	O
hCoV-19/Egypt/CCHE57357-A-45/2020	11.11111	good	EPI_ISL_812818	29903	20B	B.1.1	GR
hCoV-19/Egypt/CCHE57357-A-63/2020	0	good	EPI_ISL_812835	29903	20D	C.36	GR
hCoV-19/Egypt/CCHE57357-A-90/2020	2.777778	good	EPI_ISL_812847	29903	20D	C.36	GR
hCoV-19/Egypt/CCHE57357-A-93/2020	2.777778	good	EPI_ISL_812849	29903	20A	B.1	GH
hCoV-19/Egypt/CCHE57357-P-10/2020	6.25	good	EPI_ISL_812852	29903	20A	B.1	G
hCoV-19/Egypt/CCHE57357-P-17/2020	4.340278	good	EPI_ISL_812855	29903	20A	B.1	O
hCoV-19/Egypt/CCHE57357-P-20/2020	0	good	EPI_ISL_812856	29903	20A	B.1	GH
hCoV-19/Egypt/CCHE57357-P-22/2020	44.44444	mediocre	EPI_ISL_812857	29903	20A	B.1	GH
hCoV-19/Egypt/CCHE57357-P-30/2020	84.02778	mediocre	EPI_ISL_812862	29903	20A	B.1	GH
hCoV-19/Egypt/CCHE57357-P-38/2020	0.173611	good	EPI_ISL_812868	29903	20B	B.1.1	GR
hCoV-19/Egypt/CCHE57357-P-43/2020	0	good	EPI_ISL_812871	29903	19A	B	O
hCoV-19/Egypt/CUNCI-HGC8I040/2020	6.25	good	EPI_ISL_857322	29793	20D	C.36	GR
hCoV-19/Egypt/CUNCI-HGC8I039/2020	900	bad	EPI_ISL_857323	29793	20D	C.36	GR
hCoV-19/Egypt/CUNCI-HGC9I013/2020	11.11111	good	EPI_ISL_857328	29793	20D	C.36	GR
hCoV-19/Egypt/CUNCI-HGC9I028/2020	5.340278	good	EPI_ISL_857334	29793	20D	C.36	GR
hCoV-19/Egypt/CUNCI-HGC9I031/2020	50.17361	mediocre	EPI_ISL_857338	29793	20D	C.36	GR
hCoV-19/Egypt/CUNCI-HGC9I021/2020	101.5625	bad	EPI_ISL_857339	29793	20D	C.36	GR
hCoV-19/Egypt/CUNCI-HGC9I024/2020	18.36111	good	EPI_ISL_857341	29783	20A	B.1.466.1	GH
hCoV-19/Egypt/CUNCI-HGC9I032/2020	5.5625	good	EPI_ISL_857344	29792	20D	C.36	GR
hCoV-19/Egypt/CCHE57357-P-37/2020	0	good	EPI_ISL_812867	29903	19A	B	L
hCoV-19/Egypt/ARMY-353/2021	0	good	EPI_ISL_1936283	29891	19A	B	L
hCoV-19/Egypt/ARMY-MCL012/2020	39.0625	mediocre	EPI_ISL_907085	29884	20A	B.1	GH
hCoV-19/Egypt/ARMY-351/2021	0	good	EPI_ISL_1936281	29887	19A	B	L
hCoV-19/Egypt/ARMY-228/2021	21.00694	good	EPI_ISL_1936107	29877	20A	B.1	GH
hCoV-19/Egypt/ARMY-281/2021	0	good	EPI_ISL_1936132	29891	19A	B	L
hCoV-19/Egypt/NRC1/2020	0	good	EPI_ISL_1315064	29864	20A	B.1	GH
hCoV-19/Egypt/NRC2/2020	0	good	EPI_ISL_1315065	29864	20A	B.1	GH
hCoV-19/Egypt/CCHE57357_Wave_3_A001/2021	11.11111	good	EPI_ISL_2566467	29903	20D	C.36.3	GR
hCoV-19/Egypt/CCHE57357_Wave_3_A002/2021	14.0625	good	EPI_ISL_2566468	29903	20D	C.36.3	GR
hCoV-19/Egypt/CCHE57357_Wave_3_A003/2021	21.00694	good	EPI_ISL_2566469	29903	20D	C.36.3	GR
hCoV-19/Egypt/CCHE57357_Wave_3_A006/2021	62.67361	mediocre	EPI_ISL_2566471	29903	20D	C.36.3	GR
hCoV-19/Egypt/CCHE57357_Wave_3_A007/2021	177.7778	bad	EPI_ISL_2566472	29903	20D	C.36	O
hCoV-19/Egypt/CCHE57357_Wave_3_A012/2021	29.34028	good	EPI_ISL_2566473	29903	20D	C.36.3	GR
hCoV-19/Egypt/CCHE57357_Wave_3_A013/2021	34.02778	mediocre	EPI_ISL_2566474	29903	20I (Alpha, V1)	None	GR
hCoV-19/Egypt/CCHE57357_Wave_3_A015/2021	39.0625	mediocre	EPI_ISL_2566475	29903	20D	C.36.3	GR
hCoV-19/Egypt/CCHE57357_Wave_3_A016/2021	62.67361	mediocre	EPI_ISL_2566476	29903	20D	C.36.3	O
hCoV-19/Egypt/CCHE57357_Wave_3_A017/2021	146.0069	bad	EPI_ISL_2566477	29903	20D	C.38	GR
hCoV-19/Egypt/CCHE57357_Wave_3_A018/2021	56.25	mediocre	EPI_ISL_2566478	29903	20D	None	O

hCoV-19/Egypt/CCHE57357_Wave_3_A019/2021	8.506944	good	EPI_ISL_2566479	29903	20I (Alpha, V1)	None	GR
hCoV-19/Egypt/CCHE57357_Wave_3_A020/2021	17.36111	good	EPI_ISL_2566480	29903	20D	C.36.3	GR
hCoV-19/Egypt/CCHE57357_Wave_3_A021/2021	14.0625	good	EPI_ISL_2566481	29903	20D	B.1.1.10	GR
hCoV-19/Egypt/CCHE57357_Wave_3_A024/2021	21.00694	good	EPI_ISL_2566483	29903	20I (Alpha, V1)	None	GR
hCoV-19/Egypt/CCHE57357_Wave_3_A025/2021	189.0625	bad	EPI_ISL_2566484	29903	20D	C.36.3	GR
hCoV-19/Egypt/CCHE57357_Wave_3_A028/2021	0.173611	good	EPI_ISL_2566485	29903	20D	C.36.3	GR
hCoV-19/Egypt/CCHE57357_Wave_3_A029/2021	11.11111	good	EPI_ISL_2566486	29903	20D	C.36.3	GR
hCoV-19/Egypt/CCHE57357_Wave_3_A030/2021	0	good	EPI_ISL_2566487	29903	20D	C.36.3	GR
hCoV-19/Egypt/CCHE57357_Wave_3_A032/2021	14.0625	good	EPI_ISL_2566488	29903	20D	C.36.3	GR
hCoV-19/Egypt/CCHE57357_Wave_3_A033/2021	11.11111	good	EPI_ISL_2566489	29903	20D	C.36.3	GR
hCoV-19/Egypt/CCHE57357_Wave_3_A036/2021	4.340278	good	EPI_ISL_2566490	29903	20D	C.36.3	GR
hCoV-19/Egypt/CCHE57357_Wave_3_A038/2021	84.02778	mediocre	EPI_ISL_2566491	29903	20D	C.36.3	O
hCoV-19/Egypt/CCHE57357_Wave_3_A041/2021	434.0278	bad	EPI_ISL_2566493	29903	20D	C.36	GR
hCoV-19/Egypt/CCHE57357_Wave_3_A043/2021	6.25	good	EPI_ISL_2566494	29903	20D	C.36.3	GR
hCoV-19/Egypt/CCHE57357_Wave_3_A044/2021	0	good	EPI_ISL_2566495	29903	20D	C.36.3	GR
hCoV-19/Egypt/CCHE57357_Wave_3_A045/2021	44.44444	mediocre	EPI_ISL_2566496	29903	20D	C.36.3	O
hCoV-19/Egypt/CCHE57357_Wave_3_A047/2021	69.44444	mediocre	EPI_ISL_2566497	29903	20D	C.36.3	GR
hCoV-19/Egypt/CCHE57357_Wave_3_A049/2021	44.44444	mediocre	EPI_ISL_2566498	29903	20D	C.36.3	GR
hCoV-19/Egypt/CCHE57357_Wave_3_A050/2021	25	good	EPI_ISL_2566499	29903	20D	C.36.3	GR
hCoV-19/Egypt/CCHE57357_Wave_3_A053/2021	44.44444	mediocre	EPI_ISL_2566501	29903	20D	C.36	GR
hCoV-19/Egypt/CCHE57357_Wave_3_A054/2021	34.02778	mediocre	EPI_ISL_2566502	29903	20D	C.36	GR
hCoV-19/Egypt/CCHE57357_Wave_3_A057/2021	21.00694	good	EPI_ISL_2566503	29903	20I (Alpha, V1)	None	GR
hCoV-19/Egypt/CCHE57357_Wave_3_A058/2021	39.0625	mediocre	EPI_ISL_2566504	29903	20D	C.36	GR
hCoV-19/Egypt/CCHE57357_Wave_3_A059/2021	0	good	EPI_ISL_2566505	29903	20I (Alpha, V1)	B.1.1.7	GR
hCoV-19/Egypt/CCHE57357_Wave_3_A063/2021	76.5625	mediocre	EPI_ISL_2566506	29903	20D	C.36.3	GR
hCoV-19/Egypt/CCHE57357_Wave_3_A064/2021	166.8403	bad	EPI_ISL_2566507	29903	20D	C.36.3.1	GR
hCoV-19/Egypt/CCHE57357_Wave_3_A066/2021	56.25	mediocre	EPI_ISL_2566508	29903	20D	C.36.3	GR
hCoV-19/Egypt/CCHE57357_Wave_3_A067/2021	6.25	good	EPI_ISL_2566509	29903	20D	C.36.3	GR
hCoV-19/Egypt/CCHE57357_Wave_3_A068/2021	44.44444	mediocre	EPI_ISL_2566510	29903	20D	C.36.3	GR
hCoV-19/Egypt/CCHE57357_Wave_3_A069/2021	689.0625	bad	EPI_ISL_2566511	29903	20D	C.36	GR
hCoV-19/Egypt/CCHE57357_Wave_3_A070/2021	14.0625	good	EPI_ISL_2566512	29903	20I (Alpha, V1)	None	GR
hCoV-19/Egypt/CCHE57357_Wave_3_A073/2021	25	good	EPI_ISL_2566513	29903	20D	C.36.3	GR
hCoV-19/Egypt/CCHE57357_Wave_3_A074/2021	17.36111	good	EPI_ISL_2566514	29903	20D	C.36.3	GR
hCoV-19/Egypt/CCHE57357_Wave_3_A008/2021	126.5625	bad	EPI_ISL_2566515	29903	20D	C.38	O
hCoV-19/Egypt/CCHE57357_Wave_3_A040/2021	14.0625	good	EPI_ISL_2566516	29903	20I (Alpha, V1)	None	GR
hCoV-19/Egypt/CCHE57357_Wave_3_A055/2021	62.67361	mediocre	EPI_ISL_2566517	29903	20D	C.36.3	GR
hCoV-19/Egypt/CCHE57357_Wave_3_A_009/2021	177.7778	bad	EPI_ISL_2566518	29903	19A	B	L

hCoV-19/Egypt/CCHE57357_Wave_3_A_011/2021	434.0278	bad	EPI_ISL_2566519	29903	19A	B.1	L
hCoV-19/Egypt/CCHE57357_Wave_3_A_023/2021	177.7778	bad	EPI_ISL_2566520	29903	19A	B.23	L
hCoV-19/Egypt/CCHE57357_Wave_3_A_026/2021	400	bad	EPI_ISL_2566521	29903	20D	C.36.3	O
hCoV-19/Egypt/CCHE57357_Wave_3_A_027/2021	487.6736	bad	EPI_ISL_2566522	29903	19A	B.1	O
hCoV-19/Egypt/CCHE57357_Wave_3_A_035/2021	250.6944	bad	EPI_ISL_2566523	29903	20D	None	GR
hCoV-19/Egypt/CCHE57357_Wave_3_A_037/2021	434.0278	bad	EPI_ISL_2566524	29903	20D	None	O
hCoV-19/Egypt/CCHE57357_Wave_3_A_048/2021	177.7778	bad	EPI_ISL_2566525	29903	20D	C.36.3	GR
hCoV-19/Egypt/CCHE57357_Wave_3_A_052/2021	469.4444	bad	EPI_ISL_2566526	29903	20I (Alpha, V1)	B.1.1.10	GR
hCoV-19/Egypt/CCHE57357_Wave_3_A_056/2021	200.6944	bad	EPI_ISL_2566527	29903	20D	C.36.3	GR
hCoV-19/Egypt/CCHE57357_Wave_3_A_060/2021	136.1111	bad	EPI_ISL_2566528	29903	19A	B	L
hCoV-19/Egypt/CCHE57357_Wave_3_A_072/2021	400	bad	EPI_ISL_2566529	29903	20D	C.36.3	GR
hCoV-19/Egypt/CCHE57357_Wave_3_A_076/2021	136.1111	bad	EPI_ISL_2566530	29903	20D	C.36.3	GR
hCoV-19/Egypt/ARMY-350/2021	0	good	EPI_ISL_1936280	29886	19A	B	L
hCoV-19/Egypt/NRC-03/2020	0	good	EPI_ISL_430819	29709	20A	B.1	GH
hCoV-19/Egypt/MASRI-C5-040/2020	60.59028	mediocre	EPI_ISL_1586895	29860	20D	C.36	GR
hCoV-19/Egypt/ARMY-208/2021	39.0625	mediocre	EPI_ISL_1936102	29884	20D	C.36	GR
hCoV-19/Egypt/ARMY-210/2021	69.44444	mediocre	EPI_ISL_1936103	29878	20D	C.36	GR
hCoV-19/Egypt/ARMY-213/2021	146.0069	bad	EPI_ISL_1936104	29878	20D	C.38	GR
hCoV-19/Egypt/ARMY-219/2021	21.00694	good	EPI_ISL_1936105	29880	20A	B.1	GH
hCoV-19/Egypt/ARMY-241/2021	21.00694	good	EPI_ISL_1936106	29880	20A	B.1	GH
hCoV-19/Egypt/ARMY-239/2021	21.00694	good	EPI_ISL_1936108	29880	20I (Alpha, V1)	None	GR
hCoV-19/Egypt/ARMY-206/2021	21.00694	good	EPI_ISL_1936109	29884	20I (Alpha, V1)	None	GR
hCoV-19/Egypt/ARMY-249/2021	21.00694	good	EPI_ISL_1936110	29880	20I (Alpha, V1)	None	GR
hCoV-19/Egypt/ARMY-259/2021	21.00694	good	EPI_ISL_1936111	29879	20I (Alpha, V1)	None	GR
hCoV-19/Egypt/ARMY-250/2021	84.02778	mediocre	EPI_ISL_1936112	29884	20D	C.36	GR
hCoV-19/Egypt/ARMY-251/2021	84.02778	mediocre	EPI_ISL_1936113	29879	20D	C.36	GR
hCoV-19/Egypt/ARMY-290/2021	84.02778	mediocre	EPI_ISL_1936114	29884	20D	C.36	GR
hCoV-19/Egypt/ARMY-291/2021	84.02778	mediocre	EPI_ISL_1936115	29878	20D	C.36	GR
hCoV-19/Egypt/ARMY-252/2021	84.02778	mediocre	EPI_ISL_1936116	29884	20D	C.36	GR
hCoV-19/Egypt/ARMY-255/2021	84.02778	mediocre	EPI_ISL_1936117	29881	20D	C.36	GR
hCoV-19/Egypt/ARMY-256/2021	84.02778	mediocre	EPI_ISL_1936118	29879	20D	C.36	O
hCoV-19/Egypt/ARMY-257/2021	84.02778	mediocre	EPI_ISL_1936119	29881	20D	C.36	GR
hCoV-19/Egypt/ARMY-258/2021	84.02778	mediocre	EPI_ISL_1936120	29877	20D	C.36	GR
hCoV-19/Egypt/ARMY-260/2021	84.02778	mediocre	EPI_ISL_1936121	29884	20D	C.36	GR
hCoV-19/Egypt/ARMY-261/2021	84.02778	mediocre	EPI_ISL_1936122	29876	20D	C.36	O
hCoV-19/Egypt/ARMY-262/2021	84.02778	mediocre	EPI_ISL_1936123	29879	20D	C.36	GR

hCoV-19/Egypt/ARMY-264/2021	84.02778	mediocre	EPI_ISL_1936124	29883	20D	C.36	GR
hCoV-19/Egypt/ARMY-265/2021	84.02778	mediocre	EPI_ISL_1936125	29884	20D	C.36	O
hCoV-19/Egypt/ARMY-266/2021	84.02778	mediocre	EPI_ISL_1936126	29882	20D	C.36	O
hCoV-19/Egypt/ARMY-267/2021	100	bad	EPI_ISL_1936127	29879	20D	C.36.3	O
hCoV-19/Egypt/ARMY-275/2021	200.6944	bad	EPI_ISL_1936130	29891	19B	A.28	S
hCoV-19/Egypt/ARMY-231/2021	29.34028	good	EPI_ISL_1936134	29881	20D	C.36	GR
hCoV-19/Egypt/ARMY-203/2021	584.0278	bad	EPI_ISL_1936136	29881	20D	C.36	GR
hCoV-19/Egypt/ARMY-204/2021	584.0278	bad	EPI_ISL_1936137	29881	20D	C.36	GR
hCoV-19/Egypt/ARMY-205/2021	584.0278	bad	EPI_ISL_1936138	29881	20D	C.36	GR
hCoV-19/Egypt/ARMY-207/2021	544.4444	bad	EPI_ISL_1936139	29878	20D	C.36	GR
hCoV-19/Egypt/ARMY-211/2021	544.4444	bad	EPI_ISL_1936140	29878	20D	C.36	GR
hCoV-19/Egypt/ARMY-212/2021	39.0625	mediocre	EPI_ISL_1936141	29887	20A	B.1	GH
hCoV-19/Egypt/ARMY-215/2021	39.0625	mediocre	EPI_ISL_1936142	29882	20A	B.1	GH
hCoV-19/Egypt/ARMY-216/2021	39.0625	mediocre	EPI_ISL_1936143	29880	20A	B.1	GH
hCoV-19/Egypt/ARMY-217/2021	584.0278	bad	EPI_ISL_1936144	29881	20D	C.36	GR
hCoV-19/Egypt/ARMY-218/2021	544.4444	bad	EPI_ISL_1936145	29878	20D	C.36	GR
hCoV-19/Egypt/ARMY-20/2021	39.0625	mediocre	EPI_ISL_1936194	29884	20D	C.36	GR
hCoV-19/Egypt/ARMY-21/2021	69.44444	mediocre	EPI_ISL_1936195	29878	20D	C.36	GR
hCoV-19/Egypt/ARMY-22/2021	146.0069	bad	EPI_ISL_1936196	29878	20D	C.38	GR
hCoV-19/Egypt/ARMY-23/2021	21.00694	good	EPI_ISL_1936197	29880	20A	B.1	GH
hCoV-19/Egypt/ARMY-24/2021	21.00694	good	EPI_ISL_1936198	29880	20A	B.1	GH
hCoV-19/Egypt/ARMY-25/2021	21.00694	good	EPI_ISL_1936199	29877	20A	B.1	GH
hCoV-19/Egypt/ARMY-26/2021	21.00694	good	EPI_ISL_1936200	29880	20I (Alpha, V1)	None	GR
hCoV-19/Egypt/ARMY-27/2021	21.00694	good	EPI_ISL_1936201	29884	20I (Alpha, V1)	None	GR
hCoV-19/Egypt/ARMY-28/2021	21.00694	good	EPI_ISL_1936202	29880	20I (Alpha, V1)	None	GR
hCoV-19/Egypt/ARMY-29/2021	21.00694	good	EPI_ISL_1936203	29879	20I (Alpha, V1)	None	GR
hCoV-19/Egypt/ARMY-30/2021	84.02778	mediocre	EPI_ISL_1936204	29884	20D	C.36	GR
hCoV-19/Egypt/ARMY-31/2021	84.02778	mediocre	EPI_ISL_1936205	29879	20D	C.36	GR
hCoV-19/Egypt/ARMY-32/2021	84.02778	mediocre	EPI_ISL_1936206	29884	20D	C.36	GR
hCoV-19/Egypt/ARMY-33/2021	84.02778	mediocre	EPI_ISL_1936207	29878	20D	C.36	GR
hCoV-19/Egypt/ARMY-34/2021	84.02778	mediocre	EPI_ISL_1936208	29884	20D	C.36	GR
hCoV-19/Egypt/ARMY-63/2021	84.02778	mediocre	EPI_ISL_1936209	29881	20D	C.36	GR
hCoV-19/Egypt/ARMY-35/2021	84.02778	mediocre	EPI_ISL_1936210	29879	20D	C.36	GR
hCoV-19/Egypt/ARMY-36/2021	84.02778	mediocre	EPI_ISL_1936211	29881	20D	C.36	GR
hCoV-19/Egypt/ARMY-37/2021	84.02778	mediocre	EPI_ISL_1936212	29877	20D	C.36	GR
hCoV-19/Egypt/ARMY-38/2021	84.02778	mediocre	EPI_ISL_1936213	29884	20D	C.36	GR
hCoV-19/Egypt/ARMY-39/2021	84.02778	mediocre	EPI_ISL_1936214	29876	20D	C.36	O
hCoV-19/Egypt/ARMY-40/2021	84.02778	mediocre	EPI_ISL_1936215	29879	20D	C.36	GR
hCoV-19/Egypt/ARMY-41/2021	84.02778	mediocre	EPI_ISL_1936216	29883	20D	C.36	GR
hCoV-19/Egypt/ARMY-42/2021	84.02778	mediocre	EPI_ISL_1936217	29884	20D	C.36	GR

hCoV-19/Egypt/ARMY-43/2021	84.02778	mediocre	EPI_ISL_1936218	29882	20D	C.36	O
hCoV-19/Egypt/ARMY-44/2021	100	bad	EPI_ISL_1936219	29879	20D	C.36.3	O
hCoV-19/Egypt/ARMY-45/2021	200.6944	bad	EPI_ISL_1936220	29895	19B	A.28	S
hCoV-19/Egypt/ARMY-65/2021	200.6944	bad	EPI_ISL_1936221	29899	19B	A.28	S
hCoV-19/Egypt/ARMY-51/2021	94.44444	mediocre	EPI_ISL_1936227	29890	20D	C.36.3	GR
hCoV-19/Egypt/ARMY-52/2021	584.0278	bad	EPI_ISL_1936228	29881	20D	C.36	GR
hCoV-19/Egypt/ARMY-53/2021	584.0278	bad	EPI_ISL_1936229	29881	20D	C.36	GR
hCoV-19/Egypt/ARMY-54/2021	584.0278	bad	EPI_ISL_1936230	29881	20D	C.36	GR
hCoV-19/Egypt/ARMY-55/2021	544.4444	bad	EPI_ISL_1936231	29878	20D	C.36	GR
hCoV-19/Egypt/ARMY-56/2021	544.4444	bad	EPI_ISL_1936232	29878	20D	C.36	GR
hCoV-19/Egypt/ARMY-57/2021	39.0625	mediocre	EPI_ISL_1936233	29887	20A	B.1	GH
hCoV-19/Egypt/ARMY-58/2021	39.0625	mediocre	EPI_ISL_1936234	29882	20A	B.1	GH
hCoV-19/Egypt/ARMY-59/2021	39.0625	mediocre	EPI_ISL_1936235	29880	20A	B.1	GH
hCoV-19/Egypt/ARMY-60/2021	584.0278	bad	EPI_ISL_1936236	29881	20D	C.36	GR
hCoV-19/Egypt/ARMY-61/2021	544.4444	bad	EPI_ISL_1936237	29878	20D	C.36	GR
hCoV-19/Egypt/ARMY-300/2021	108.5069	bad	EPI_ISL_1936240	29878	20D	C.36.3	GR
hCoV-19/Egypt/ARMY-301/2021	108.5069	bad	EPI_ISL_1936241	29878	20D	C.36.3	GR
hCoV-19/Egypt/ARMY-303/2021	108.5069	bad	EPI_ISL_1936242	29877	20D	C.36.3	GR
hCoV-19/Egypt/ARMY-304/2021	108.5069	bad	EPI_ISL_1936243	29885	20D	C.36.3	GR
hCoV-19/Egypt/ARMY-305/2021	76.5625	mediocre	EPI_ISL_1936244	29882	20D	C.36.3	GR
hCoV-19/Egypt/ARMY-306/2021	76.5625	mediocre	EPI_ISL_1936245	29881	20D	C.36.3	GR
hCoV-19/Egypt/ARMY-307/2021	76.5625	mediocre	EPI_ISL_1936246	29881	20D	C.36.3	GR
hCoV-19/Egypt/ARMY-308/2021	400	bad	EPI_ISL_1936247	29880	20D	C.38	GR
hCoV-19/Egypt/ARMY-309/2021	400	bad	EPI_ISL_1936248	29879	20D	C.38	GR
hCoV-19/Egypt/ARMY-310/2021	200.6944	bad	EPI_ISL_1936249	29881	19B	A.28	S
hCoV-19/Egypt/ARMY-311/2021	200.6944	bad	EPI_ISL_1936250	29879	19B	A.28	S
hCoV-19/Egypt/ARMY-312/2021	200.6944	bad	EPI_ISL_1936251	29881	19B	A.28	S
hCoV-19/Egypt/ARMY-313/2021	100	bad	EPI_ISL_1936252	29884	20B	B.1.1	S
hCoV-19/Egypt/ARMY-314/2021	100	bad	EPI_ISL_1936253	29886	20B	B.1.1	S
hCoV-19/Egypt/ARMY-315/2021	156.25	bad	EPI_ISL_1936254	29882	20D	C.36	GR
hCoV-19/Egypt/ARMY-316/2021	156.25	bad	EPI_ISL_1936255	29879	20D	C.36	GR
hCoV-19/Egypt/ARMY-317/2021	156.25	bad	EPI_ISL_1936256	29875	20D	C.36	GR
hCoV-19/Egypt/ARMY-318/2021	156.25	bad	EPI_ISL_1936257	29885	19B	A.28	S
hCoV-19/Egypt/ARMY-319/2021	156.25	bad	EPI_ISL_1936258	29888	19B	A.28	S
hCoV-19/Egypt/ARMY-320/2021	321.0069	bad	EPI_ISL_1936259	29883	19A	B.1.1	O
hCoV-19/Egypt/ARMY-321/2021	321.0069	bad	EPI_ISL_1936260	29880	19A	B.1.1	O
hCoV-19/Egypt/ARMY-324/2021	91.84028	mediocre	EPI_ISL_1936261	29889	20D	C.38	GR
hCoV-19/Egypt/ARMY-325/2021	91.84028	mediocre	EPI_ISL_1936262	29882	20D	C.38	GR
hCoV-19/Egypt/ARMY-326/2021	91.84028	mediocre	EPI_ISL_1936263	29875	20D	C.38	GR
hCoV-19/Egypt/ARMY-327/2021	400	bad	EPI_ISL_1936264	29873	20D	C.38	GR
hCoV-19/Egypt/ARMY-328/2021	400	bad	EPI_ISL_1936265	29883	20D	C.38	GR
hCoV-19/Egypt/ARMY-329/2021	108.5069	bad	EPI_ISL_1936266	29877	20D	C.36.3	GR
hCoV-19/Egypt/ARMY-330/2021	108.5069	bad	EPI_ISL_1936267	29875	20D	C.36.3	GR

hCoV-19/Egypt/ARMY-331/2021	108.5069	bad	EPI_ISL_1936268	29880	20D	C.36.3	GR
hCoV-19/Egypt/ARMY-332/2021	108.5069	bad	EPI_ISL_1936269	29875	20D	C.36.3	GR
hCoV-19/Egypt/ARMY-333/2021	108.5069	bad	EPI_ISL_1936270	29878	20D	C.36.3	GR
hCoV-19/Egypt/ARMY-334/2021	108.5069	bad	EPI_ISL_1936271	29875	20D	C.36.3	GR
hCoV-19/Egypt/ARMY-335/2021	108.5069	bad	EPI_ISL_1936272	29885	20D	C.36.3	GR
hCoV-19/Egypt/ARMY-340/2021	39.0625	mediocre	EPI_ISL_1936273	29877	20D	C.36	GR
hCoV-19/Egypt/ARMY-342/2021	17.36111	good	EPI_ISL_1936274	29884	20D	C.36	GR
hCoV-19/Egypt/ARMY-343/2021	0.694444	good	EPI_ISL_1936275	29875	20D	C.36.3	GR
hCoV-19/Egypt/ARMY-344/2021	0.173611	good	EPI_ISL_1936276	29880	20I (Alpha, V1)	None	GR
hCoV-19/Egypt/ARMY-348/2021	200.6944	bad	EPI_ISL_1936278	29882	19B	A.28	S
hCoV-19/Egypt/ARMY-349/2021	200.6944	bad	EPI_ISL_1936279	29890	19B	A.28	S
hCoV-19/Egypt/ARMY-356/2021	21.00694	good	EPI_ISL_1936286	29878	20I (Alpha, V1)	None	GR
hCoV-19/Egypt/ARMY-357/2021	21.00694	good	EPI_ISL_1936287	29878	20A	B.1	GH
hCoV-19/Egypt/ARMY-358/2021	21.00694	good	EPI_ISL_1936288	29876	20A	B.1	GH
hCoV-19/Egypt/ARMY-359/2021	21.00694	good	EPI_ISL_1936289	29880	20A	B.1	GH
hCoV-19/Egypt/ARMY-360/2021	21.00694	good	EPI_ISL_1936290	29879	20A	B.1	GH
hCoV-19/Egypt/ARMY-361/2021	29.34028	good	EPI_ISL_1936291	29878	20D	C.36	GR
hCoV-19/Egypt/ARMY-362/2021	29.34028	good	EPI_ISL_1936292	29879	20D	C.36	GR
hCoV-19/Egypt/ARMY-363/2021	29.34028	good	EPI_ISL_1936293	29888	20D	C.36	GR
hCoV-19/Egypt/ARMY-364/2021	29.34028	good	EPI_ISL_1936294	29873	20D	C.36	GR
hCoV-19/Egypt/PHARCO-ARMY-36/2021	34.02778	mediocre	EPI_ISL_1936296	29793	20D	C.36	GR
hCoV-19/Egypt/PHARCO-ARMY-37/2021	78.0625	mediocre	EPI_ISL_1936297	29793	20D	C.36	GR
hCoV-19/Egypt/PHARCO-ARMY-38/2021	7.25	good	EPI_ISL_1936298	29793	20D	C.36	GR
hCoV-19/Egypt/PHARCO-ARMY-39/2021	101.5625	bad	EPI_ISL_1936299	29793	20D	C.36	GR
hCoV-19/Egypt/PHARCO-ARMY-40/2021	54.17361	mediocre	EPI_ISL_1936300	29793	20D	C.36	GR
hCoV-19/Egypt/PHARCO-ARMY-41/2021	45.44444	mediocre	EPI_ISL_1936301	29793	20D	C.36	GR
hCoV-19/Egypt/PHARCO-ARMY-42/2021	100	bad	EPI_ISL_1936302	29793	20A	B.1	O
hCoV-19/Egypt/PHARCO-ARMY-43/2021	169	bad	EPI_ISL_1936303	29793	20D	C.36	GR
hCoV-19/Egypt/PHARCO-ARMY-44/2021	34.02778	mediocre	EPI_ISL_1936304	29793	20D	C.36	GR
hCoV-19/Egypt/PHARCO-ARMY-45/2021	78.0625	mediocre	EPI_ISL_1936305	29793	20D	C.36	GR
hCoV-19/Egypt/PHARCO-ARMY-46/2021	7.25	good	EPI_ISL_1936306	29793	20D	C.36	GR
hCoV-19/Egypt/PHARCO-ARMY-47/2021	101.5625	bad	EPI_ISL_1936307	29793	20D	C.36	GR
hCoV-19/Egypt/PHARCO-ARMY-48/2021	54.17361	mediocre	EPI_ISL_1936308	29793	20D	C.36	GR
hCoV-19/Egypt/PHARCO-ARMY-49/2021	45.44444	mediocre	EPI_ISL_1936309	29793	20D	C.36	GR
hCoV-19/Egypt/PHARCO-ARMY-51/2021	169	bad	EPI_ISL_1936311	29793	20D	C.36	GR
hCoV-19/Egypt/PHARCO-ARMY-52/2021	34.02778	mediocre	EPI_ISL_1936312	29793	20D	C.36	GR
hCoV-19/Egypt/PHARCO-ARMY-53/2021	78.0625	mediocre	EPI_ISL_1936313	29793	20D	C.36	GR
hCoV-19/Egypt/PHARCO-ARMY-54/2021	7.25	good	EPI_ISL_1936314	29793	20D	C.36	GR
hCoV-19/Egypt/PHARCO-ARMY-55/2021	101.5625	bad	EPI_ISL_1936315	29793	20D	C.36	GR
hCoV-19/Egypt/PHARCO-ARMY-56/2021	54.17361	mediocre	EPI_ISL_1936316	29793	20D	C.36	GR
hCoV-19/Egypt/PHARCO-ARMY-57/2021	45.44444	mediocre	EPI_ISL_1936317	29793	20D	C.36	GR
hCoV-19/Egypt/PHARCO-ARMY-59/2021	169	bad	EPI_ISL_1936319	29793	20D	C.36	GR

hCoV-19/Egypt/PHARCO-ARMY-60/2021	34.02778	mediocre	EPI_ISL_1936320	29793	20D	C.36	GR
hCoV-19/Egypt/PHARCO-ARMY-61/2021	78.0625	mediocre	EPI_ISL_1936321	29793	20D	C.36	GR
hCoV-19/Egypt/PHARCO-ARMY-62/2021	7.25	good	EPI_ISL_1936322	29793	20D	C.36	GR
hCoV-19/Egypt/PHARCO-ARMY-63/2021	101.5625	bad	EPI_ISL_1936323	29793	20D	C.36	GR
hCoV-19/Egypt/PHARCO-ARMY-64/2021	54.17361	mediocre	EPI_ISL_1936324	29793	20D	C.36	GR
hCoV-19/Egypt/PHARCO-ARMY-65/2021	45.44444	mediocre	EPI_ISL_1936325	29793	20D	C.36	GR
hCoV-19/Egypt/PHARCO-ARMY-66/2021	100	bad	EPI_ISL_1936326	29793	20A	B.1	O
hCoV-19/Egypt/PHARCO-ARMY-67/2021	169	bad	EPI_ISL_1936327	29793	20D	C.36	GR
hCoV-19/Egypt/PHARCO-ARMY-68/2021	34.02778	mediocre	EPI_ISL_1936328	29793	20D	C.36	GR
hCoV-19/Egypt/PHARCO-ARMY-69/2021	78.0625	mediocre	EPI_ISL_1936329	29793	20D	C.36	GR
hCoV-19/Egypt/PHARCO-ARMY-70/2021	7.25	good	EPI_ISL_1936330	29793	20D	C.36	GR
hCoV-19/Egypt/PHARCO-ARMY-71/2021	101.5625	bad	EPI_ISL_1936331	29793	20D	C.36	GR
hCoV-19/Egypt/PHARCO-ARMY-72/2021	54.17361	mediocre	EPI_ISL_1936332	29793	20D	C.36	GR
hCoV-19/Egypt/PHARCO-ARMY-73/2021	45.44444	mediocre	EPI_ISL_1936333	29793	20D	C.36	GR
hCoV-19/Egypt/PHARCO-ARMY-74/2021	100	bad	EPI_ISL_1936334	29793	20A	B.1	O
hCoV-19/Egypt/PHARCO-ARMY-75/2021	169	bad	EPI_ISL_1936335	29793	20D	C.36	GR
hCoV-19/Egypt/PHARCO-ARMY-76/2021	34.02778	mediocre	EPI_ISL_1936336	29793	20D	C.36	GR
hCoV-19/Egypt/PHARCO-ARMY-77/2021	78.0625	mediocre	EPI_ISL_1936337	29793	20D	C.36	GR
hCoV-19/Egypt/PHARCO-ARMY-78/2021	7.25	good	EPI_ISL_1936338	29793	20D	C.36	GR
hCoV-19/Egypt/PHARCO-ARMY/2021	101.5625	bad	EPI_ISL_1936339	29793	20D	C.36	GR
hCoV-19/Egypt/PHARCO-ARMY-21/2021	1225	bad	EPI_ISL_1936340	29793	20A	B.1.1	G
hCoV-19/Egypt/PHARCO-ARMY-22/2021	292.8403	bad	EPI_ISL_1936341	29796	20D	C.36	GR
hCoV-19/Egypt/PHARCO-ARMY-23/2021	177.7778	bad	EPI_ISL_1936342	29799	20D	C.36	GR
hCoV-19/Egypt/PHARCO-ARMY-24/2021	156.25	bad	EPI_ISL_1936343	29796	20D	C.36	GR
hCoV-19/Egypt/PHARCO-ARMY-25/2021	43.0625	mediocre	EPI_ISL_1936344	29793	20A	B.1	GH
hCoV-19/Egypt/PHARCO-ARMY-26/2021	1.173611	good	EPI_ISL_1936345	29790	20D	C.36	GR
hCoV-19/Egypt/PHARCO-ARMY-27/2021	50.17361	mediocre	EPI_ISL_1936346	29793	20D	C.36	GR
hCoV-19/Egypt/PHARCO-ARMY-28/2021	0.173611	good	EPI_ISL_1936347	29793	20D	C.36	GR
hCoV-19/Egypt/PHARCO-ARMY-29/2021	39.0625	mediocre	EPI_ISL_1936348	29879	20A	B.1	GH
hCoV-19/Egypt/PHARCO-ARMY-30/2021	0.694444	good	EPI_ISL_1936349	29875	20D	C.36	GR
hCoV-19/Egypt/PHARCO-ARMY-31/2021	0.694444	good	EPI_ISL_1936350	29876	20D	C.36	GR
hCoV-19/Egypt/PHARCO-ARMY-32/2021	0.694444	good	EPI_ISL_1936351	29876	20D	C.36	GR
hCoV-19/Egypt/PHARCO-ARMY-33/2021	0.694444	good	EPI_ISL_1936352	29877	20D	C.36	GR
hCoV-19/Egypt/PHARCO-ARMY-34/2021	0.694444	good	EPI_ISL_1936353	29870	20D	C.36	GR
hCoV-19/Egypt/PHARCO-ARMY-35/2021	0.694444	good	EPI_ISL_1936354	29873	20D	C.36	GR
hCoV-19/Egypt/PHARCO-ARMY-91/2021	39.0625	mediocre	EPI_ISL_1936355	29880	20A	B.1	GH
hCoV-19/Egypt/PHARCO-ARMY-92/2021	0.694444	good	EPI_ISL_1936356	29880	20D	C.36	GR
hCoV-19/Egypt/PHARCO-ARMY-93/2021	0.694444	good	EPI_ISL_1936357	29872	20D	C.36	GR
hCoV-19/Egypt/PHARCO-ARMY-94/2021	0.694444	good	EPI_ISL_1936358	29876	20D	C.36	GR
hCoV-19/Egypt/PHARCO-ARMY-95/2021	0.694444	good	EPI_ISL_1936359	29877	20D	C.36	GR
hCoV-19/Egypt/PHARCO-ARMY-96/2021	0.694444	good	EPI_ISL_1936360	29874	20D	C.36	GR
hCoV-19/Egypt/PHARCO-ARMY-97/2021	0.694444	good	EPI_ISL_1936361	29880	20D	C.36	GR
hCoV-19/Egypt/PHARCO-ARMY-98/2021	39.0625	mediocre	EPI_ISL_1936362	29875	20A	B.1	GH

hCoV-19/Egypt/PHARCO-ARMY-99/2021	39.0625	mediocre	EPI_ISL_1936363	29877	20A	B.1	GH
hCoV-19/Egypt/PHARCO-ARMY-100/2021	0.694444	good	EPI_ISL_1936364	29880	20D	C.36	GR
hCoV-19/Egypt/PHARCO-ARMY-81/2021	39.0625	mediocre	EPI_ISL_1936365	29880	20A	B.1	GH
hCoV-19/Egypt/PHARCO-ARMY-82/2021	0.694444	good	EPI_ISL_1936366	29880	20D	C.36	GR
hCoV-19/Egypt/PHARCO-ARMY-83/2021	0.694444	good	EPI_ISL_1936367	29872	20D	C.36	GR
hCoV-19/Egypt/PHARCO-ARMY-84/2021	0.694444	good	EPI_ISL_1936368	29876	20D	C.36	GR
hCoV-19/Egypt/PHARCO-ARMY-85/2021	0.694444	good	EPI_ISL_1936369	29877	20D	C.36	GR
hCoV-19/Egypt/PHARCO-ARMY-86/2021	0.694444	good	EPI_ISL_1936370	29874	20D	C.36	GR
hCoV-19/Egypt/PHARCO-ARMY-87/2021	0.694444	good	EPI_ISL_1936371	29880	20D	C.36	GR
hCoV-19/Egypt/PHARCO-ARMY-88/2021	39.0625	mediocre	EPI_ISL_1936372	29875	20A	B.1	GH
hCoV-19/Egypt/PHARCO-ARMY-89/2021	39.0625	mediocre	EPI_ISL_1936373	29877	20A	B.1	GH
hCoV-19/Egypt/PHARCO-ARMY-80/2021	0.694444	good	EPI_ISL_1936374	29880	20D	C.36	GR
hCoV-19/Egypt/ARMY-400/2021	0	good	EPI_ISL_1969078	29783	20I (Alpha, V1)	B.1.1.7	GR
hCoV-19/Egypt/ARMY-401/2021	0	good	EPI_ISL_1969079	29783	20I (Alpha, V1)	B.1.1.7	GR
hCoV-19/Egypt/ARMY-402/2021	0	good	EPI_ISL_1969080	29783	20I (Alpha, V1)	B.1.1.7	GR
hCoV-19/Egypt/CCHE57357_Wave_3_A004/2021	11.11111	good	EPI_ISL_2566470	29903	20D	C.36.3	GR
hCoV-19/Egypt/CCHE57357_Wave_3_A039/2021	25	good	EPI_ISL_2566492	29903	20D	C.36.3	GR
hCoV-19/Egypt/CCHE57357_Wave_3_A051/2021	0	good	EPI_ISL_2566500	29903	20D	C.36.3	GR
hCoV-19/Egypt/NRC-481/2021	71.52553	mediocre	EPI_ISL_2227328	29894	20D	C.36.3	GR
hCoV-19/Egypt/NRC-479/2021	14.52182	good	EPI_ISL_2227329	29891	20D	C.36.3	GR
hCoV-19/Egypt/NRC-515/2021	108.3685	bad	EPI_ISL_2227331	29878	20D	C.36.3	GR
hCoV-19/Egypt/NRC-415/2021	14.66996	good	EPI_ISL_2227332	29888	20D	C.36.3	GR
hCoV-19/Egypt/NRC-431/2021	431.056	bad	EPI_ISL_2227333	29881	20D	C.36	GR
hCoV-19/Egypt/NRC-440/2021	12.43215	good	EPI_ISL_2227334	29880	20D	C.36.3	GR
hCoV-19/Egypt/NRC-291/2021	7.695487	good	EPI_ISL_2227335	29886	20D	C.36.3	GR
hCoV-19/Egypt/NRC-308/2021	387.717	bad	EPI_ISL_2227336	29051	20D	C.36	GR
hCoV-19/Egypt/NRC-465/2021	18.03293	good	EPI_ISL_2227337	29894	20D	C.36.3	GR
hCoV-19/Egypt/NRC-435/2021	3.498285	good	EPI_ISL_2227338	29892	20D	C.36.3	GR
hCoV-19/Egypt/NRC-434/2021	3.30701	good	EPI_ISL_2227339	29896	20D	C.36.3	GR
hCoV-19/Egypt/NRC-335/2021	401.9808	bad	EPI_ISL_2227340	29898	20D	C.36	GR
hCoV-19/Egypt/NRC-369/2021	1.949643	good	EPI_ISL_2227341	29894	20D	C.36.3	GR
hCoV-19/Egypt/NRC-433/2021	0.98524	good	EPI_ISL_2227342	29888	20D	C.36.3	GR
hCoV-19/Egypt/NRC-407/2021	292.2556	bad	EPI_ISL_2227343	29901	20D	C.36	GR
hCoV-19/Egypt/NRC-518/2020	49.6242	mediocre	EPI_ISL_2227344	29886	20D	C.36.3	GR
hCoV-19/Egypt/NRC-314/2021	20.28335	good	EPI_ISL_2227346	29893	20D	C.36.3	GR
hCoV-19/Egypt/NRC-337/2021	19.52331	good	EPI_ISL_2227347	29880	20D	C.36.3	GR
hCoV-19/Egypt/NRC-287/2021	1.5625	good	EPI_ISL_2227348	29892	20D	C.36.3	GR
hCoV-19/Egypt/NRC-488/2021	0	good	EPI_ISL_2227350	29890	20D	C.36.3	GR
hCoV-19/Egypt/NRC-6177/2020	63.32339	mediocre	EPI_ISL_2232253	29873	20A	B.1	GH
hCoV-19/Egypt/NRC-6638/2020	239.0625	bad	EPI_ISL_2232254	29868	20A	B.1	G
hCoV-19/Egypt/NRC-6166/2020	56.25	mediocre	EPI_ISL_2232255	29872	20D	C.36	GR

hCoV-19/Egypt/NRC-6143/2020	70.3125	mediocre	EPI_ISL_2232256	29879	20A	B.1	G
hCoV-19/Egypt/NRC-6632/2020	95.3125	mediocre	EPI_ISL_2232257	29893	20D	C.36	GR
hCoV-19/Egypt/NRC-6737/2020	95.3125	mediocre	EPI_ISL_2232258	29856	20D	C.36	GR
hCoV-19/Egypt/NRC-5876/2020	73.61111	mediocre	EPI_ISL_2232259	29876	20A	B.1.170	GH
hCoV-19/Egypt/NRC-6883/2020	64.75694	mediocre	EPI_ISL_2232260	29878	20D	C.36	GR
hCoV-19/Egypt/NRC-6264/2020	59.02778	mediocre	EPI_ISL_2232262	29878	20D	C.36	GR
hCoV-19/Egypt/NRC-6369/2020	57.8125	mediocre	EPI_ISL_2232263	29867	20D	C.36	GR
hCoV-19/Egypt/NRC-6089/2020	62.5	mediocre	EPI_ISL_2232264	29903	20D	C.36	GR
hCoV-19/Egypt/NRC-6000/2020	60.59028	mediocre	EPI_ISL_2232265	29865	20D	C.36	GR
hCoV-19/Egypt/NRC-6015/2020	60.59028	mediocre	EPI_ISL_2232266	29865	20D	C.36	GR
hCoV-19/Egypt/NRC-6943/2020	70.3125	mediocre	EPI_ISL_2232270	29878	20B	B.1.1.31 2	GR
hCoV-19/Egypt/NRC-6186/2020	57.8125	mediocre	EPI_ISL_2232271	29878	20A	B.1	GH
hCoV-19/Egypt/NRC-6069/2020	56.94444	mediocre	EPI_ISL_2232272	29878	20A	B.1.544	GH
hCoV-19/Egypt/NRC-6839/2020	70.3125	mediocre	EPI_ISL_2232273	29878	20A	B.1	GH
hCoV-19/Egypt/NRC-7011/2020	70.3125	mediocre	EPI_ISL_2232274	29873	20A	B.1	GH
hCoV-19/Egypt/NRC-6375/2020	56.25	mediocre	EPI_ISL_2232276	29902	20D	C.36	GR
hCoV-19/Egypt/NRC-6317/2020	164.7569	bad	EPI_ISL_2232278	29902	20D	B.1	G
hCoV-19/Egypt/NRC-6168/2020	73.61111	mediocre	EPI_ISL_2232279	29878	20D	B.1.1.1	GR
hCoV-19/Egypt/NRC-6181/2020	57.8125	mediocre	EPI_ISL_2232280	29878	20A	B.1	GH
hCoV-19/Egypt/NRC-6180/2020	64.75694	mediocre	EPI_ISL_2232281	29869	20A	B.1	GH
hCoV-19/Egypt/NRC-6179/2020	62.5	mediocre	EPI_ISL_2232282	29879	20A	B.1	GH
hCoV-19/Egypt/NRC-6141/2020	59.02778	mediocre	EPI_ISL_2232283	29895	20A	B.1	GH
hCoV-19/Egypt/NRC-6051/2020	70.3125	mediocre	EPI_ISL_2232285	29886	20A	B.1	GH
hCoV-19/Egypt/NRC-5900/2020	59.02778	mediocre	EPI_ISL_2232286	29893	20A	B.1	GH
hCoV-19/Egypt/NRC-5980/2020	106.4236	bad	EPI_ISL_2232287	29901	20A	B.1	GH
hCoV-19/Egypt/NRC-5911/2020	59.02778	mediocre	EPI_ISL_2232289	29898	20A	B.1	GH
hCoV-19/Egypt/NRC-5880/2020	56.94444	mediocre	EPI_ISL_2232290	29892	20A	B.1.170	GH
hCoV-19/Egypt/NRC-5623/2020	56.25	mediocre	EPI_ISL_2232292	29897	20D	C.36	GR
hCoV-19/Egypt/NRC-5912/2020	81.25	mediocre	EPI_ISL_2232293	29899	20A	B.1	GH
hCoV-19/Egypt/NRC-6001/2020	0	good	EPI_ISL_2232294	29902	20D	C.36	GR
hCoV-19/Egypt/NRC-5990/2020	4.340278	good	EPI_ISL_2232295	29867	20A	B.1.195	G
hCoV-19/Egypt/NRC-5882/2020	14.0625	good	EPI_ISL_2232296	29903	20A	B.1.170	GH
hCoV-19/Egypt/NRC-6076/2020	2.777778	good	EPI_ISL_2232297	29890	20A	B.1	GH
hCoV-19/Egypt/NRC-6021/2020	2.777778	good	EPI_ISL_2232298	29897	20A	B.1	GH
hCoV-19/Egypt/NRC-5886/2020	0	good	EPI_ISL_2232301	29899	20B	B.1.1	GR
hCoV-19/Egypt/NRC-5467/2020	0.694444	good	EPI_ISL_2232302	29883	20B	B.1.1	GR
hCoV-19/Egypt/NRC-6117/2020	11.11111	good	EPI_ISL_2232303	29866	20A	B.1.170	GH
hCoV-19/Egypt/NRC-6837/2020	0.694444	good	EPI_ISL_2232304	29901	20A	B.1	GH
hCoV-19/Egypt/NRC-6834/2020	14.0625	good	EPI_ISL_2232305	29902	20D	C.36	GR
hCoV-19/Egypt/NRC-6828/2020	0	good	EPI_ISL_2232307	29903	20A	B.1	GH
hCoV-19/Egypt/NRC-6504/2020	8.506944	good	EPI_ISL_2232310	29903	20D	C.36	GR
hCoV-19/Egypt/NRC-6373/2020	0	good	EPI_ISL_2232311	29903	20D	C.36	GR
hCoV-19/Egypt/NRC-6374/2020	0	good	EPI_ISL_2232312	29902	20D	C.36	GR

hCoV-19/Egypt/NRC-5586/2020	0.173611	good	EPI_ISL_2232316	29878	20A	B.1	GH
hCoV-19/Egypt/NRC-5587/2020	0	good	EPI_ISL_2232317	29902	20A	B.1	GH
hCoV-19/Egypt/NRC-6012/2020	1.5625	good	EPI_ISL_2232318	29903	20B	B.1.1	GR
hCoV-19/Egypt/NRC-5890/2020	0.694444	good	EPI_ISL_2232320	29902	20A	B.1.195	G
hCoV-19/Egypt/NRC-5864/2020	0.694444	good	EPI_ISL_2232322	29902	20A	B.1.170	GH
hCoV-19/Egypt/NRC-5799/2020	0	good	EPI_ISL_2232323	29878	20A	B.1	G
hCoV-19/Egypt/NRC-6895/2020	0.173611	good	EPI_ISL_2232324	29901	20D	C.36	GR
hCoV-19/Egypt/NRC-5455/2020	0	good	EPI_ISL_2232326	29900	20D	C.36	GR
hCoV-19/Egypt/NRC-5447/2020	0	good	EPI_ISL_2232329	29903	20D	C.36	GR
hCoV-19/Egypt/NRC-6161/2020	0	good	EPI_ISL_2232336	29903	20D	C.36	GR
hCoV-19/Egypt/NRC-6160/2020	0	good	EPI_ISL_2232337	29900	20D	C.36	GR
hCoV-19/Egypt/NRC-5548/2020	0	good	EPI_ISL_2232338	29901	20D	C.36	GR
hCoV-19/Egypt/NRC-6004/2020	0.173611	good	EPI_ISL_2232339	29903	20A	B.1	G
hCoV-19/Egypt/NRC-5797/2020	0	good	EPI_ISL_2232340	29903	20A	B.1	GH
hCoV-19/Egypt/NRC-5767/2020	0	good	EPI_ISL_2232342	29902	20A	B.1.398	G
hCoV-19/Egypt/NRC-5462/2020	1.5625	good	EPI_ISL_2232344	29903	20B	B.1.1	GR
hCoV-19/Egypt/NRC-462/2020	2.777778	good	EPI_ISL_2232345	29903	20B	B.1.1	GR
hCoV-19/Egypt/NRC-5444/2020	0	good	EPI_ISL_2232347	29903	20D	C.36	GR
hCoV-19/Egypt/NRC-5445/2020	0	good	EPI_ISL_2232348	29903	20D	C.36	GR
hCoV-19/Egypt/NRC-5442/2020	0.173611	good	EPI_ISL_2232349	29867	20D	C.36	GR
hCoV-19/Egypt/NRC-6838/2020	0.173611	good	EPI_ISL_2232350	29903	20A	B.1	GH
hCoV-19/Egypt/NRC-6841/2020	2.777778	good	EPI_ISL_2232351	29896	20A	B.1	GH
hCoV-19/Egypt/NRC-7021/2020	8.506944	good	EPI_ISL_2232352	29900	20A	B.1	GH
hCoV-19/Egypt/NRC-6976/2020	6.25	good	EPI_ISL_2232353	29900	20A	B.1	GH
hCoV-19/Egypt/NRC-6959/2020	4.340278	good	EPI_ISL_2232355	29899	20A	B.1	GH
hCoV-19/Egypt/NRC-6942/2020	8.506944	good	EPI_ISL_2232356	29901	20A	B.1	GH
hCoV-19/Egypt/NRC-6630/2020	2.777778	good	EPI_ISL_2232357	29878	20A	B.1.195	G
hCoV-19/Egypt/NRC-6823/2020	0.173611	good	EPI_ISL_2232358	29902	20A	B.1	GH
hCoV-19/Egypt/NRC-6821/2020	0.694444	good	EPI_ISL_2232360	29903	20A	B.1	GH
hCoV-19/Egypt/NRC-6820/2020	0.173611	good	EPI_ISL_2232361	29903	20A	B.1	GH
hCoV-19/Egypt/NRC-6818/2020	0.173611	good	EPI_ISL_2232362	29903	20A	B.1	GH
hCoV-19/Egypt/NRC-6819/2020	0.694444	good	EPI_ISL_2232363	29903	20A	B.1	GH
hCoV-19/Egypt/NRC-6816/2020	0.173611	good	EPI_ISL_2232364	29902	20A	B.1	GH
hCoV-19/Egypt/NRC-6817/2020	0.694444	good	EPI_ISL_2232365	29902	20A	B.1	GH
hCoV-19/Egypt/NRC-6760/2020	8.506944	good	EPI_ISL_2232366	29903	20A	B.1	GH
hCoV-19/Egypt/NRC-6465/2020	1.5625	good	EPI_ISL_2232367	29893	20D	C.36	G
hCoV-19/Egypt/NRC-6241/2020	0	good	EPI_ISL_2232369	29888	20D	C.36	GR
hCoV-19/Egypt/NRC-6217/2020	0	good	EPI_ISL_2232371	29896	20D	C.36	GR
hCoV-19/Egypt/NRC-6218/2020	0	good	EPI_ISL_2232372	29899	20D	C.36	GR
hCoV-19/Egypt/NRC-5638/2020	0	good	EPI_ISL_2232373	29891	20A	B.1.170	GH
hCoV-19/Egypt/NRC-5641/2020	0.694444	good	EPI_ISL_2232374	29901	20A	B.1.170	GH
hCoV-19/Egypt/NRC-5628/2020	4.340278	good	EPI_ISL_2232375	29903	20B	B.1.1	GR
hCoV-19/Egypt/NRC-5630/2020	1.5625	good	EPI_ISL_2232376	29881	20B	B.1.1	GR

hCoV-19/Egypt/NRC-5646/2020	0	good	EPI_ISL_2232377	29903	20A	B.1.170	GH
hCoV-19/Egypt/NRC-5648/2020	0	good	EPI_ISL_2232378	29903	20A	B.1.170	GH
hCoV-19/Egypt/NRC-5626/2020	2.777778	good	EPI_ISL_2232380	29901	20B	B.1.1	GR
hCoV-19/Egypt/NRC-6158/2020	0	good	EPI_ISL_2232381	29893	20D	C.36	GR
hCoV-19/Egypt/NRC-6071/2020	0	good	EPI_ISL_2232383	29903	20D	C.36	GR
hCoV-19/Egypt/NRC-6048/2020	1.5625	good	EPI_ISL_2232384	29891	20A	B.1	GH
hCoV-19/Egypt/NRC-5983/2020	1.5625	good	EPI_ISL_2232385	29903	20A	B.1	GH
hCoV-19/Egypt/NRC-5984/2020	4.340278	good	EPI_ISL_2232386	29902	20A	B.1	GH
hCoV-19/Egypt/NRC-6025/2020	0.173611	good	EPI_ISL_2232387	29898	20A	B.1.170	GH
hCoV-19/Egypt/NRC-5981/2020	1.5625	good	EPI_ISL_2232388	29902	20A	B.1	GH
hCoV-19/Egypt/NRC-5909/2020	6.25	good	EPI_ISL_2232389	29893	20A	B.1	GH
hCoV-19/Egypt/NRC-5892/2020	0.694444	good	EPI_ISL_2232390	29903	20A	B.1.195	G
hCoV-19/Egypt/NRC-5879/2020	1.5625	good	EPI_ISL_2232391	29902	20A	B.1.170	GH
hCoV-19/Egypt/NRC-5877/2020	0.173611	good	EPI_ISL_2232392	29866	20A	B.1.170	GH
hCoV-19/Egypt/NRC-5844/2020	0.694444	good	EPI_ISL_2232393	29899	20A	B.1	GH
hCoV-19/Egypt/NRC-5878/2020	0.694444	good	EPI_ISL_2232394	29866	20A	B.1.170	GH
hCoV-19/Egypt/NRC-5884/2020	0.173611	good	EPI_ISL_2232396	29903	20A	B.1.170	GH
hCoV-19/Egypt/NRC-5863/2020	11.11111	good	EPI_ISL_2232397	29903	20D	C.36	GR
hCoV-19/Egypt/NRC-5871/2020	0.173611	good	EPI_ISL_2232399	29902	20A	B.1.170	GH
hCoV-19/Egypt/NRC-5872/2020	8.506944	good	EPI_ISL_2232400	29902	20A	B.1.170	GH
hCoV-19/Egypt/NRC-5874/2020	0.694444	good	EPI_ISL_2232401	29902	20A	B.1.170	GH
hCoV-19/Egypt/NRC-5875/2020	0.694444	good	EPI_ISL_2232402	29902	20A	B.1.170	GH
hCoV-19/Egypt/NRC-5781/2020	2.777778	good	EPI_ISL_2232403	29902	20A	B.1.170	GH
hCoV-19/Egypt/NRC-5520/2020	1.5625	good	EPI_ISL_2232404	29903	20B	B.1.1	GR
hCoV-19/Egypt/NRC-463/2020	4.340278	good	EPI_ISL_2232406	29903	20A	B.1.170	GH
hCoV-19/Egypt/CPHL-NRC-S23/2021	189.9292	bad	EPI_ISL_2313046	29632	20D	C.36.3	GR
hCoV-19/Egypt/CPHL-NRC-S22/2021	174.1442	bad	EPI_ISL_2313047	29881	20D	C.36.3	GR
hCoV-19/Egypt/CPHL-NRC-S21/2021	37.39096	mediocre	EPI_ISL_2313048	29880	20D	C.36.3	GR
hCoV-19/Egypt/CPHL-NRC-S19/2021	100.5192	bad	EPI_ISL_2313050	29631	20D	C.36.3	GR
hCoV-19/Egypt/CPHL-NRC-S17/2021	12.25	good	EPI_ISL_2313052	28316	20D	C.36.3	GR
hCoV-19/Egypt/CPHL-NRC-S16/2021	23.50454	good	EPI_ISL_2313053	29742	20D	C.36.3	GR
hCoV-19/Egypt/CPHL-NRC-S14/2021	35.38047	mediocre	EPI_ISL_2313055	29732	20D	C.36.3	GR
hCoV-19/Egypt/CPHL-NRC-S12/2021	56.70173	mediocre	EPI_ISL_2313057	29472	20D	C.36.3	GR
hCoV-19/Egypt/CPHL-NRC-S11/2021	319.9315	bad	EPI_ISL_2313058	29888	20D	C.36	GR
hCoV-19/Egypt/CPHL-NRC-S10/2021	16.62827	good	EPI_ISL_2313059	29731	20D	C.36.3	GR
hCoV-19/Egypt/CPHL-NRC-S9/2021	315.6544	bad	EPI_ISL_2313060	29885	20D	C.36.3	GR
hCoV-19/Egypt/CPHL-NRC-S8/2021	410.4305	bad	EPI_ISL_2313061	29752	20D	C.36	GR
hCoV-19/Egypt/CPHL-NRC-S7/2021	3.896968	good	EPI_ISL_2313062	29726	20D	C.36.3	GR
hCoV-19/Egypt/CPHL-NRC-S6/2021	98.15672	mediocre	EPI_ISL_2313063	29879	20D	C.36.3	GR
hCoV-19/Egypt/CPHL-NRC-S4/2021	146.2308	bad	EPI_ISL_2313065	29879	20D	C.36.3	GR
hCoV-19/Egypt/CPHL-NRC-S3/2021	51.30804	mediocre	EPI_ISL_2313066	29734	20D	C.36.3	GR
hCoV-19/Egypt/CPHL-NRC-S2/2021	46.74508	mediocre	EPI_ISL_2313067	29892	20D	C.36.3	GR
hCoV-19/Egypt/CPHL-NRC-S1/2021	4.394458	good	EPI_ISL_2313068	29588	20D	C.36.3	GR

hCoV-19/Egypt/CPHL-NRC-S24/2021	116.56	bad	EPI_ISL_2313069	29880	20D	C.36.3	GR
hCoV-19/Egypt/NRC-6407/2020	1542.638	bad	EPI_ISL_2380050	29865	20D	C.36	GR
hCoV-19/Egypt/NRC-6182/2020	121.0069	bad	EPI_ISL_2380051	29874	20A	B.1	GH
hCoV-19/Egypt/NRC-6408/2020	60.0625	mediocre	EPI_ISL_2380052	29880	20D	C.36	GR
hCoV-19/Egypt/NRC-6631/2020	1939.138	bad	EPI_ISL_2380053	29857	20D	C.36	GR
hCoV-19/Egypt/NRC-6856/2020	134.1004	bad	EPI_ISL_2380054	29852	20A	B.1.470	GH
hCoV-19/Egypt/NRC-6853/2020	575.0447	bad	EPI_ISL_2380055	29866	20A	B.1.170	GH
hCoV-19/Egypt/NRC-6176/2020	285.5903	bad	EPI_ISL_2380056	29879	20A	B.1	GH
hCoV-19/Egypt/NRC-6634/2020	56.25	mediocre	EPI_ISL_2380057	29900	20D	C.36	GR
hCoV-19/Egypt/NRC-6849/2020	1840.73	bad	EPI_ISL_2380058	29865	20A	B.1	GH
hCoV-19/Egypt/NRC-6170/2020	545.3125	bad	EPI_ISL_2380059	29867	20A	B.1	GH
hCoV-19/Egypt/NRC-6848/2020	249.7878	bad	EPI_ISL_2380060	29875	20A	B.1	GH
hCoV-19/Egypt/NRC-6663/2020	325	bad	EPI_ISL_2380061	29876	20A	B.1	G
hCoV-19/Egypt/NRC-6651/2020	254.3403	bad	EPI_ISL_2380062	29876	20A	B.1	GH
hCoV-19/Egypt/NRC-6185/2020	56.94444	mediocre	EPI_ISL_2380063	29879	20A	B.1	GH
hCoV-19/Egypt/NRC-6165/2020	59.02778	mediocre	EPI_ISL_2380064	29877	20D	C.36	GR
hCoV-19/Egypt/NRC-6840/2020	77.25694	mediocre	EPI_ISL_2380065	29895	20A	B.1	GH
hCoV-19/Egypt/NRC-6649/2020	156.9444	bad	EPI_ISL_2380066	29867	20A	B.1	G
hCoV-19/Egypt/NRC-6184/2020	234.8767	bad	EPI_ISL_2380067	29879	20A	B.1	GH
hCoV-19/Egypt/NRC-6183/2020	116.8403	bad	EPI_ISL_2380068	29880	20A	B.1	GH
hCoV-19/Egypt/NRC-6581/2020	254.3403	bad	EPI_ISL_2380069	29864	20A	B.1	GH
hCoV-19/Egypt/NRC-6746/2020	259.3364	bad	EPI_ISL_2380070	29882	20A	B.1	GH
hCoV-19/Egypt/NRC-6781/2020	1237.638	bad	EPI_ISL_2380071	29874	20A	B.1	G
hCoV-19/Egypt/NRC-6751/2020	967.3611	bad	EPI_ISL_2380072	29877	20A	B.1	GH
hCoV-19/Egypt/NRC-5868/2020	1773.611	bad	EPI_ISL_2380073	29905	20B	B.1.1	GR
hCoV-19/Egypt/NRC-6775/2020			EPI_ISL_2380074	29852	None	B.1.385	GH
hCoV-19/Egypt/NRC-6050/2020	9643.961	bad	EPI_ISL_2380075	29879	20A	B.1.606	GH
hCoV-19/Egypt/NRC-6773/2020	2609.969	bad	EPI_ISL_2380076	29873	20A	B.1.177	G
hCoV-19/Egypt/NRC-6003/2020	59.02778	mediocre	EPI_ISL_2380077	29904	20D	C.36	GR
hCoV-19/Egypt/NRC-6761/2020	65.35614	mediocre	EPI_ISL_2380078	29847	20A	B.1.470	GH
hCoV-19/Egypt/NRC-6037/2020	77.25694	mediocre	EPI_ISL_2380079	29866	20A	B.1.195	G
hCoV-19/Egypt/NRC-6766/2020	1467.373	bad	EPI_ISL_2380080	29874	20A	B.1	G
hCoV-19/Egypt/NRC-6102/2020	301.5625	bad	EPI_ISL_2380081	29884	20B	C.8	GR
hCoV-19/Egypt/NRC-5918/2020	109.2137	bad	EPI_ISL_2380082	29902	20A	B.1	GH
hCoV-19/Egypt/NRC-6378/2020	85.59028	mediocre	EPI_ISL_2380083	29863	20A	B.1.170	GH
hCoV-19/Egypt/NRC-6513/2020	351.5625	bad	EPI_ISL_2380084	29864	20D	C.36	GR
hCoV-19/Egypt/NRC-6082/2020	121.0069	bad	EPI_ISL_2380085	29876	20A	B.1	GH
hCoV-19/Egypt/NRC-5972/2020	550.756	bad	EPI_ISL_2380086	29862	20A	B.1.170	GH
hCoV-19/Egypt/NRC-6596/2020	118.9236	bad	EPI_ISL_2380087	29828	20A	B.1	GH
hCoV-19/Egypt/NRC-6030/2020	81.25	mediocre	EPI_ISL_2380088	29865	20A	B.1.170	GH
hCoV-19/Egypt/NRC-6743/2020	556.4236	bad	EPI_ISL_2380089	29862	20A	B.1	G
hCoV-19/Egypt/NRC-6792/2020	392.3098	bad	EPI_ISL_2380090	29861	20A	B.1	GH
hCoV-19/Egypt/NRC-5883/2020	56.94444	mediocre	EPI_ISL_2380091	29904	20A	B.1.170	GH

hCoV-19/Egypt/NRC-6478/2020	84.02778	mediocre	EPI_ISL_2380092	29902	20D	B.1.1	G
hCoV-19/Egypt/NRC-6496/2020	0.694444	good	EPI_ISL_2380093	29901	20D	C.36	GR
hCoV-19/Egypt/NRC-6639/2020	67.36111	mediocre	EPI_ISL_2380094	29869	20A	B.1	GH
hCoV-19/Egypt/NRC-6503/2020	77.25694	mediocre	EPI_ISL_2380095	29867	20D	C.36	GR
hCoV-19/Egypt/NRC-6637/2020	77.25694	mediocre	EPI_ISL_2380096	29879	20A	B.1	G
hCoV-19/Egypt/NRC-6599/2020	9322.645	bad	EPI_ISL_2380097	29881	20A	B.1.1.372	G
hCoV-19/Egypt/NRC-6572/2020	881.3773	bad	EPI_ISL_2380098	29864	20B	B.1.1.184	GR
hCoV-19/Egypt/NRC-289/2021	58.69285	mediocre	EPI_ISL_2227330	29825	20D	C.36.3	GR
hCoV-19/Egypt/NRC-295/2021	27.3124	good	EPI_ISL_2227345	29879	20D	C.36.3	GR
hCoV-19/Egypt/NRC-329/2021	0	good	EPI_ISL_2227349	29887	20D	C.36.3	GR
hCoV-19/Egypt/EMC-2/2021	0	good	EPI_ISL_2955336	29756	20I (Alpha, V1)	B.1.1.7	GRY
hCoV-19/Egypt/EMC-1/2021	0.64	good	EPI_ISL_2960150	29148	20I (Alpha, V1)	B.1.1.7	GRY
hCoV-19/Egypt/ARMY-273/2021	200.6944	bad	EPI_ISL_1936128	29895	19B	A.28	S
hCoV-19/Egypt/ARMY-274/2021	200.6944	bad	EPI_ISL_1936129	29899	19B	A.28	S
hCoV-19/Egypt/ARMY-297/2021	94.44444	mediocre	EPI_ISL_1936135	29890	20D	C.36.3	GR
hCoV-19/Egypt/ARMY-46/2021	200.6944	bad	EPI_ISL_1936222	29891	19B	A.28	S
hCoV-19/Egypt/ARMY-50/2021	29.34028	good	EPI_ISL_1936226	29881	20D	C.36	GR
hCoV-19/Egypt/ARMY-347/2021	200.6944	bad	EPI_ISL_1936277	29883	19B	A.28	S
hCoV-19/Egypt/ARMY-354/2021	21.00694	good	EPI_ISL_1936284	29880	20I (Alpha, V1)	None	GR
hCoV-19/Egypt/ARMY-355/2021	21.00694	good	EPI_ISL_1936285	29877	20I (Alpha, V1)	None	GR
hCoV-19/Egypt/PHARCO-ARMY-50/2021	100	bad	EPI_ISL_1936310	29793	20A	B.1	O
hCoV-19/Egypt/PHARCO-ARMY-58/2021	100	bad	EPI_ISL_1936318	29793	20A	B.1	O
hCoV-19/Egypt/CPHL-A1/2021			EPI_ISL_3262210	24639	None	None	O
hCoV-19/Egypt/CPHL-A2/2021	14.0625	good	EPI_ISL_3274158	29836	20D	C.36	GR
hCoV-19/Egypt/CPHL-A4/2021	0	good	EPI_ISL_3274160	29829	20D	C.36.3	GR
hCoV-19/Egypt/CPHL-A5/2021	69.44444	mediocre	EPI_ISL_3274161	29836	20D	C.36	GR
hCoV-19/Egypt/CPHL-A6/2021	34.02778	mediocre	EPI_ISL_3274162	29836	20A	B.1.466	GH
hCoV-19/Egypt/CPHL-A8/2020	0.173611	good	EPI_ISL_3274163	29836	20D	C.36	GR
hCoV-19/Egypt/CPHL-A9/2021	11.11111	good	EPI_ISL_3274164	29833	20A	B.1	GH
hCoV-19/Egypt/CPHL-A10/2021	0	good	EPI_ISL_3274165	29830	19B	A.28	S
hCoV-19/Egypt/CUNCI-HGC9I036/2020	1.5625	good	EPI_ISL_857336	29787	19B	A.28	S
hCoV-19/Egypt/NRC1-2/2020	0	good	EPI_ISL_1273103	29864	20A	B.1	GH
hCoV-19/Egypt/CUNCI-HGC10I015/2020	252.25	bad	EPI_ISL_907091	29793	20D	C.36	GR
hCoV-19/Egypt/CPHL-NRC-S15/2021	48.12248	mediocre	EPI_ISL_2313054	29658	20D	C.36.3	GR
hCoV-19/Egypt/CPHL-NRC-S18/2021	63.1142	mediocre	EPI_ISL_2313051	29879	20D	C.36.3	GR
hCoV-19/Egypt/CPHL-NRC-S13/2021	499.4083	bad	EPI_ISL_2313056	29881	20D	C.36	GR
hCoV-19/Egypt/CPHL-NRC-S5/2021	427.6397	bad	EPI_ISL_2313064	28889	20D	C.36	GR
hCoV-19/Egypt/CPHL-NRC-S20/2021	212.5116	bad	EPI_ISL_2313049	29786	20D	C.36.3	GR
hCoV-19/Egypt/MASRI-C4-037/2020	56.42361	mediocre	EPI_ISL_1165080	29808	20D	C.36	GR

hCoV-19/Egypt/NRC-6885/2020	0.694444	good	EPI_ISL_2232332	29901	20D	C.36	GR
hCoV-19/Egypt/NRC-6611/2020	0.173611	good	EPI_ISL_2232333	29903	20D	C.36	GR
hCoV-19/Egypt/NRC-6215/2020	0	good	EPI_ISL_2232334	29902	20D	C.36	GR
hCoV-19/Egypt/NRC-5611/2020	0	good	EPI_ISL_2232314	29891	20D	C.36	GR
hCoV-19/Egypt/NRC-6894/2020	0	good	EPI_ISL_2232331	29902	20D	C.36	GR
hCoV-19/Egypt/NRC-5756/2020	2.454475	good	EPI_ISL_2232252	29902	20B	B.1.1	GR
hCoV-19/Egypt/NRC-6411/2020	62.5	mediocre	EPI_ISL_2232261	29878	20D	C.36	GR
hCoV-19/Egypt/NRC-5852/2020	57.8125	mediocre	EPI_ISL_2232267	29878	20D	C.36	GR
hCoV-19/Egypt/NRC-5836/2020	60.59028	mediocre	EPI_ISL_2232268	29878	20A	B.1	GH
hCoV-19/Egypt/NRC-6957/2020	81.25	mediocre	EPI_ISL_2232269	29865	20A	B.1	GH
hCoV-19/Egypt/NRC-6636/2020	118.9236	bad	EPI_ISL_2232275	29878	20D	C.36	GR
hCoV-19/Egypt/NRC-6377/2020	56.42361	mediocre	EPI_ISL_2232277	29873	20D	C.36	GR
hCoV-19/Egypt/NRC-6099/2020	67.36111	mediocre	EPI_ISL_2232284	29873	20A	B.1	GH
hCoV-19/Egypt/NRC-5982/2020	59.02778	mediocre	EPI_ISL_2232288	29891	20A	B.1	GH
hCoV-19/Egypt/NRC-5873/2020	64.75694	mediocre	EPI_ISL_2232291	29904	20A	B.1.170	GH
hCoV-19/Egypt/NRC-6073/2020	1.5625	good	EPI_ISL_2232299	29862	20A	B.1	GH
hCoV-19/Egypt/NRC-5589/2020	0	good	EPI_ISL_2232300	29871	20B	B.1.1	GR
hCoV-19/Egypt/NRC-6991/2020	21.00694	good	EPI_ISL_2232306	29903	20D	C.36	GR
hCoV-19/Egypt/NRC-6410/2020	0	good	EPI_ISL_2232308	29891	20D	C.36	GR
hCoV-19/Egypt/NRC-6539/2020	21.00694	good	EPI_ISL_2232309	29901	20A	B.1	GH
hCoV-19/Egypt/NRC-5610/2020	0	good	EPI_ISL_2232313	29891	20D	C.36	GR
hCoV-19/Egypt/NRC-5643/2020	0.694444	good	EPI_ISL_2232315	29903	20B	B.1.1	GR
hCoV-19/Egypt/NRC-5540/2020	0	good	EPI_ISL_2232319	29899	20D	C.36	GR
hCoV-19/Egypt/NRC-5853/2020	0	good	EPI_ISL_2232321	29899	20D	C.36	GR
hCoV-19/Egypt/NRC-5464/2020	0	good	EPI_ISL_2232325	29903	20D	C.36	GR
hCoV-19/Egypt/NRC-6888/2020	1.5625	good	EPI_ISL_2232330	29902	20D	C.36	GR
hCoV-19/Egypt/NRC-5456/2020	0.173611	good	EPI_ISL_2232327	29902	20B	B.1.1	GR
hCoV-19/Egypt/NRC-5446/2020	0	good	EPI_ISL_2232328	29902	20D	C.36	GR
hCoV-19/Egypt/NRC-6219/2020	0	good	EPI_ISL_2232335	29903	20D	C.36	GR
hCoV-19/Egypt/NRC-5817/2020	1.5625	good	EPI_ISL_2232341	29878	20A	B.1	GH
hCoV-19/Egypt/NRC-5461/2020	1.5625	good	EPI_ISL_2232343	29903	20B	B.1.1	GR
hCoV-19/Egypt/NRC-5451/2020	0	good	EPI_ISL_2232346	29903	20D	C.36	GR
hCoV-19/Egypt/NRC-6985/2020	2.777778	good	EPI_ISL_2232354	29903	20A	B.1	GH
hCoV-19/Egypt/NRC-6822/2020	0.173611	good	EPI_ISL_2232359	29900	20A	B.1	GH
hCoV-19/Egypt/NRC-6449/2020	0	good	EPI_ISL_2232368	29902	20D	C.36	GR
hCoV-19/Egypt/NRC-6216/2020	0	good	EPI_ISL_2232370	29895	20D	C.36	GR
hCoV-19/Egypt/NRC-5625/2020	1.5625	good	EPI_ISL_2232379	29903	20B	B.1.1	GR
hCoV-19/Egypt/NRC-6157/2020	0	good	EPI_ISL_2232382	29902	20D	C.36	GR
hCoV-19/Egypt/NRC-5885/2020	1.5625	good	EPI_ISL_2232395	29903	20A	B.1.170	GH
hCoV-19/Egypt/NRC-5870/2020	11.11111	good	EPI_ISL_2232398	29868	20A	B.1.517	G
hCoV-19/Egypt/NRC-5463/2020	0	good	EPI_ISL_2232405	29903	20A	B.1.170	GH
hCoV-19/Egypt/NRC-5159/2020	0	good	EPI_ISL_2232407	29898	20D	C.36	GR
hCoV-19/Egypt/MASRI-C4-029/2020	56.25	mediocre	EPI_ISL_1165078	29830	20D	C.36	GR

hCoV-19/Egypt/MASRI-C4-033/2020	677.2569	bad	EPI_ISL_1165079	29805	20D	C.36	GR
hCoV-19/Egypt/MASRI-C4-038/2020			EPI_ISL_1165081	29171	None	C.36	GR
hCoV-19/Egypt/MASRI-C4-023/2020			EPI_ISL_1165083	29779	20A	B.1	GH
hCoV-19/Egypt/MASRI-C4-004/2020	102.2569	bad	EPI_ISL_1165084	24076	None	None	GR
hCoV-19/Egypt/MASRI-C4-012/2020			EPI_ISL_1165085	27911	None	B.1.517	GH
hCoV-19/Egypt/MASRI-C4-014/2020			EPI_ISL_1165086	28153	None	B.1.36.1<br>9	GH
hCoV-19/Egypt/MASRI-C4-018/2020	56.25	mediocre	EPI_ISL_1165087	29603	20A	B.1	GH
hCoV-19/Egypt/MASRI-C5-003/2020			EPI_ISL_1167186	26166	None	B.1.1.36<br>5	GR
hCoV-19/Egypt/MASRI-C5-005/2020			EPI_ISL_1167187	9846	None	None	O
hCoV-19/Egypt/MASRI-C5-007/2020			EPI_ISL_1167188	20625	None	None	GH
hCoV-19/Egypt/MASRI-C5-009/2020	56.25	mediocre	EPI_ISL_1167189	29750	20D	C.1	GR
hCoV-19/Egypt/MASRI-C5-010/2020			EPI_ISL_1167190	16309	None	None	O
hCoV-19/Egypt/MASRI-C5-017/2020			EPI_ISL_1167191	16237	None	None	G
hCoV-19/Egypt/MASRI-C5-020/2020			EPI_ISL_1167192	26574	None	B.1	GH
hCoV-19/Egypt/MASRI-C5-026/2020			EPI_ISL_1167194	27511	None	B.1.426	GH
hCoV-19/Egypt/MASRI-C5-027/2020			EPI_ISL_1167195	27100	None	B.1.1.13<br>3	G
hCoV-19/Egypt/MASRI-C5-039/2020			EPI_ISL_1167196	14923	None	None	GR
hCoV-19/Egypt/MASRI-C5-042/2020			EPI_ISL_1167197	28740	None	C.36	GR
hCoV-19/Egypt/MASRI-011/2020	42.36111	mediocre	EPI_ISL_1097026	29903	20D	C.36	GR
hCoV-19/Egypt/MASRI-014/2020			EPI_ISL_1097028	28153	None	B.1.36.1<br>9	GH
hCoV-19/Egypt/MASRI-018/2020	56.25	mediocre	EPI_ISL_1097029	29603	20A	B.1	GH
hCoV-19/Egypt/MASRI-C5-021/2020	509.0278	bad	EPI_ISL_1167193	29802	20D	C.36	GR
hCoV-19/Egypt/MASRI-012/2020			EPI_ISL_1097027	27911	None	B.1.517	GH
hCoV-19/Egypt/CUNCI-HGC008/2020	1	good	EPI_ISL_468044	29760	20A	B.1	GH
hCoV-19/Egypt/CUNCI-HGC002/2020	49	mediocre	EPI_ISL_468045	29760	20A	B.1	GH
hCoV-19/Egypt/CUNCI-HGC007/2020	0	good	EPI_ISL_468046	29760	20A	B.1	GH
hCoV-19/Egypt/CUNCI-HGC013/2020	0	good	EPI_ISL_468047	29760	20A	B.1.201	G
hCoV-19/Egypt/CUNCI-HGC004/2020	0	good	EPI_ISL_468048	29760	20A	B.1	G
hCoV-19/Egypt/CUNCI-HGC003/2020	0	good	EPI_ISL_468049	29760	20A	B.1	GH
hCoV-19/Egypt/CUNCI-HGC006/2020	1	good	EPI_ISL_468050	29795	20A	B.1	GH
hCoV-19/Egypt/CUNCI-HGC012/2020	1	good	EPI_ISL_468051	29760	20A	B.1	GH
hCoV-19/Egypt/CUNCI-HGC005/2020	0	good	EPI_ISL_468052	29760	20A	B.1	G
hCoV-19/Egypt/CUNCI-HGC009/2020	121	bad	EPI_ISL_468053	29760	20A	B.1	GH
hCoV-19/Egypt/CUNCI-HGC007-2/2020	0	good	EPI_ISL_468054	29801	20A	B.1	GH
hCoV-19/Egypt/CUNCI-HGC010/2020	0.173611	good	EPI_ISL_468055	29797	20A	B.1	GH
hCoV-19/Egypt/CUNCI-HGC015/2020	16	good	EPI_ISL_468056	29760	20A	B.1	G
hCoV-19/Egypt/CUNCI-HGC023/2020	64	mediocre	EPI_ISL_468057	29760	20A	B.1	G
hCoV-19/Egypt/CUNCI-HGC016/2020	225	bad	EPI_ISL_468058	29760	20A	B.1	GH
hCoV-19/Egypt/CUNCI-HGC011/2020	4	good	EPI_ISL_468059	29760	20A	B.1	GH
hCoV-19/Egypt/CUNCI-HGC014/2020	36	mediocre	EPI_ISL_468060	29760	20A	B.1	G
hCoV-19/Egypt/CUNCI-HGC021/2020	256	bad	EPI_ISL_468061	29760	20A	B.1	G
hCoV-19/Egypt/CUNCI-HGC028/2020	1164.507	bad	EPI_ISL_468062	29760	19A	B.1	O

hCoV-19/Egypt/CUNCI-HGC002-2/2020	49	mediocre	EPI_ISL_469275	29760	20A	B.1	GH
hCoV-19/Egypt/CUNCI-HGC5I015/2020	1.5625	good	EPI_ISL_475722	29799	20A	B.1	GH
hCoV-19/Egypt/CUNCI-HGC6I016/2020	0	good	EPI_ISL_475723	29799	20A	B.1.36	GH
hCoV-19/Egypt/CUNCI-HGC6I031/2020	21.00694	good	EPI_ISL_475724	29799	20D	C.36	GR
hCoV-19/Egypt/MASRI-2/2020	0	good	EPI_ISL_475745	29830	20A	B.1	GH
hCoV-19/Egypt/MASRI-3/2020	0	good	EPI_ISL_475746	29816	19B	A	S
hCoV-19/Egypt/MASRI-6/2020	0	good	EPI_ISL_475747	29826	20A	B.1	GH
hCoV-19/Egypt/MASRI-13/2020	0	good	EPI_ISL_475748	29814	20A	B.1	GH
hCoV-19/Egypt/MASRI-14/2020	0	good	EPI_ISL_475749	29816	20A	B.1	GH
hCoV-19/Egypt/MASRI-15/2020	62.5	mediocre	EPI_ISL_475750	29612	20B	B.1.1	GR
hCoV-19/Egypt/MASRI-9/2020	0	good	EPI_ISL_475751	29815	20D	C.36	GR
hCoV-19/Egypt/MASRI-10/2020	0	good	EPI_ISL_475752	29814	20A	B.1.170	GH
hCoV-19/Egypt/MASRI-11/2020	0	good	EPI_ISL_475753	29827	20A	B.1	GH
hCoV-19/Egypt/CUNCI-HGC6I029/2020	0.173611	good	EPI_ISL_477161	29871	20A	B.1	O
hCoV-19/Egypt/CUNCI-HGC6I010/2020	2.777778	good	EPI_ISL_478672	29838	20A	B.1.170	GH
hCoV-19/Egypt/CUNCI-HGC3I02/2020	27.77778	good	EPI_ISL_479686	29846	20A	B.1	O
hCoV-19/Egypt/CUNCI-HGC3I03/2020	0	good	EPI_ISL_479687	29835	20A	B.1	GH
hCoV-19/Egypt/CUNCI-HGC3I06/2020	1	good	EPI_ISL_479688	29873	20A	B.1	GH
hCoV-19/Egypt/CUNCI-HGC3I07/2020	0	good	EPI_ISL_479689	29862	20A	B.1	GH
hCoV-19/Egypt/CUNCI-HGC3I05/2020	0	good	EPI_ISL_479690	29898	20A	B.1	G
hCoV-19/Egypt/CUNCI-HGC3I025/2020	16.69444	good	EPI_ISL_479691	29835	20A	B.1	G
hCoV-19/Egypt/CUNCI-HGC3I09/2020	121	bad	EPI_ISL_479692	29851	20A	B.1	GH
hCoV-19/Egypt/CUNCI-HGC3I012/2020	1	good	EPI_ISL_479693	29843	20A	B.1	GH
hCoV-19/Egypt/CUNCI-HGC3I013/2020	0	good	EPI_ISL_479694	29872	20A	B.1.201	G
hCoV-19/Egypt/CUNCI-HGC3I023/2020	64	mediocre	EPI_ISL_479695	29868	20A	B.1	G
hCoV-19/Egypt/CUNCI-HGC3I014/2020	25	good	EPI_ISL_479696	29835	20A	B.1	G
hCoV-19/Egypt/CUNCI-HGC3I011/2020	4	good	EPI_ISL_479697	29857	20A	B.1	GH
hCoV-19/Egypt/CUNCI-HGC3I08/2020	1	good	EPI_ISL_479698	29874	20A	B.1	GH
hCoV-19/Egypt/CUNCI-HGC4I003/2020	0	good	EPI_ISL_479699	29834	20B	B.1.1	GR
hCoV-19/Egypt/CUNCI-HGC4I004/2020	0	good	EPI_ISL_479700	29818	20B	B.1.1	GR
hCoV-19/Egypt/CUNCI-HGC4I029/2020	0	good	EPI_ISL_479701	29860	20D	C.36	GR
hCoV-19/Egypt/CUNCI-HGC4I026/2020	4.340278	good	EPI_ISL_479702	29792	20A	B.1	O
hCoV-19/Egypt/CUNCI-HGC4I030/2020	0	good	EPI_ISL_479703	29837	20A	B.1	GH
hCoV-19/Egypt/CUNCI-HGC4I033/2020	51.77778	mediocre	EPI_ISL_479704	29833	20A	B.1	O
hCoV-19/Egypt/CUNCI-HGC4I022/2020	0.694444	good	EPI_ISL_479705	29835	20B	B.1.1	GR
hCoV-19/Egypt/CUNCI-HGC4I025/2020	1.694444	good	EPI_ISL_479706	29887	20A	B.1	GH
hCoV-19/Egypt/CUNCI-HGC4I031/2020	11.11111	good	EPI_ISL_479707	29836	20D	C.36	GR
hCoV-19/Egypt/CUNCI-HGC4I034/2020	0	good	EPI_ISL_479708	29837	20A	B.1	GH
hCoV-19/Egypt/CUNCI-HGC4I032/2020	0	good	EPI_ISL_479709	29810	20B	B.1.1	GR
hCoV-19/Egypt/CUNCI-HGC5I001/2020	144	bad	EPI_ISL_479710	29843	20B	B.1.1	GR
hCoV-19/Egypt/CUNCI-HGC5I003/2020	9	good	EPI_ISL_479711	29860	20A	B.1	G
hCoV-19/Egypt/CUNCI-HGC5I011/2020	64	mediocre	EPI_ISL_479712	29835	20B	B.1.1	GR
hCoV-19/Egypt/CUNCI-HGC5I012/2020	1	good	EPI_ISL_479713	29839	20B	B.1.1	GR

hCoV-19/Egypt/CUNCI-HGC5I014/2020	0	good	EPI_ISL_479714	29885	20A	B.1	GH
hCoV-19/Egypt/CUNCI-HGC5I022/2020	0	good	EPI_ISL_479715	29898	20B	B.1.1	GR
hCoV-19/Egypt/CUNCI-HGC5I033/2020	144	bad	EPI_ISL_479717	29870	20A	B.1	G
hCoV-19/Egypt/CUNCI-HGC6I003/2020	1	good	EPI_ISL_479718	29836	20A	B.1	O
hCoV-19/Egypt/CUNCI-HGC6I002/2020	0	good	EPI_ISL_479719	29851	20D	C.36	GR
hCoV-19/Egypt/CUNCI-HGC5I034/2020	1	good	EPI_ISL_479720	29834	20A	B.1	O
hCoV-19/Egypt/CUNCI-HGC5I030/2020	81	mediocre	EPI_ISL_479721	29895	20A	B.1	O
hCoV-19/Egypt/CUNCI-HGC6I007/2020	100	bad	EPI_ISL_479722	29871	20A	B.1	G
hCoV-19/Egypt/CUNCI-HGC6I033/2020	22.00694	good	EPI_ISL_479723	29847	20D	C.17	O
hCoV-19/Egypt/CUNCI-HGC6I026/2020	0	good	EPI_ISL_479724	29834	20D	C.36	GR
hCoV-19/Egypt/CUNCI-HGC6I015/2020	0	good	EPI_ISL_479725	29890	20B	B.1.1	GR
hCoV-19/Egypt/CUNCI-HGC6I009/2020	0	good	EPI_ISL_479726	29857	20D	C.36	GR
hCoV-19/Egypt/CUNCI-HGC6I011/2020	5.340278	good	EPI_ISL_479727	29835	19B	A	S
hCoV-19/Egypt/CUNCI-HGC4I015/2020	0	good	EPI_ISL_479728	29799	20B	B.1.1	GR
hCoV-19/Egypt/CUNCI-HGC5I010/2020	83.77778	mediocre	EPI_ISL_479730	29799	20B	B.1.1	O
hCoV-19/Egypt/CUNCI-HGC5I016/2020	0	good	EPI_ISL_479731	29799	20A	B.1	GH
hCoV-19/Egypt/CUNCI-HGC6I024/2020	0	good	EPI_ISL_479732	29768	20B	B.1.1	GR
hCoV-19/Egypt/CUNCI-HGC6I032/2020	0	good	EPI_ISL_479733	29799	20D	C.36	GR
hCoV-19/Egypt/CUNCI-HGC5I029/2020	16	good	EPI_ISL_479734	29799	20A	B.1	GH
hCoV-19/Egypt/CUNCI-HGC5I026/2020	37.5625	mediocre	EPI_ISL_479735	29799	20A	B.1	O
hCoV-19/Egypt/MASRI-005/2020	0	good	EPI_ISL_482759	29826	20A	B.1.195	G
hCoV-19/Egypt/MASRI-007/2020	0	good	EPI_ISL_482760	29832	20A	B.1.195	G
hCoV-19/Egypt/MASRI-008/2020	0	good	EPI_ISL_482761	29814	20A	B.1.195	G
hCoV-19/Egypt/MASRI-011/2020	0	good	EPI_ISL_482762	29833	20A	B.1.195	G
hCoV-19/Egypt/MASRI-012/2020	0	good	EPI_ISL_482763	29842	20A	B.1.195	G
hCoV-19/Egypt/MASRI-013/2020	0	good	EPI_ISL_482764	29831	20A	B.1.195	G
hCoV-19/Egypt/MASRI-014/2020	0.173611	good	EPI_ISL_482765	29825	20A	B.1.466	GH
hCoV-19/Egypt/MASRI-015/2020	0	good	EPI_ISL_482766	29815	20A	B.1	GH
hCoV-19/Egypt/MASRI-016/2020	0	good	EPI_ISL_482767	29834	20A	B.1	GH
hCoV-19/Egypt/MASRI-022/2020	0	good	EPI_ISL_482768	29823	20A	B.1	GH
hCoV-19/Egypt/MASRI-023/2020	0	good	EPI_ISL_482769	29818	20A	B.1	GH
hCoV-19/Egypt/MASRI-024/2020	0	good	EPI_ISL_482770	29824	20A	B.1	GH
hCoV-19/Egypt/MASRI-025/2020	62.5	mediocre	EPI_ISL_482771	29819	20A	B.1	GH
hCoV-19/Egypt/MASRI-026/2020	0	good	EPI_ISL_482772	29827	20A	B.1	GH
hCoV-19/Egypt/MASRI-027/2020	0	good	EPI_ISL_482773	29780	20B	B.1.1	GR
hCoV-19/Egypt/MASRI-029/2020	0	good	EPI_ISL_482774	29828	20A	B.1	GH
hCoV-19/Egypt/MASRI-030/2020	434.9028	bad	EPI_ISL_482775	29814	20B	B.1.1	GR
hCoV-19/Egypt/MASRI-009/2020	0	good	EPI_ISL_483035	29834	19B	A	S
hCoV-19/Egypt/MASRI-018/2020	6.25	good	EPI_ISL_483036	29816	19B	A	S
hCoV-19/Egypt/MASRI-020/2020	0.173611	good	EPI_ISL_483037	29824	20A	B.1	GH
hCoV-19/Egypt/MASRI-028/2020	0	good	EPI_ISL_483038	29828	20C	B.1	GH
hCoV-19/Egypt/Army-MCL001/2020	1.5625	good	EPI_ISL_510526	29857	20A	B.1	GH
hCoV-19/Egypt/HCoV2-Egy-002/2020	2.777778	good	EPI_ISL_510532	29859	20A	B.1	GH

hCoV-19/Egypt/CUNCI-7I028/2020	0	good	EPI_ISL_524426	29793	20A	B.1	GH
hCoV-19/Egypt/CUNCI-7I026/2020	0	good	EPI_ISL_524427	29793	20A	B.1	GH
hCoV-19/Egypt/EGY-020/2020	1.5625	good	EPI_ISL_526975	29845	20A	B.1	GH
hCoV-19/Egypt/EGY-021/2020	1.5625	good	EPI_ISL_526976	29845	20A	B.1	GH
hCoV-19/Egypt/EGY-022/2020	1.5625	good	EPI_ISL_526977	29845	20A	B.1	GH
hCoV-19/Egypt/EGY-023/2020	1.5625	good	EPI_ISL_526978	29845	20A	B.1	GH
hCoV-19/Egypt/EGY-024/2020	1.5625	good	EPI_ISL_526979	29845	20A	B.1	GH
hCoV-19/Egypt/EGY-025/2020	1.5625	good	EPI_ISL_526980	29845	20A	B.1	GH
hCoV-19/Egypt/EGY-026/2020	1.5625	good	EPI_ISL_526981	29845	20A	B.1	GH
hCoV-19/Egypt/EGY-027/2020	1.5625	good	EPI_ISL_526982	29845	20A	B.1	GH
hCoV-19/Egypt/EGY-028/2020	1.5625	good	EPI_ISL_526983	29845	20A	B.1	GH
hCoV-19/Egypt/EGY-029/2020	1.5625	good	EPI_ISL_526984	29845	20A	B.1	GH
hCoV-19/Egypt/EGY-030/2020	1.5625	good	EPI_ISL_526985	29845	20A	B.1	GH
hCoV-19/Egypt/EGY-S031/2020	1.5625	good	EPI_ISL_526986	29845	20A	B.1	GH
hCoV-19/Egypt/EGY-S032/2020	1.5625	good	EPI_ISL_526987	29845	20A	B.1	GH
hCoV-19/Egypt/EGY-S034/2020	1.5625	good	EPI_ISL_526989	29845	20A	B.1	GH
hCoV-19/Egypt/EGY-S035/2020	1.5625	good	EPI_ISL_526990	29845	20A	B.1	GH
hCoV-19/Egypt/EGY-S036/2020	1.5625	good	EPI_ISL_526991	29845	20A	B.1	GH
hCoV-19/Egypt/EGY-S037/2020	1.5625	good	EPI_ISL_526992	29845	20A	B.1	GH
hCoV-19/Egypt/EGY-S038/2020	1.5625	good	EPI_ISL_526993	29845	20A	B.1	GH
hCoV-19/Egypt/EGY-S039/2020	1.5625	good	EPI_ISL_526994	29845	20A	B.1	GH
hCoV-19/Egypt/EGY-S040/2020	1.5625	good	EPI_ISL_526995	29845	20A	B.1	GH
hCoV-19/Egypt/EGY-S030/2020	1.5625	good	EPI_ISL_526996	29845	20A	B.1	GH
hCoV-19/Egypt/EGY-041/2020	1.5625	good	EPI_ISL_526997	29845	20A	B.1	GH
hCoV-19/Egypt/EGY-042/2020	1.5625	good	EPI_ISL_526998	29845	20A	B.1	GH
hCoV-19/Egypt/EGY-044/2020	1.5625	good	EPI_ISL_526999	29845	20A	B.1	GH
hCoV-19/Egypt/EGY-043/2020	1.5625	good	EPI_ISL_527000	29845	20A	B.1	GH
hCoV-19/Egypt/EGY-045/2020	1.5625	good	EPI_ISL_527001	29845	20A	B.1	GH
hCoV-19/Egypt/EGY-046/2020	1.5625	good	EPI_ISL_527002	29845	20A	B.1	GH
hCoV-19/Egypt/EGY-047/2020	1.5625	good	EPI_ISL_527003	29845	20A	B.1	GH
hCoV-19/Egypt/EGY-048/2020	1.5625	good	EPI_ISL_527004	29845	20A	B.1	GH
hCoV-19/Egypt/EGY-049/2020	1.5625	good	EPI_ISL_527005	29845	20A	B.1	GH
hCoV-19/Egypt/EGY-050/2020	1.5625	good	EPI_ISL_527006	29845	20A	B.1	GH
hCoV-19/Egypt/EGY-040/2020	1.5625	good	EPI_ISL_527007	29845	20A	B.1	GH
hCoV-19/Egypt/C-CU001a-S1/2020	0	good	EPI_ISL_529031	29787	20A	B.1	GH
hCoV-19/Egypt/C-CU002b-S3/2020	0	good	EPI_ISL_529032	29792	20D	C.36	GR
hCoV-19/Egypt/CUNCI-HGC7I01/2020	0	good	EPI_ISL_529141	29793	20A	B.1	GH
hCoV-19/Egypt/CUNCI-HGC7I02/2020	2.777778	good	EPI_ISL_529142	29793	19B	A	S
hCoV-19/Egypt/CUNCI-HGC7I029/2020	0	good	EPI_ISL_529143	29793	20A	B.1	GH
hCoV-19/Egypt/CUNCI-HGC7I030/2020	0	good	EPI_ISL_529144	29793	20A	B.1	GH
hCoV-19/Egypt/CUNCI-HGC7I07/2020	1	good	EPI_ISL_529145	29793	20A	B.1	GH
hCoV-19/Egypt/CUNCI-HGC7I010/2020	1	good	EPI_ISL_576371	29808	20B	B.1.1	GR
hCoV-19/Egypt/CUNCI-HGC7I023/2020	3.777778	good	EPI_ISL_576372	29808	20D	C.36	GR

hCoV-19/Egypt/CUNCI-HGC7I025/2020	0	good	EPI_ISL_576373	29808	20A	B.1	GH
hCoV-19/Egypt/C-CEIRS-19MOH/2020	4	good	EPI_ISL_605780	29855	20A	B.1	GH
hCoV-19/Egypt/C-CEIRS-8MOH/2020	52.8242	mediocre	EPI_ISL_605782	29808	20A	B.1	G
hCoV-19/Egypt/C-CEIRS-2MOH/2020	69.19453	mediocre	EPI_ISL_605781	29807	20B	B.1.1	GR
hCoV-19/Egypt/CPHL-NRC-18/2020	0.012346	good	EPI_ISL_794594	29777	20A	B.1.535	G
hCoV-19/Egypt/CPHL-NRC-17/2020	0.516269	good	EPI_ISL_794595	29865	20A	B.1.535	G
hCoV-19/Egypt/CPHL-NRC-16/2020	0.364458	good	EPI_ISL_794596	29790	20A	B.1	GH
hCoV-19/Egypt/CPHL-NRC-14/2020	1.226351	good	EPI_ISL_794598	29821	20A	B.1	GH
hCoV-19/Egypt/CPHL-NRC-10/2020	24.41092	good	EPI_ISL_794599	29815	20A	B.1	GH
hCoV-19/Egypt/CPHL-NRC-3/2020	4.286433	good	EPI_ISL_794600	29865	20A	B.1	GH
hCoV-19/Egypt/CPHL-NRC-1/2020	0.051043	good	EPI_ISL_794601	29864	20A	B.1	GH
hCoV-19/Egypt/CPHL-NRC-23/2020	0.898985	good	EPI_ISL_794602	29865	20A	B.1	GH
hCoV-19/Egypt/CPHL-NRC-22/2020	0	good	EPI_ISL_794603	29864	20A	B.1	GH
hCoV-19/Egypt/CPHL-NRC-21/2020	0	good	EPI_ISL_794604	29843	20A	B.1	GH
hCoV-19/Egypt/CPHL-NRC-9/2020	1212.845	bad	EPI_ISL_794605	29622	20A	None	G
hCoV-19/Egypt/CPHL-NRC-6/2020	13.4173	good	EPI_ISL_794606	29864	20A	B.1	G
hCoV-19/Egypt/EGY-Cairo/2020	1.5625	good	EPI_ISL_794634	29845	20A	B.1	GH
hCoV-19/Egypt/CCHE57357-A-03/2020	0	good	EPI_ISL_812785	29903	19A	B	O
hCoV-19/Egypt/CCHE57357-A-06/2020	0	good	EPI_ISL_812787	29903	20D	C.36	GR
hCoV-19/Egypt/CCHE57357-A-09/2020	0.694444	good	EPI_ISL_812790	29903	20A	B.1	O
hCoV-19/Egypt/CCHE57357-A-12/2020	0	good	EPI_ISL_812792	29903	20A	B.1	GH
hCoV-19/Egypt/CCHE57357-A-14/2020	0	good	EPI_ISL_812794	29903	20A	B.1	GH
hCoV-19/Egypt/CCHE57357-A-19/2020	0	good	EPI_ISL_812797	29903	20D	C.36	GR
hCoV-19/Egypt/CCHE57357-A-22/2020	8.506944	good	EPI_ISL_812798	29903	19A	B.1	O
hCoV-19/Egypt/CCHE57357-A-23/2020	0	good	EPI_ISL_812799	29903	20D	C.36	GR
hCoV-19/Egypt/CCHE57357-A-24/2020	50.17361	mediocre	EPI_ISL_812800	29903	20B	B.1.1	O
hCoV-19/Egypt/CCHE57357-A-25/2020	0.694444	good	EPI_ISL_812801	29903	20A	B.1	G
hCoV-19/Egypt/CCHE57357-A-26/2020	0	good	EPI_ISL_812802	29903	19A	B	L
hCoV-19/Egypt/CCHE57357-A-28/2020	0	good	EPI_ISL_812804	29903	19A	B	L
hCoV-19/Egypt/CCHE57357-A-29/2020	1.5625	good	EPI_ISL_812805	29903	19A	B.1	L
hCoV-19/Egypt/CCHE57357-A-30/2020	11.11111	good	EPI_ISL_812806	29903	20A	B.1	G
hCoV-19/Egypt/CCHE57357-A-31/2020	0.173611	good	EPI_ISL_812807	29903	20D	C.36	GR
hCoV-19/Egypt/CCHE57357-A-34/2020	14.0625	good	EPI_ISL_812808	29903	20D	C.36	GR
hCoV-19/Egypt/CCHE57357-A-35/2020	0.173611	good	EPI_ISL_812809	29903	20D	B.1.1.1	GR
hCoV-19/Egypt/CCHE57357-A-36/2020	8.506944	good	EPI_ISL_812810	29903	19A	B	O
hCoV-19/Egypt/CCHE57357-A-38/2020	0	good	EPI_ISL_812811	29903	19A	B	L
hCoV-19/Egypt/CCHE57357-A-39/2020	14.0625	good	EPI_ISL_812812	29903	19A	B	O
hCoV-19/Egypt/CCHE57357-A-40/2020	0	good	EPI_ISL_812813	29903	19A	B	L
hCoV-19/Egypt/CCHE57357-A-41/2020	0.694444	good	EPI_ISL_812814	29903	20B	B.1.1	GR
hCoV-19/Egypt/CCHE57357-A-42/2020	44.44444	mediocre	EPI_ISL_812815	29903	20B	B.1.1	GR
hCoV-19/Egypt/CCHE57357-A-43/2020	2.777778	good	EPI_ISL_812816	29903	20D	C.36	GR
hCoV-19/Egypt/CCHE57357-A-44/2020	2.777778	good	EPI_ISL_812817	29903	20D	C.36	GR
hCoV-19/Egypt/CCHE57357-A-46/2020	4.340278	good	EPI_ISL_812819	29903	20A	B.1	GH

hCoV-19/Egypt/CCHE57357-A-47/2020	34.02778	mediocre	EPI_ISL_812820	29903	19A	B.1	O
hCoV-19/Egypt/CCHE57357-A-48/2020	62.67361	mediocre	EPI_ISL_812821	29903	19B	A.28	S
hCoV-19/Egypt/CCHE57357-A-49/2020	0	good	EPI_ISL_812822	29903	20D	C.36	GR
hCoV-19/Egypt/CCHE57357-A-50/2020	17.36111	good	EPI_ISL_812823	29903	20A	B.1	G
hCoV-19/Egypt/CCHE57357-A-51/2020	76.5625	mediocre	EPI_ISL_812824	29903	20D	C.17	O
hCoV-19/Egypt/CCHE57357-A-52/2020	39.0625	mediocre	EPI_ISL_812825	29903	20B	C.17	O
hCoV-19/Egypt/CCHE57357-A-53/2020	0	good	EPI_ISL_812826	29903	20A	B.1	GH
hCoV-19/Egypt/CCHE57357-A-56/2020	84.02778	mediocre	EPI_ISL_812829	29903	19B	B	O
hCoV-19/Egypt/CCHE57357-A-57/2020	44.44444	mediocre	EPI_ISL_812830	29903	20B	B.1.1	O
hCoV-19/Egypt/CCHE57357-A-58/2020	0	good	EPI_ISL_812831	29903	19A	B	L
hCoV-19/Egypt/CCHE57357-A-59/2020	116.8403	bad	EPI_ISL_812832	29903	20A	B.1.544	GH
hCoV-19/Egypt/CCHE57357-A-60/2020	0	good	EPI_ISL_812833	29903	19A	B	L
hCoV-19/Egypt/CCHE57357-A-62/2020	11.11111	good	EPI_ISL_812834	29903	20D	C.36	GR
hCoV-19/Egypt/CCHE57357-A-66/2020	0.694444	good	EPI_ISL_812836	29903	19A	B	L
hCoV-19/Egypt/CCHE57357-A-67/2020	17.36111	good	EPI_ISL_812837	29903	20B	B.1.1	O
hCoV-19/Egypt/CCHE57357-A-68/2020	0.173611	good	EPI_ISL_812838	29903	19A	B.1	O
hCoV-19/Egypt/CCHE57357-A-69/2020	0	good	EPI_ISL_812839	29903	19A	B	L
hCoV-19/Egypt/CCHE57357-A-70/2020	0	good	EPI_ISL_812840	29903	19A	B	O
hCoV-19/Egypt/CCHE57357-A-71/2020	0	good	EPI_ISL_812841	29903	19A	B	L
hCoV-19/Egypt/CCHE57357-A-72/2020	29.34028	good	EPI_ISL_812842	29903	20B	B.1.1	O
hCoV-19/Egypt/CCHE57357-A-75/2020	1.5625	good	EPI_ISL_812843	29903	19A	A	O
hCoV-19/Egypt/CCHE57357-A-80/2020	11.11111	good	EPI_ISL_812844	29903	19A	B	O
hCoV-19/Egypt/CCHE57357-A-88/2020	0	good	EPI_ISL_812845	29903	19A	B	L
hCoV-19/Egypt/CCHE57357-A-89/2020	50.17361	mediocre	EPI_ISL_812846	29903	20A	B.1	O
hCoV-19/Egypt/CCHE57357-A-91/2020	166.8403	bad	EPI_ISL_812848	29903	20D	B.1	G
hCoV-19/Egypt/CCHE57357-P-01/2020	0.694444	good	EPI_ISL_812850	29903	20A	B.1	O
hCoV-19/Egypt/CCHE57357-P-03/2020	0	good	EPI_ISL_812851	29903	20A	B.1	GH
hCoV-19/Egypt/CCHE57357-P-14/2020	14.0625	good	EPI_ISL_812853	29903	20A	B	O
hCoV-19/Egypt/CCHE57357-P-16/2020	1.5625	good	EPI_ISL_812854	29903	20A	B.1	GH
hCoV-19/Egypt/CCHE57357-P-24/2020	0	good	EPI_ISL_812858	29903	20B	B.1.1	GR
hCoV-19/Egypt/CCHE57357-P-26/2020	8.506944	good	EPI_ISL_812860	29903	19A	B.1.1	L
hCoV-19/Egypt/CCHE57357-P-27/2020	0	good	EPI_ISL_812861	29903	19A	B	L
hCoV-19/Egypt/CCHE57357-P-32/2020	14.0625	good	EPI_ISL_812863	29903	20D	B.1.1.1	GR
hCoV-19/Egypt/CCHE57357-P-33/2020	39.0625	mediocre	EPI_ISL_812864	29903	20D	C.36	GR
hCoV-19/Egypt/CCHE57357-P-34/2020	0	good	EPI_ISL_812865	29903	19A	B.1	O
hCoV-19/Egypt/CCHE57357-P-36/2020	69.44444	mediocre	EPI_ISL_812866	29903	20B	B.1.1	O
hCoV-19/Egypt/CCHE57357-P-39/2020	8.506944	good	EPI_ISL_812869	29903	20D	B.1.1.1	GR
hCoV-19/Egypt/CCHE57357-P-41/2020	0	good	EPI_ISL_812870	29903	19A	B	L
hCoV-19/Egypt/CCHE57357-P-45/2020	25	good	EPI_ISL_812872	29903	20A	B.1	O
hCoV-19/Egypt/CUNCI-HGC8I025/2020	33.36111	mediocre	EPI_ISL_857315	29793	20A	B.1.36	GH
hCoV-19/Egypt/CUNCI-HGC8I031/2020	57.25	mediocre	EPI_ISL_857316	29793	20D	C.36	GR
hCoV-19/Egypt/CUNCI-HGC8I032/2020	7.25	good	EPI_ISL_857317	29793	20D	C.36	GR
hCoV-19/Egypt/CUNCI-HGC8I035/2020	6.25	good	EPI_ISL_857318	29793	20D	C.36	GR

hCoV-19/Egypt/CUNCI-HGC8I034/2020	34.02778	mediocre	EPI_ISL_857319	29793	20D	C.36	GR
hCoV-19/Egypt/CUNCI-HGC8I036/2020	6.25	good	EPI_ISL_857320	29793	20D	C.36	GR
hCoV-19/Egypt/CUNCI-HGC8I037/2020	98.36111	mediocre	EPI_ISL_857321	29793	20D	C.36	GR
hCoV-19/Egypt/CUNCI-HGC9I004/2020	17.36111	good	EPI_ISL_857324	29793	20D	C.36	GR
hCoV-19/Egypt/CUNCI-HGC9I007/2020	36.11111	mediocre	EPI_ISL_857325	29793	20A	B.1.170	GH
hCoV-19/Egypt/CUNCI-HGC9I010/2020	49.69444	mediocre	EPI_ISL_857326	29793	20D	B.1.1.1	GR
hCoV-19/Egypt/CUNCI-HGC9I011/2020	529	bad	EPI_ISL_857327	29793	20A	B.1	G
hCoV-19/Egypt/CUNCI-HGC9I012/2020	44.44444	mediocre	EPI_ISL_857329	29793	20D	C.36	GR
hCoV-19/Egypt/CUNCI-HGC9I023/2020	18.0625	good	EPI_ISL_857330	29793	20A	B.1.398	G
hCoV-19/Egypt/CUNCI-HGC9I035/2020	4.340278	good	EPI_ISL_857331	29793	20D	C.36	GR
hCoV-19/Egypt/CUNCI-HGC9I022/2020	189.0625	bad	EPI_ISL_857332	29793	19B	A	O
hCoV-19/Egypt/CUNCI-HGC9I029/2020	11.11111	good	EPI_ISL_857333	29793	20D	C.36	GR
hCoV-19/Egypt/CUNCI-HGC9I042/2020	1226.563	bad	EPI_ISL_857335	29793	20A	B.1	G
hCoV-19/Egypt/CUNCI-HGC9I037/2020	23.0625	good	EPI_ISL_857337	29793	20A	B.1.466	GH
hCoV-19/Egypt/CUNCI-HGC9I033/2020	50.17361	mediocre	EPI_ISL_857340	29793	20D	C.36	GR
hCoV-19/Egypt/CUNCI-HGC9I016/2020	69.44444	mediocre	EPI_ISL_857342	29793	20B	B.1.1	GR
hCoV-19/Egypt/CUNCI-HGC9I034/2020	1.173611	good	EPI_ISL_857343	29790	20D	C.36	GR
hCoV-19/Egypt/CUNCI-HGC9I027/2020	0.173611	good	EPI_ISL_857345	29793	20D	C.36	GR
hCoV-19/Egypt/CUNCI-HGC9I043/2020	17.36111	good	EPI_ISL_857346	29793	20B	B.1.1	GR
hCoV-19/Egypt/CUNCI-HGC9I038/2020	126.5625	bad	EPI_ISL_857347	29793	20D	C.36	GR
hCoV-19/Egypt/CUNCI-HGC9I030/2020	277.7778	bad	EPI_ISL_862783	29792	20D	C.36	GR
hCoV-19/Egypt/CUNCI-HGC9I025/2020	306.25	bad	EPI_ISL_862784	29792	20D	C.36	GR
hCoV-19/Egypt/CUNCI-HGC10I024/2020	264.0625	bad	EPI_ISL_862785	29792	20D	C.36	GR
hCoV-19/Egypt/CUNCI-HGC11I004/2021	53.44444	mediocre	EPI_ISL_862786	29793	20D	C.36	GR
hCoV-19/Egypt/CUNCI-HGC11I005/2021	9.506944	good	EPI_ISL_862787	29793	20A	B.1.170	GH
hCoV-19/Egypt/CUNCI-HGC11I006/2021	17.36111	good	EPI_ISL_862788	29793	20A	B.1.466	GH
hCoV-19/Egypt/CUNCI-HGC11I008/2021	48.0625	mediocre	EPI_ISL_862789	29793	20A	B.1.177	G
hCoV-19/Egypt/CUNCI-HGC11I009/2021	98.02778	mediocre	EPI_ISL_862790	29793	20D	C.36	GR
hCoV-19/Egypt/CUNCI-HGC11I010/2021	55.0625	mediocre	EPI_ISL_862791	29793	20D	C.36	GR
hCoV-19/Egypt/CUNCI-HGC11I013/2021	100.8403	bad	EPI_ISL_862792	29793	20A	B.1.398	G
hCoV-19/Egypt/CUNCI-HGC11I014/2021	70.02778	mediocre	EPI_ISL_862793	29793	20A	B.1.398	G
hCoV-19/Egypt/CUNCI-HGC11I016/2021	1089	bad	EPI_ISL_862794	29793	20A	B.1	O
hCoV-19/Egypt/CUNCI-HGC11I022/2021	1	good	EPI_ISL_862795	29793	20D	C.36	GR
hCoV-19/Egypt/CUNCI-HGC11I026/2021	83.02778	mediocre	EPI_ISL_862796	29792	20A	B.1.398	G
hCoV-19/Egypt/CUNCI-HGC11I027/2021	20.11111	good	EPI_ISL_862797	29793	20A	B.1.466	GH
hCoV-19/Egypt/CUNCI-HGC11I028/2021	425.8403	bad	EPI_ISL_862798	29793	20D	C.36	GR
hCoV-19/Egypt/CUNCI-HGC11I029/2021	33.34028	mediocre	EPI_ISL_862799	29793	20A	B.1.466	GH
hCoV-19/Egypt/CUNCI-HGC11I031/2021	501.3611	bad	EPI_ISL_862800	29793	20D	C.36	GR
hCoV-19/Egypt/CUNCI-HGC11I032/2021	169	bad	EPI_ISL_862801	29793	20A	B.1.170	GH
hCoV-19/Egypt/CUNCI-HGC11I033/2021	94.44444	mediocre	EPI_ISL_862802	29793	20D	C.36	GR
hCoV-19/Egypt/CUNCI-HGC11I034/2021	9.506944	good	EPI_ISL_862803	29793	20D	C.36	GR
hCoV-19/Egypt/CUNCI-HGC11I035/2021	25.00694	good	EPI_ISL_862804	29793	20A	B.1.170	GH
hCoV-19/Egypt/CUNCI-HGC11I036/2021	45.44444	mediocre	EPI_ISL_862805	29793	20A	B.1.466	GH

hCoV-19/Egypt/CUNCI-HGC11I040/2021	87.67361	mediocre	EPI_ISL_862806	29793	20A	B.1.441	G
hCoV-19/Egypt/CUNCI-HGC11I041/2021	15.0625	good	EPI_ISL_862807	29793	20B	B.1.1.35 3	GR
hCoV-19/Egypt/CUNCI-HGC11I042/2021	45.44444	mediocre	EPI_ISL_862808	29793	20D	C.36	GR
hCoV-19/Egypt/CUNCI-HGC11I047/2021	54.17361	mediocre	EPI_ISL_862809	29793	20D	C.36	GR
hCoV-19/Egypt/CUNCI-HGC11I048/2021	229.3403	bad	EPI_ISL_862810	29793	20A	B.1	G
hCoV-19/Egypt/CUNCI-HGC11I049/2021	60.44444	mediocre	EPI_ISL_862811	29793	20D	B.1	G
hCoV-19/Egypt/CUNCI-HGC11I052/2021	69.44444	mediocre	EPI_ISL_862812	29793	20D	C.36	GR
hCoV-19/Egypt/CUNCI-HGC11I053/2021	1.173611	good	EPI_ISL_862813	29793	20D	C.36	GR
hCoV-19/Egypt/CUNCI-HGC12I001/2021	49.17361	mediocre	EPI_ISL_890195	29793	20A	B.1.170	GH
hCoV-19/Egypt/CUNCI-HGC12I003/2021	26	good	EPI_ISL_890196	29793	20A	B.1.398	G
hCoV-19/Egypt/CUNCI-HGC12I004/2021	289	bad	EPI_ISL_890197	29793	20A	B.1	G
hCoV-19/Egypt/CUNCI-HGC12I005/2021	100	bad	EPI_ISL_890198	29793	20A	B.1	O
hCoV-19/Egypt/CUNCI-HGC12I007/2021	17.36111	good	EPI_ISL_890199	29793	20D	C.36	GR
hCoV-19/Egypt/CUNCI-HGC12I008/2021	25.69444	good	EPI_ISL_890200	29793	20D	C.36	GR
hCoV-19/Egypt/CUNCI-HGC12I009/2021	447.25	bad	EPI_ISL_890201	29793	20D	C.36	GR
hCoV-19/Egypt/CUNCI-HGC12I010/2021	1024.174	bad	EPI_ISL_890202	29793	20B	C.17	GR
hCoV-19/Egypt/CUNCI-HGC12I011/2021	0.694444	good	EPI_ISL_890203	29793	20D	C.36	GR
hCoV-19/Egypt/CUNCI-HGC12I012/2021	733.3403	bad	EPI_ISL_890204	29793	20D	C.17	GR
hCoV-19/Egypt/CUNCI-HGC12I013/2021	1024.174	bad	EPI_ISL_890205	29793	20B	C.17	GR
hCoV-19/Egypt/CUNCI-HGC12I014/2021	6.25	good	EPI_ISL_890206	29793	20D	C.36	GR
hCoV-19/Egypt/CUNCI-HGC12I016/2021	6.25	good	EPI_ISL_890207	29793	20D	C.36	GR
hCoV-19/Egypt/CUNCI-HGC12I021/2021	69.44444	mediocre	EPI_ISL_890208	29793	20D	C.36	GR
hCoV-19/Egypt/CUNCI-HGC12I022/2021	11.11111	good	EPI_ISL_890209	29793	20D	C.36	GR
hCoV-19/Egypt/CUNCI-HGC12I023/2021	0	good	EPI_ISL_890210	29793	20D	C.36	GR
hCoV-19/Egypt/CUNCI-HGC12I026/2021	169	bad	EPI_ISL_890211	29793	20D	C.36	GR
hCoV-19/Egypt/CUNCI-HGC12I029/2021	101.5625	bad	EPI_ISL_890212	29793	20D	C.36	GR
hCoV-19/Egypt/CUNCI-HGC12I030/2021	1225	bad	EPI_ISL_890213	29793	20A	B.1.1	G
hCoV-19/Egypt/CUNCI-HGC12I031/2021	40.0625	mediocre	EPI_ISL_890214	29793	20D	C.36	GR
hCoV-19/Egypt/CUNCI-HGC12I032/2021	484.1736	bad	EPI_ISL_890215	29793	20B	C.17	GR
hCoV-19/Egypt/CUNCI-HGC12I033/2021	676	bad	EPI_ISL_890216	29793	20A	B.1	G
hCoV-19/Egypt/CUNCI-HGC12I034/2021	1764.694	bad	EPI_ISL_890217	29793	20B	C.36	GR
hCoV-19/Egypt/CUNCI-HGC12I035/2021	1156	bad	EPI_ISL_890218	29793	20A	B.1.398	G
hCoV-19/Egypt/CUNCI-HGC12I036/2021	841	bad	EPI_ISL_890219	29793	20A	B.1.170	G
hCoV-19/Egypt/CUNCI-HGC12I037/2021	41	mediocre	EPI_ISL_890220	29793	20A	B.1.466	GH
hCoV-19/Egypt/CUNCI-HGC12I038/2021	196	bad	EPI_ISL_890221	29793	20B	C.17	GR
hCoV-19/Egypt/CUNCI-HGC12I039/2021	4.173611	good	EPI_ISL_890222	29793	20A	B.1	GH
hCoV-19/Egypt/CUNCI-HGC12I041/2021	330.25	bad	EPI_ISL_890223	29793	20A	B.1	G
hCoV-19/Egypt/CUNCI-HGC12I042/2021	29.34028	good	EPI_ISL_890224	29793	20A	B.1.466	GH
hCoV-19/Egypt/CUNCI-HGC12I043/2021	126.5625	bad	EPI_ISL_890225	29793	20D	C.36	GR
hCoV-19/Egypt/CUNCI-HGC12I045/2021	125.4444	bad	EPI_ISL_890226	29793	20D	C.36	GR
hCoV-19/Egypt/CUNCI-HGC12I047/2021	54.17361	mediocre	EPI_ISL_890227	29793	20D	C.36	GR
hCoV-19/Egypt/CUNCI-HGC12I048/2021	10.25	good	EPI_ISL_890228	29793	20D	C.36	GR
hCoV-19/Egypt/CUNCI-HGC12I050/2021	63.67361	mediocre	EPI_ISL_890229	29793	20D	C.36	GR

hCoV-19/Egypt/CUNCI-HGC12I052/2021	15.11111	good	EPI_ISL_890230	29793	20A	B.1	GH
hCoV-19/Egypt/CUNCI-HGC12I053/2021	1296	bad	EPI_ISL_890231	29793	20B	C.17	GR
hCoV-19/Egypt/ARMY/2020	184.7198	bad	EPI_ISL_907076	29870	20D	C.36	O
hCoV-19/Egypt/ARMY-MCL001-2/2020	39.0625	mediocre	EPI_ISL_907077	29884	20A	B.1	GH
hCoV-19/Egypt/ARMY-MCL002/2020	0.694444	good	EPI_ISL_907078	29885	20D	C.36	GR
hCoV-19/Egypt/ARMY-MCL003/2020	0.694444	good	EPI_ISL_907079	29880	20D	C.36	GR
hCoV-19/Egypt/ARMY-MCL005/2020	0.694444	good	EPI_ISL_907080	29872	20D	C.36	GR
hCoV-19/Egypt/ARMY-MCL008/2020	0.694444	good	EPI_ISL_907081	29874	20D	C.36	GR
hCoV-19/Egypt/ARMY-MCL0018/2020	0.694444	good	EPI_ISL_907082	29896	20D	C.36	GR
hCoV-19/Egypt/ARMY-MCL009/2020	0.694444	good	EPI_ISL_907083	29903	20D	C.36	GR
hCoV-19/Egypt/ARMY-MCL011/2020	39.0625	mediocre	EPI_ISL_907084	29884	20A	B.1	GH
hCoV-19/Egypt/CUNCI-HGC8I033/2020	78.0625	mediocre	EPI_ISL_907086	29793	20D	C.36	GR
hCoV-19/Egypt/CUNCI-HGC9I014/2020	4.694444	good	EPI_ISL_907087	29793	20D	C.36	GR
hCoV-19/Egypt/CUNCI-HGC9I015/2020	0	good	EPI_ISL_907088	29793	20D	C.36	GR
hCoV-19/Egypt/CUNCI-HGC9I040/2020	2.777778	good	EPI_ISL_907089	29793	20D	C.36	GR
hCoV-19/Egypt/CUNCI-HGC10I010/2020	16.69444	good	EPI_ISL_907090	29793	20D	C.36	GR
hCoV-19/Egypt/CUNCI-HGC10I014/2020	17.50694	good	EPI_ISL_907092	29793	20D	C.36	GR
hCoV-19/Egypt/CUNCI-HGC11I003/2021	336.1111	bad	EPI_ISL_907093	29793	20D	C.36	GR
hCoV-19/Egypt/CUNCI-HGC09I025/2020	306.25	bad	EPI_ISL_907094	29793	20D	C.36	GR
hCoV-19/Egypt/CUNCI-HGC09I030/2020	277.7778	bad	EPI_ISL_907095	29793	20D	C.36	GR
hCoV-19/Egypt/CUNCI-HGC09I039/2020	0	good	EPI_ISL_907096	29793	19B	A.28	S
hCoV-19/Egypt/CUNCI-HGC09I041/2020	303.0625	bad	EPI_ISL_907097	29793	20A	B.1	O
hCoV-19/Egypt/CUNCI-HGC10I009/2020	169	bad	EPI_ISL_907098	29793	20A	B.1	GH
hCoV-19/Egypt/CUNCI-HGC10I021/2020	86.17361	mediocre	EPI_ISL_907099	29793	20D	C.36	GR
hCoV-19/Egypt/CUNCI-HGC10I027/2020	625.6944	bad	EPI_ISL_907100	29793	20A	B.1	G
hCoV-19/Egypt/CUNCI-HGC11I007/2021	99.17361	mediocre	EPI_ISL_907101	29793	19B	A.28	S
hCoV-19/Egypt/CUNCI-HGC11I011/2021	12.50694	good	EPI_ISL_907102	29793	20D	C.36	GR
hCoV-19/Egypt/CUNCI-HGC11I015/2021	176.5625	bad	EPI_ISL_907103	29793	20A	B.1	O
hCoV-19/Egypt/CUNCI-HGC11I021/2021	50.0625	mediocre	EPI_ISL_907104	29793	20D	C.36	GR
hCoV-19/Egypt/CUNCI-HGC11I023/2021	1601.563	bad	EPI_ISL_907105	29793	19A	A	O
hCoV-19/Egypt/CUNCI-HGC11I024/2021	529.1736	bad	EPI_ISL_907106	29793	20A	B.1	O
hCoV-19/Egypt/CUNCI-HGC11I025/2021	0	good	EPI_ISL_907107	29793	19B	A.28	S
hCoV-19/Egypt/CUNCI-HGC11I030/2021	95.0625	mediocre	EPI_ISL_907108	29793	20A	B.1.466	GH
hCoV-19/Egypt/CUNCI-HGC11I038/2021	132.1111	bad	EPI_ISL_907109	29793	20B	B.1.1	GR
hCoV-19/Egypt/CUNCI-HGC11I039/2021	116	bad	EPI_ISL_907110	29793	20D	C.36	GR
hCoV-19/Egypt/CUNCI-HGC11I046/2021	152.1111	bad	EPI_ISL_907111	29793	20D	B.1	O
hCoV-19/Egypt/CUNCI-HGC11I050/2021	0.173611	good	EPI_ISL_907112	29793	20D	C.36	GR
hCoV-19/Egypt/CUNCI-HGC11I051/2021	100	bad	EPI_ISL_907113	29793	20D	C.36	GR
hCoV-19/Egypt/CUNCI-HGC12I002/2021	121	bad	EPI_ISL_907114	29793	20A	B.1.170	G
hCoV-19/Egypt/CUNCI-HGC12I024/2021	8.506944	good	EPI_ISL_907115	29793	20D	C.36	GR
hCoV-19/Egypt/CUNCI-HGC12I025/2021	12.11111	good	EPI_ISL_907116	29793	20D	C.36	GR
hCoV-19/Egypt/CUNCI-HGC12I040/2021	251.6944	bad	EPI_ISL_907117	29793	20D	C.36	GR
hCoV-19/Egypt/CUNCI-HGC12I046/2021	188.4444	bad	EPI_ISL_907118	29793	20D	B.1	G

hCoV-19/Egypt/CPHL-NRC-5/2020	1261.565	bad	EPI_ISL_794607	29725	20A	None	G
hCoV-19/Egypt/CPHL-NRC-4/2020	2.790137	good	EPI_ISL_794608	29865	20B	B.1.1	GR
hCoV-19/Egypt/CUNCI-HGC12I049/2021	165.25	bad	EPI_ISL_1040916	29793	19B	A.28	S
hCoV-19/Egypt/NRC-01/2020	0	good	EPI_ISL_430820	29737	20A	B.1	GH
hCoV-19/Egypt/MASRI-C4-025/2020	11.11111	good	EPI_ISL_1098839	29823	20D	C.36	GR
hCoV-19/Egypt/MASRI-C4-035/2020	2.777778	good	EPI_ISL_1109484	29832	20D	C.36	GR
hCoV-19/Egypt/MASRI-C4-036/2020	2.777778	good	EPI_ISL_1109485	29862	20C	B.1.367	GH
hCoV-19/Egypt/MASRI-C4-041/2020	0	good	EPI_ISL_1109486	29823	20D	C.36	GR
hCoV-19/Egypt/MASRI-C5-001/2020	4.340278	good	EPI_ISL_1109624	29832	20D	C.36	GR
hCoV-19/Egypt/MASRI-C5-016/2020	0	good	EPI_ISL_1109625	29841	20D	C.36	GR
hCoV-19/Egypt/MASRI-C5-008/2020	0	good	EPI_ISL_1109626	29827	20D	C.36	GR
hCoV-19/Egypt/MASRI-C5-015/2020	33.50694	mediocre	EPI_ISL_1109627	29858	20A	B.1	GH
hCoV-19/Egypt/MASRI-C5-013/2020	0.173611	good	EPI_ISL_1109628	29868	20A	B.1.36.3<br>8	GH
hCoV-19/Egypt/MASRI-C5-002/2020	0	good	EPI_ISL_1109629	29834	20A	B.1	GH
hCoV-19/Egypt/MASRI-C5-019/2020	0	good	EPI_ISL_1109630	29833	20D	C.36	GR
hCoV-19/Egypt/MASRI-C4-011/2020	42.36111	mediocre	EPI_ISL_1141525	29903	20D	C.36	GR
hCoV-19/Egypt/NRC-5531/2020	0.173611	good	EPI_ISL_8189544	29903	20A	B.1.170	GH
hCoV-19/Egypt/NRC-5525/2020	0.173611	good	EPI_ISL_8189545	29874	20A	B.1.170	GH
hCoV-19/Egypt/NRC-5545/2020	2.777778	good	EPI_ISL_8189546	29903	20B	B.1.1	GR
hCoV-19/Egypt/NRC-6444/2020	117.3611	bad	EPI_ISL_8189547	29903	20B	B.1.1.1	GR
hCoV-19/Egypt/NRC-5795/2020	29.34028	good	EPI_ISL_8189548	29903	20D	B.1.1.1	GR
hCoV-19/Egypt/NRC-5794/2020	0	good	EPI_ISL_8189549	29902	20A	B.1.398	G
hCoV-19/Egypt/NRC-5451NS/2020	0.173611	good	EPI_ISL_8189550	29899	20D	C.36	GR
hCoV-19/Egypt/NRC-7475/2020	8.506944	good	EPI_ISL_8189551	29898	20D	C.36	GR
hCoV-19/Egypt/NRC-5954/2020	0.694444	good	EPI_ISL_8189552	29903	20A	B.1.170	GH
hCoV-19/Egypt/NRC-5930/2020	2.194787	good	EPI_ISL_8189553	29865	20D	C.36	GR
hCoV-19/Egypt/NRC-5552OP/2020	13750	bad	EPI_ISL_8193584	29902	20A	B.1	G
hCoV-19/Egypt/NRC-5551OP/2020	12397.27	bad	EPI_ISL_8193585	29873	20A	B.1.36.3<br>9	G
hCoV-19/Egypt/NRC-5550OP/2020	518.9329	bad	EPI_ISL_8193586	29876	20D	B.1.1.1	G
hCoV-19/Egypt/NRC-7090/2020	23077.26	bad	EPI_ISL_8193587	29900	20A	B.1.517	G
hCoV-19/Egypt/NRC-7076/2020	25147.19	bad	EPI_ISL_8193588	29877	20A	B.1.36.3<br>9	GH
hCoV-19/Egypt/NRC-7071/2020	5178.318	bad	EPI_ISL_8193589	29878	20D	B.1.1	GR
hCoV-19/Egypt/NRC-7068/2020	7284.057	bad	EPI_ISL_8193590	29893	20A	B.1.111	GH
hCoV-19/Egypt/NRC-6999/2020	77.57742	mediocre	EPI_ISL_8193591	29872	20A	B.1	GH
hCoV-19/Egypt/NRC-6994/2020	264.3088	bad	EPI_ISL_8193592	29884	20A	B.1	GH
hCoV-19/Egypt/NRC-6662/2020	43761.17	bad	EPI_ISL_8193593	29884	20B	B.1.1.51	GR
hCoV-19/Egypt/NRC-6661/2020	23429.34	bad	EPI_ISL_8193595	29883	20A	B.1	G
hCoV-19/Egypt/NRC-6660/2020	26579.9	bad	EPI_ISL_8193596	29899	20A	B.1	G
hCoV-19/Egypt/NRC-6659/2020			EPI_ISL_8193597	29892	None	B.1.1.19<br>2	O
hCoV-19/Egypt/NRC-6658/2020	12856.25	bad	EPI_ISL_8193598	29899	20D	B.1.1.19<br>2	GR
hCoV-19/Egypt/NRC-6657/2020	33457.02	bad	EPI_ISL_8193599	29886	20B	B.1.362	O
hCoV-19/Egypt/NRC-6645/2020	31295.31	bad	EPI_ISL_8193600	29901	20A	B.1.36.3<br>9	O

hCoV-19/Egypt/NRC-6641/2020	40943.92	bad	EPI_ISL_8193601	29894	20A	B.1	G
hCoV-19/Egypt/NRC-6040/2020	40188.18	bad	EPI_ISL_8193602	29869	20D	B.1	G
hCoV-19/Egypt/NRC-6129/2020	56.25	mediocre	EPI_ISL_8193603	29897	20B	B.1.1	GR
hCoV-19/Egypt/NRC-6109/2020	20551.56	bad	EPI_ISL_8193604	29893	20A	B.1.195	G
hCoV-19/Egypt/NRC-6108/2020	10617.36	bad	EPI_ISL_8193605	29897	20A	B.1	O
hCoV-19/Egypt/NRC-6081/2020	11250.69	bad	EPI_ISL_8193606	29908	20A	B.1	G
hCoV-19/Egypt/NRC-6078/2020	67745.29	bad	EPI_ISL_8193607	29874	20B	B.1.1.51	GR
hCoV-19/Egypt/NRC-6074/2020	9514.063	bad	EPI_ISL_8193608	29901	20A	B.1	G
hCoV-19/Egypt/NRC-6072/2020	86523.82	bad	EPI_ISL_8193609	29898	20A	B.1	G
hCoV-19/Egypt/NRC-6063/2020	14762.19	bad	EPI_ISL_8193610	29846	20D	B.1.1.11<br>7	GR
hCoV-19/Egypt/NRC-6057/2020	7606.25	bad	EPI_ISL_8193611	29881	20D	B.1.1.1	G
hCoV-19/Egypt/NRC-6056/2020	305.1235	bad	EPI_ISL_8193612	29865	20D	B.1	G
hCoV-19/Egypt/NRC-6049/2020	7546.007	bad	EPI_ISL_8193613	29904	20A	B.1	GH
hCoV-19/Egypt/NRC-6047/2020	20362.5	bad	EPI_ISL_8193614	29894	20A	B.1	O
hCoV-19/Egypt/NRC-6043/2020	22300.17	bad	EPI_ISL_8193615	29876	20A	B.1.36.3<br>9	G
hCoV-19/Egypt/NRC-6036/2020	29144.33	bad	EPI_ISL_8193616	29776	20A	B.1	G
hCoV-19/Egypt/NRC-6912/2020	7027.018	bad	EPI_ISL_8193617	29872	20D	B.1.1.1	G
hCoV-19/Egypt/NRC-6902/2020	37131.91	bad	EPI_ISL_8193618	29877	20B	B.1.1.51	G
hCoV-19/Egypt/NRC-6398/2020	274.2378	bad	EPI_ISL_8193619	29901	20A	B.1	GH
hCoV-19/Egypt/NRC-6379/2020	34656.42	bad	EPI_ISL_8193620	29893	20A	B.1	G
hCoV-19/Egypt/NRC-6390/2020	32084.96	bad	EPI_ISL_8193621	29876	20A	B.1.36.3<br>9	GH
hCoV-19/Egypt/NRC-6386/2020	28229.12	bad	EPI_ISL_8193622	29879	20A	B.1	GH
hCoV-19/Egypt/NRC-5538OP/2020	13523.13	bad	EPI_ISL_8193623	29881	20A	B.1.456	GH
hCoV-19/Egypt/NRC-5521NS/2020	27537.63	bad	EPI_ISL_8193624	29890	20A	B.1.36.3<br>9	G
hCoV-19/Egypt/NRC-5486/2020	7366.129	bad	EPI_ISL_8193625	29877	20D	B.1.1.19<br>2	G
hCoV-19/Egypt/NRC-5466NS/2020	287.6736	bad	EPI_ISL_8193626	29872	20B	B.1.1	GR
hCoV-19/Egypt/NRC-5459NS/2020	3107.813	bad	EPI_ISL_8193627	29880	20A	B.1	O
hCoV-19/Egypt/NRC-6735/2020	72618.51	bad	EPI_ISL_8193628	29896	20D	B.1.517	G
hCoV-19/Egypt/NRC-6725/2020	26487.5	bad	EPI_ISL_8193629	29908	20A	B.1	O
hCoV-19/Egypt/NRC-5438NS/2020	14518.75	bad	EPI_ISL_8193630	29880	20A	B.1.371	O
hCoV-19/Egypt/NRC-5435NS/2020	5408.47	bad	EPI_ISL_8193631	29873	20A	B.1.533	G
hCoV-19/Egypt/NRC-5441NS/2020	7484.028	bad	EPI_ISL_8193632	29890	20D	B.1.187	G
hCoV-19/Egypt/NRC-7340/2020	5856.424	bad	EPI_ISL_8193633	29899	20A	B.1.170	GH
hCoV-19/Egypt/NRC-7316/2020	629.6968	bad	EPI_ISL_8193634	29870	20D	B.1	G
hCoV-19/Egypt/NRC-7314/2020	4555.179	bad	EPI_ISL_8193635	29871	20A	B.1	GH
hCoV-19/Egypt/NRC-7313/2020	76479.2	bad	EPI_ISL_8193636	29880	20A	B.1.517	G
hCoV-19/Egypt/NRC-7307/2020	40360.09	bad	EPI_ISL_8193637	29878	20D	B.1	GH
hCoV-19/Egypt/NRC-7306/2020	37618.63	bad	EPI_ISL_8193638	29886	20D	B.1	G
hCoV-19/Egypt/NRC-7305/2020	30941.21	bad	EPI_ISL_8193639	29875	20A	B.1	G
hCoV-19/Egypt/NRC-7297/2020	43949.03	bad	EPI_ISL_8193640	29876	20A	B.1	G
hCoV-19/Egypt/NRC-7292/2020	15681.25	bad	EPI_ISL_8193641	29890	20A	B.1.293	G
hCoV-19/Egypt/NRC-7283/2020	2912.674	bad	EPI_ISL_8193642	29899	20D	B.1.187	O

hCoV-19/Egypt/NRC-7282/2020	26372.3	bad	EPI_ISL_8193643	29871	20A	B.1.517	G
hCoV-19/Egypt/NRC-7269/2020	13520.45	bad	EPI_ISL_8193644	29539	20A	B.1	G
hCoV-19/Egypt/NRC-6612/2020	24491.36	bad	EPI_ISL_8193645	29861	20A	B.1	G
hCoV-19/Egypt/NRC-6602/2020	24204.17	bad	EPI_ISL_8193646	29876	20D	B.1.36.3<br>9	G
hCoV-19/Egypt/NRC-6590/2020	16194.41	bad	EPI_ISL_8193647	29898	20D	B.1.362	GR
hCoV-19/Egypt/NRC-6519/2020	14266.62	bad	EPI_ISL_8193648	29562	20A	B.1.36.3<br>9	G
hCoV-19/Egypt/NRC-6509/2020	824.4616	bad	EPI_ISL_8193649	29569	20A	B.1	G
hCoV-19/Egypt/NRC-6506/2020	1859.736	bad	EPI_ISL_8193650	29542	20A	B.1.517	G
hCoV-19/Egypt/NRC-6505/2020	4737.5	bad	EPI_ISL_8193651	29899	20D	B.1	G
hCoV-19/Egypt/NRC-5555OP/2020	1006.424	bad	EPI_ISL_8193653	29904	20D	C.36	GR
hCoV-19/Egypt/NRC-5555NS/2020	64.75694	mediocre	EPI_ISL_8193654	29902	20D	C.36	GR
hCoV-19/Egypt/NRC-5556NS/2020	56.25	mediocre	EPI_ISL_8193655	29903	20D	C.36	GR
hCoV-19/Egypt/NRC-5558NS/2020	1192.381	bad	EPI_ISL_8193656	29902	20D	C.36	GR
hCoV-19/Egypt/NRC-5558OP/2020	56.25	mediocre	EPI_ISL_8193657	29904	20D	C.36	GR
hCoV-19/Egypt/NRC-5581OP/2020	665.2778	bad	EPI_ISL_8193658	29904	20B	B.1.1	GR
hCoV-19/Egypt/NRC-5575OP/2020	1727.257	bad	EPI_ISL_8193659	29873	20D	C.36.3.1	GR
hCoV-19/Egypt/NRC-5578OP/2020	926.5625	bad	EPI_ISL_8193660	29898	20B	B.1.1.29<br>4	G
hCoV-19/Egypt/NRC-6335/2020	1048.383	bad	EPI_ISL_8193851	29870	20A	B.1.170	GH
hCoV-19/Egypt/NRC-6336/2020	2630.018	bad	EPI_ISL_8193852	29882	20D	B.1.1.29<br>4	GR
hCoV-19/Egypt/NRC-6337/2020	281.5968	bad	EPI_ISL_8193853	29878	20A	B.1.170	GH
hCoV-19/Egypt/NRC-6445/2020	1171.057	bad	EPI_ISL_8193854	29886	20A	B.1.170	GH
hCoV-19/Egypt/NRC-6446/2020	684.0278	bad	EPI_ISL_8193855	29896	20A	B.1.170	GH
hCoV-19/Egypt/NRC-6334/2020	857.8125	bad	EPI_ISL_8193856	29900	20A	B.1.170	GH
hCoV-19/Egypt/NRC-6333/2020	325.0231	bad	EPI_ISL_8193857	29893	20A	B.1.170	GH
hCoV-19/Egypt/NRC-6332/2020	1102.778	bad	EPI_ISL_8193858	29880	20A	B.1.170	GH
hCoV-19/Egypt/NRC-6209/2020	389.7569	bad	EPI_ISL_8193859	29583	20B	B.1.1.1	GR
hCoV-19/Egypt/NRC-6207/2020	606.25	bad	EPI_ISL_8193860	29870	20B	B.1.1.1	GR
hCoV-19/Egypt/NRC-6206/2020	729.3403	bad	EPI_ISL_8193861	29643	20B	B.1.1.1	GR
hCoV-19/Egypt/NRC-6205/2020	562.5242	bad	EPI_ISL_8193862	29886	20A	B.1.170	GH
hCoV-19/Egypt/NRC-6201/2020	270.7373	bad	EPI_ISL_8193863	29855	20A	B.1	GH
hCoV-19/Egypt/NRC-6199/2020	106.4236	bad	EPI_ISL_8193864	29901	20A	B.1.36.3<br>1	GH
hCoV-19/Egypt/NRC-5792/2020	331.4236	bad	EPI_ISL_8193866	29886	20A	B.1	GH
hCoV-19/Egypt/NRC-5771/2020	259.0278	bad	EPI_ISL_8193867	29896	20A	B.1	G
hCoV-19/Egypt/NRC-5755/2020	3708.516	bad	EPI_ISL_8193868	29886	20A	B.1	GH
hCoV-19/Egypt/NRC-5754/2020	301.5625	bad	EPI_ISL_8193869	29886	20A	B.1.170	GH
hCoV-19/Egypt/NRC-5726/2020	462.5	bad	EPI_ISL_8193870	29882	20D	B.1.1.1	O
hCoV-19/Egypt/NRC-5455NS/2020	642.376	bad	EPI_ISL_8193871	29822	20A	B.1.170	G
hCoV-19/Egypt/NRC-5448NS/2020	106.4236	bad	EPI_ISL_8193872	29872	20B	B.1.1.1	G
hCoV-19/Egypt/NRC-5445NS/2020	226.5625	bad	EPI_ISL_8193873	29901	20D	C.36	GR
hCoV-19/Egypt/NRC-5443NS/2020	204.3403	bad	EPI_ISL_8193874	29870	20A	B.1.170	G
hCoV-19/Egypt/NRC-5450OP/2020	3984.627	bad	EPI_ISL_8193875	29872	20A	B.1	G
hCoV-19/Egypt/NRC-5448OP/2020	56.49276	mediocre	EPI_ISL_8193876	29871	20D	C.36	GR

hCoV-19/Egypt/NRC-5445OP/2020	1252.461	bad	EPI_ISL_8193877	29882	20D	C.36	GR
hCoV-19/Egypt/NRC-5443OP/2020	1923.55	bad	EPI_ISL_8193878	29899	20B	B.1.1	GR
hCoV-19/Egypt/NRC-6290/2020	944.6354	bad	EPI_ISL_8193879	29882	20B	B.1.1	GR
hCoV-19/Egypt/NRC-6285/2020	642.6616	bad	EPI_ISL_8193880	29873	20B	B.1.1	GR
hCoV-19/Egypt/NRC-6172/2020	2475.191	bad	EPI_ISL_8193881	29886	20B	B.1.1.1	GR
hCoV-19/Egypt/NRC-6283/2020	2939.063	bad	EPI_ISL_8193882	29875	20D	B.1.533	G
hCoV-19/Egypt/NRC-6237/2020	528.0367	bad	EPI_ISL_8193883	29856	20A	B.1.170	GH
hCoV-19/Egypt/NRC-6187/2020	535.8115	bad	EPI_ISL_8193884	29852	20A	B.1	GH
hCoV-19/Egypt/NRC-6167/2020	606.25	bad	EPI_ISL_8193885	29872	20A	B.1.170	GH
hCoV-19/Egypt/NRC-6231/2020	246.0105	bad	EPI_ISL_8193886	29865	20A	B.1.170	GH
hCoV-19/Egypt/NRC-6301/2020	73.61111	mediocre	EPI_ISL_8193887	29900	20A	B.1.170	GH
hCoV-19/Egypt/NRC-6292/2020	301.7803	bad	EPI_ISL_8193888	29873	20A	B.1.170	GH
hCoV-19/Egypt/NRC-6178/2020	246.2111	bad	EPI_ISL_8193889	29887	20A	B.1.170	GH
hCoV-19/Egypt/NRC-6174/2020	489.0625	bad	EPI_ISL_8193890	29870	20A	B.1.170	GH
hCoV-19/Egypt/NRC-6173/2020	3166.605	bad	EPI_ISL_8193891	29905	20A	B.1.1.37<br>2	GH
hCoV-19/Egypt/NRC-5772/2020	62.5	mediocre	EPI_ISL_8193892	29860	20A	B.1.170	GH
hCoV-19/Egypt/NRC-6171/2020	2384.273	bad	EPI_ISL_8193893	29883	20B	B.1	G
hCoV-19/Egypt/NRC-6169/2020	1040.866	bad	EPI_ISL_8193894	29876	20B	B.1	G
hCoV-19/Egypt/NRC-6135/2020	2738.811	bad	EPI_ISL_8193895	29885	20A	None	G
hCoV-19/Egypt/NRC-6164/2020	6408.049	bad	EPI_ISL_8193896	29882	20A	B.1.1	G
hCoV-19/Egypt/NRC-6163/2020	1934.949	bad	EPI_ISL_8193897	29879	20A	B.1	G
hCoV-19/Egypt/NRC-6553/2020	1562.5	bad	EPI_ISL_8193898	29875	20A	B.1.170	GH
hCoV-19/Egypt/NRC-6452/2020	7242.454	bad	EPI_ISL_8193899	29884	20A	B.1	O
hCoV-19/Egypt/NRC-6451/2020	8641.751	bad	EPI_ISL_8193900	29902	20A	B.1	O
hCoV-19/Egypt/NRC-6450/2020	3575.96	bad	EPI_ISL_8193901	29874	20A	B.1.417	G
hCoV-19/Egypt/NRC-6541/2020	1056.25	bad	EPI_ISL_8193902	29876	20A	B.1	O
hCoV-19/Egypt/NRC-6538/2020	3096.182	bad	EPI_ISL_8193903	29880	20D	B.1	GH
hCoV-19/Egypt/NRC-6033/2020	5269.138	bad	EPI_ISL_8193904	29889	20B	B.1.1	GR
hCoV-19/Egypt/NRC-6032/2020	2707.149	bad	EPI_ISL_8193905	29896	20B	B.1.1.1	O
hCoV-19/Egypt/NRC-6023/2020	11108.64	bad	EPI_ISL_8193906	29884	20A	B.1	G
hCoV-19/Egypt/NRC-6031/2020	4348.177	bad	EPI_ISL_8193907	29906	20A	B.1	GH
hCoV-19/Egypt/NRC-6017/2020	11958.12	bad	EPI_ISL_8193908	29889	20A	None	GH
hCoV-19/Egypt/NRC-6006/2020	5939.76	bad	EPI_ISL_8193909	29881	20A	B.1.575	G
hCoV-19/Egypt/NRC-6944/2020	309.0278	bad	EPI_ISL_8193910	29898	20B	B.1.1.31<br>2	GR
hCoV-19/Egypt/NRC-5965/2020	3096.07	bad	EPI_ISL_8193911	29874	20A	B.1.417	GH
hCoV-19/Egypt/NRC-5964/2020	2320.077	bad	EPI_ISL_8193912	29878	20A	B.1	GH
hCoV-19/Egypt/NRC-5962/2020	259.0278	bad	EPI_ISL_8193913	29776	20A	B.1	GH
hCoV-19/Egypt/NRC-5961/2020	498.7003	bad	EPI_ISL_8193914	29904	20B	B.1.1	GR
hCoV-19/Egypt/NRC-5955/2020	2815.661	bad	EPI_ISL_8193915	29877	20B	B.1.533	G
hCoV-19/Egypt/NRC-5953/2020	724.3335	bad	EPI_ISL_8193916	29873	20A	B.1.170	GH
hCoV-19/Egypt/NRC-5952/2020	58.29218	mediocre	EPI_ISL_8193917	29866	20A	B.1.170	GH
hCoV-19/Egypt/NRC-5950/2020	6220.213	bad	EPI_ISL_8193918	29879	20B	B.1.1	GR
hCoV-19/Egypt/NRC-5949/2020	243.4597	bad	EPI_ISL_8193919	29865	20A	B.1.170	GH

hCoV-19/Egypt/NRC-5944/2020	2671.664	bad	EPI_ISL_8193920	29888	20B	B.1.1.1	GR
hCoV-19/Egypt/NRC-5943/2020	515.0534	bad	EPI_ISL_8193921	29877	20A	B.1	GH
hCoV-19/Egypt/NRC-5940/2020	1221.889	bad	EPI_ISL_8193922	29873	20D	C.36.3.1	GR
hCoV-19/Egypt/NRC-5939/2020	576.4392	bad	EPI_ISL_8193923	29875	20B	B.1	G
hCoV-19/Egypt/NRC-5938/2020	254.3482	bad	EPI_ISL_8193924	29886	20A	B.1	GH
hCoV-19/Egypt/NRC-5936/2020	85.59028	mediocre	EPI_ISL_8193925	29886	20A	B.1	GH
hCoV-19/Egypt/NRC-6350/2020	1618.924	bad	EPI_ISL_8194864	29905	20A	B.1	GH
hCoV-19/Egypt/NRC-6064/2020	1066.84	bad	EPI_ISL_8194865	29900	20D	B.1.1.1	GR
hCoV-19/Egypt/NRC-6559/2020	302.5625	bad	EPI_ISL_8194866	29895	20D	C.36	GR
hCoV-19/Egypt/NRC-6469/2020	7970.313	bad	EPI_ISL_8194867	29874	20D	C.36	GR
hCoV-19/Egypt/NRC-6453/2020	12262.1	bad	EPI_ISL_8194868	29896	20B	B.1.1.1	G
hCoV-19/Egypt/NRC-6359/2020	4243.75	bad	EPI_ISL_8194869	29878	20D	B.1.533	G
hCoV-19/Egypt/NRC-6701/2020	10212.74	bad	EPI_ISL_8194870	29905	20A	B.1.221	G
hCoV-19/Egypt/NRC-6367/2020	488.0693	bad	EPI_ISL_8194871	29885	20D	C.36	GR
hCoV-19/Egypt/NRC-5616/2020	2417.53	bad	EPI_ISL_8194872	29871	20D	B.1.1.1	GR
hCoV-19/Egypt/NRC-5616OP/2020	225.6944	bad	EPI_ISL_8194873	29873	20D	C.36	GR
hCoV-19/Egypt/NRC-5621/2020	11027.43	bad	EPI_ISL_8194874	29901	20A	B.1	G
hCoV-19/Egypt/NRC-5627/2020	1587.809	bad	EPI_ISL_8194875	29901	20A	B.1.284	GH
hCoV-19/Egypt/NRC-5635/2020	5407.813	bad	EPI_ISL_8194876	29886	20B	B.1.1	GR
hCoV-19/Egypt/NRC-5632/2020	259.0278	bad	EPI_ISL_8194877	29879	20A	B.1.170	GH
hCoV-19/Egypt/NRC-5645/2020	246.0069	bad	EPI_ISL_8194878	29873	20A	B.1.170	GH
hCoV-19/Egypt/NRC-5642OP/2020	281.25	bad	EPI_ISL_8194879	29887	20B	B.1.533	G
hCoV-19/Egypt/NRC-6365/2020	11358.4	bad	EPI_ISL_8194880	29881	20A	B.1.195	GH
hCoV-19/Egypt/NRC-6351/2020	35563.8	bad	EPI_ISL_8194881	29901	20A	B.1	G
hCoV-19/Egypt/NRC-6352/2020	29791.46	bad	EPI_ISL_8194882	29882	20A	B.1	GH
hCoV-19/Egypt/NRC-6353/2020	12415.97	bad	EPI_ISL_8194883	29897	20A	B.1	GH
hCoV-19/Egypt/NRC-6357/2020	13033.5	bad	EPI_ISL_8194884	29906	20A	B.1.180	G
hCoV-19/Egypt/NRC-6376/2020	1236.111	bad	EPI_ISL_8194885	29898	20D	B.1.1.1	GR
hCoV-19/Egypt/NRC-6575/2020	11208.22	bad	EPI_ISL_8194886	29872	20A	B.1	GH
hCoV-19/Egypt/NRC-6382/2020	1206.252	bad	EPI_ISL_8194887	29878	20A	B.1	O
hCoV-19/Egypt/NRC-6318/2020	5254.861	bad	EPI_ISL_8194888	29877	20D	B.1	GH
hCoV-19/Egypt/NRC-6320/2020	3644.352	bad	EPI_ISL_8194889	29870	20A	B.1	G
hCoV-19/Egypt/NRC-6322/2020			EPI_ISL_8194890	29870	None	B.1.1.372	GR
hCoV-19/Egypt/NRC-6381/2020	975.6944	bad	EPI_ISL_8194891	29894	20D	B.1	G
hCoV-19/Egypt/NRC-6380/2020	1757.813	bad	EPI_ISL_8194892	29885	20B	B.1.1.1	GR
hCoV-19/Egypt/NRC-7615/2021	44.44444	mediocre	EPI_ISL_8215716	29871	20A	B.1.36.31	GH
hCoV-19/Egypt/NRC-581/2021	8.506944	good	EPI_ISL_8215717	29896	20D	C.36.3	GR
hCoV-19/Egypt/NRC-627/2021	17.36111	good	EPI_ISL_8215718	29893	20D	C.36.3	GR
hCoV-19/Egypt/NRC-628/2021	487.6736	bad	EPI_ISL_8215719	29864	20D	C.36	GR
hCoV-19/Egypt/NRC-637/2021	0	good	EPI_ISL_8215720	29895	20D	C.36.3	GR
hCoV-19/Egypt/NRC-657/2021	494.4444	bad	EPI_ISL_8215721	29883	20I (Alpha, V1)	B.1.1.7	GRY
hCoV-19/Egypt/NRC-659/2021	2567.121	bad	EPI_ISL_8215722	29887	20D	B.1.1	GR

hCoV-19/Egypt/NRC-681/2021	1658.507	bad	EPI_ISL_8215723	29894	20D	C.36.3	GR
hCoV-19/Egypt/NRC-690/2021	459.0278	bad	EPI_ISL_8215724	29896	20D	B.1.516	G
hCoV-19/Egypt/NRC-533/2021	1132.084	bad	EPI_ISL_8251483	29892	20D	C.36	GR
hCoV-19/Egypt/NRC-536/2021	516.8403	bad	EPI_ISL_8251484	29896	20D	C.36.3	GR
hCoV-19/Egypt/NRC-537/2021	223.0903	bad	EPI_ISL_8251485	29865	20D	C.38	GR
hCoV-19/Egypt/NRC-589/2021	1017.501	bad	EPI_ISL_8251486	29861	20D	C.36.3	GR
hCoV-19/Egypt/NRC-596/2021	4951.867	bad	EPI_ISL_8251487	29895	20D	B.1	G
hCoV-19/Egypt/NRC-603/2021	389.7569	bad	EPI_ISL_8251488	29886	20D	C.36.3	GR
hCoV-19/Egypt/NRC-608/2021	745.3125	bad	EPI_ISL_8251489	29899	20D	C.36	GR
hCoV-19/Egypt/NRC-613/2021	350.6944	bad	EPI_ISL_8251490	29864	20A	B.1.170	GH
hCoV-19/Egypt/NRC-617/2021	3304.34	bad	EPI_ISL_8251491	29904	20A	B.1	G
hCoV-19/Egypt/NRC-623/2021	1500.694	bad	EPI_ISL_8251492	29895	20D	C.36.3	GR
hCoV-19/Egypt/NRC-635/2021	316.8403	bad	EPI_ISL_8251493	29884	20D	C.36.3	GR
hCoV-19/Egypt/NRC-639/2021	64.75694	mediocre	EPI_ISL_8251494	29880	20D	C.36.3	GR
hCoV-19/Egypt/NRC-650/2021	1850	bad	EPI_ISL_8251495	29876	20D	C.17	GR
hCoV-19/Egypt/NRC-651/2021	2140.816	bad	EPI_ISL_8251496	29860	20D	B.1	G
hCoV-19/Egypt/NRC-652/2021	1782.813	bad	EPI_ISL_8251497	29897	20I (Alpha, V1)	None	GR
hCoV-19/Egypt/NRC-660/2021	961.1111	bad	EPI_ISL_8251498	29882	20D	B.1.516	G
hCoV-19/Egypt/NRC-662/2021	4381.485	bad	EPI_ISL_8251499	29897	20D	B.1.187	G
hCoV-19/Egypt/NRC-678/2021	1782.82	bad	EPI_ISL_8251500	29865	20D	C.36.3	GR
hCoV-19/Egypt/NRC-682/2021	1381.25	bad	EPI_ISL_8251501	29881	20A	B.1	G
hCoV-19/Egypt/NRC-694/2021	3885.593	bad	EPI_ISL_8251502	29875	20D	B.1.187	G
hCoV-19/Egypt/NRC-697/2021	4881.25	bad	EPI_ISL_8251503	29892	20D	B.1	G
hCoV-19/Egypt/NRC-699/2021	1264.757	bad	EPI_ISL_8251504	29896	20D	B.1	G
hCoV-19/Egypt/NRC-704/2021	141.8403	bad	EPI_ISL_8251505	29891	20D	C.36.3	GR
hCoV-19/Egypt/NRC-731/2021	1006.944	bad	EPI_ISL_8251507	29893	20D	B.1.1.1	GR
hCoV-19/Egypt/NRC-7088/2021	22833.29	bad	EPI_ISL_8251513	29873	20A	B.1.36.39	GH
hCoV-19/Egypt/NRC-7082/2021	42976.86	bad	EPI_ISL_8251514	29901	20A	B.1.36.39	O
hCoV-19/Egypt/USC-1/2021	0	good	EPI_ISL_8464608	584	19A	None	O
hCoV-19/Egypt/USC-2/2021	0	good	EPI_ISL_8466472	579	21H (Mu)	None	O
hCoV-19/Egypt/USC-3/2021	0	good	EPI_ISL_8469597	656	19A	None	O
hCoV-19/Egypt/USC-4/2021	0	good	EPI_ISL_8469870	627	19A	None	O
hCoV-19/Egypt/USC-5/2021	0	good	EPI_ISL_8470239	585	19A	None	O
hCoV-19/Egypt/USC-6/2021	0	good	EPI_ISL_8470721	626	20C	None	O
hCoV-19/Egypt/CPHL-EGY21143/2021	672.179	bad	EPI_ISL_9047493	29855	21J (Delta)	AY.100	O
hCoV-19/Egypt/CPHL-EGY21144/2020	424.6801	bad	EPI_ISL_9047494	29907	21J (Delta)	AY.122	O
hCoV-19/Egypt/CPHL-EGY21215/2021	751.3541	bad	EPI_ISL_9047564	29643	21J (Delta)	AY.125	G
hCoV-19/Egypt/CPHL-EGY21216/2021	1772.859	bad	EPI_ISL_9047565	29641	21J (Delta)	None	G
hCoV-19/Egypt/CPHL-EGY21217/2021	1112.901	bad	EPI_ISL_9047566	29637	21J (Delta)	None	G
hCoV-19/Egypt/CPHL-EGY21218/2021	762.5049	bad	EPI_ISL_9047567	29636	21J (Delta)	AY.125	G

hCoV-19/Egypt/CPHL-EGY21219/2021	1676.286	bad	EPI_ISL_9047568	29641	21J (Delta)	None	G
hCoV-19/Egypt/CPHL-EGY21220/2021	748.5732	bad	EPI_ISL_9047569	29635	21J (Delta)	AY.122	G
hCoV-19/Egypt/CPHL-EGY21221/2021	2058.777	bad	EPI_ISL_9047570	29632	21J (Delta)	None	G
