# Supplementary material for: Insight into Genetic Characteristics of Identified SARS-CoV-2 Variants in Egypt from March 2020 to May 2021
Source: Pathogens. 2022 Jul 26;11(8):834. doi: 10.3390/pathogens11080834 (PMC9330621; doi:10.3390/pathogens11080834)
Supplement: Supplementary file 1 [file pathogens-11-00834-s001.zip › Supplement Table S2.pdf]

Supplement Table S2: shows the most common amino acid mutations among different lineages occurring in Egypt between March 2020 and May 2021.

Clade	%	N gene	ORF1a	ORF1b	ORF3a	ORF8	M gene	ORF7b
A.28	(1.7%)	A35V, S202N	V86F, A3529T	D1183Y, A975V	S171L			
B.1	(4%)			P314L		S84L		
B.1.1	(5.4%)	R203K(50.5%), G204R(38.3%)		P314L		S84L		
B.1.1.1	(1.4%)	R203K(50.5%), G204R(38.3%)	T1246I, G3278S	P314L		S84L		
B.1.1.7	(0.7%)	D3L, R203K(50.5%), G204R(38.3%), S235F	T1001I, A1708D, I2230T, del3675/3677	P314L		S84L,Y73C, Q27*, R52I		
B.1.170	6.3%)			P314L	Q57H(30%), S235F	S84L		
B.1.36.38	0.02	S194L(2%)	V561A	P314L	Q57H(30%)	S84L		
C.36	(27.5%)	R203K(50.5%), G204R(38.3%)	T4090I, T1246I, G3278S	P314L		S84L		
C.36.3	(9%)	R203K(50.5%), G204R(38.3%), G212V(52%)	D3222N,L3691S,T1246I D2980N, S3687L, E102K, G3278S, P2287S, T4090I, D1639N, A859V	P314L, D1028Y		S84L	I82T	A43S
C.38(	1%)	R203K(50.5%), G204R(38.3%), P13L, E378Q, A211V	G2207C G3278S, K1348Q, H1500Y, del138/144,T1246I , R2115I, T3646A	P314L, A2132V		S84L, T11K	S84L	
