# Supplementary figures and images for: Insight into Genetic Characteristics of Identified SARS-CoV-2 Variants in Egypt from March 2020 to May 2021
Source: Pathogens. 2022 Jul 26;11(8):834. doi: 10.3390/pathogens11080834 (PMC9330621; doi:10.3390/pathogens11080834)

Figure S1: Maximum likelihood (ML) phylogeny of Egyptian SARS-CoV-2 whole-genome sequences.

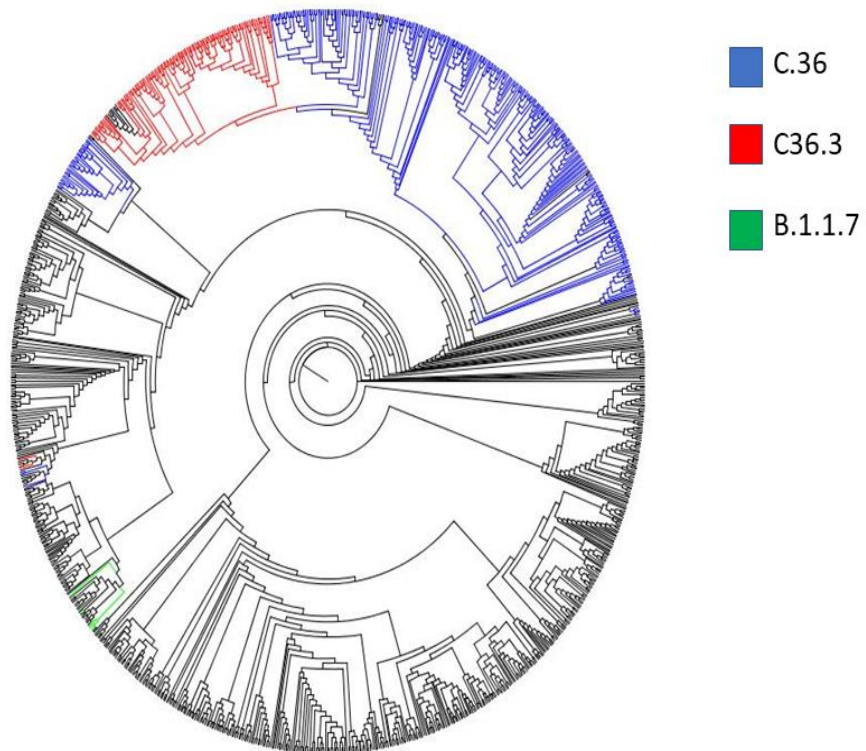

Supplement: Supplementary file 1 [file pathogens-11-00834-s001.zip › Supplement Figure S1.pdf]

Figure S2: cumulative of C.36.3 prevalence worldwide

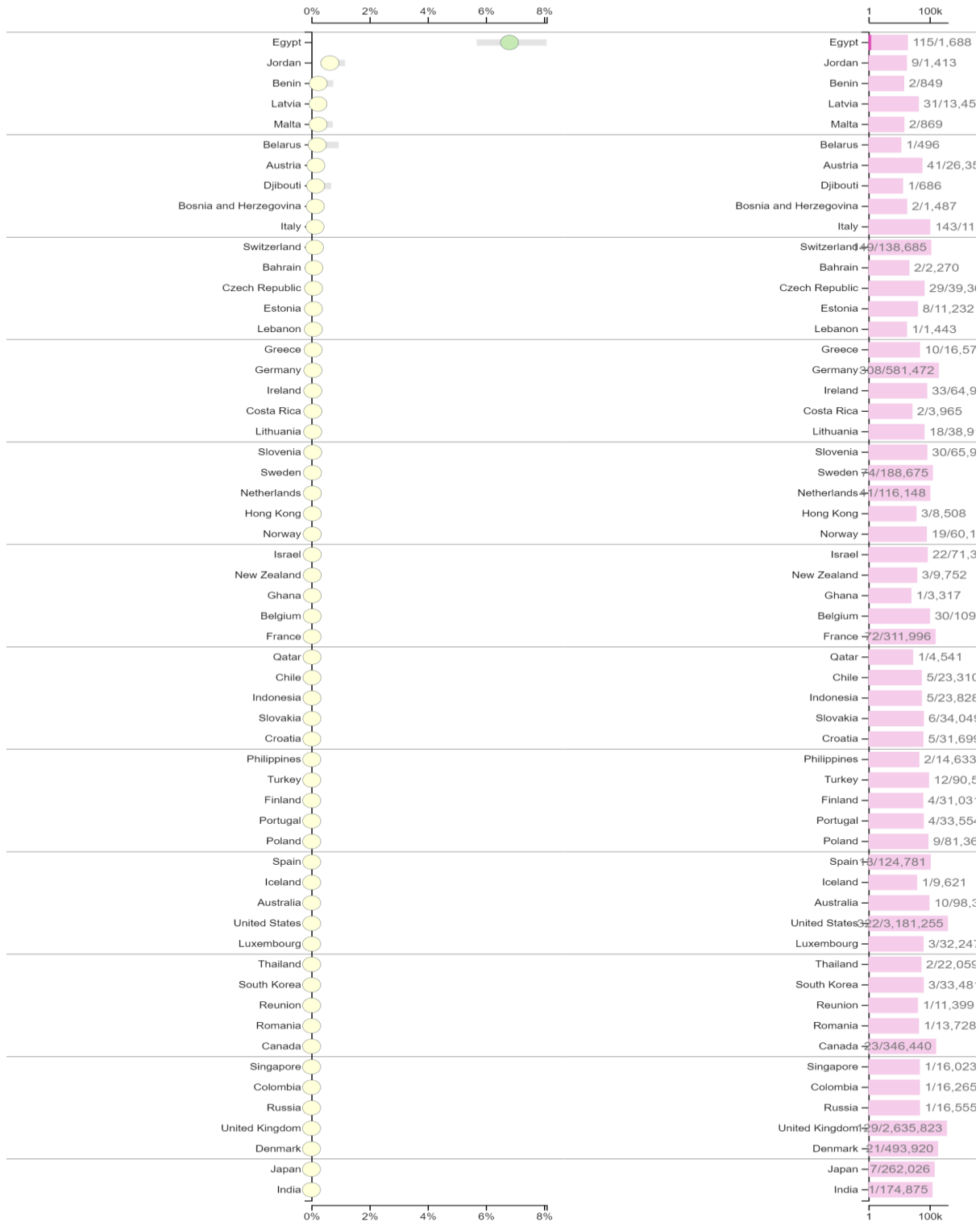

Supplement: Supplementary file 1 [file pathogens-11-00834-s001.zip › Supplement Figure S2.pdf]

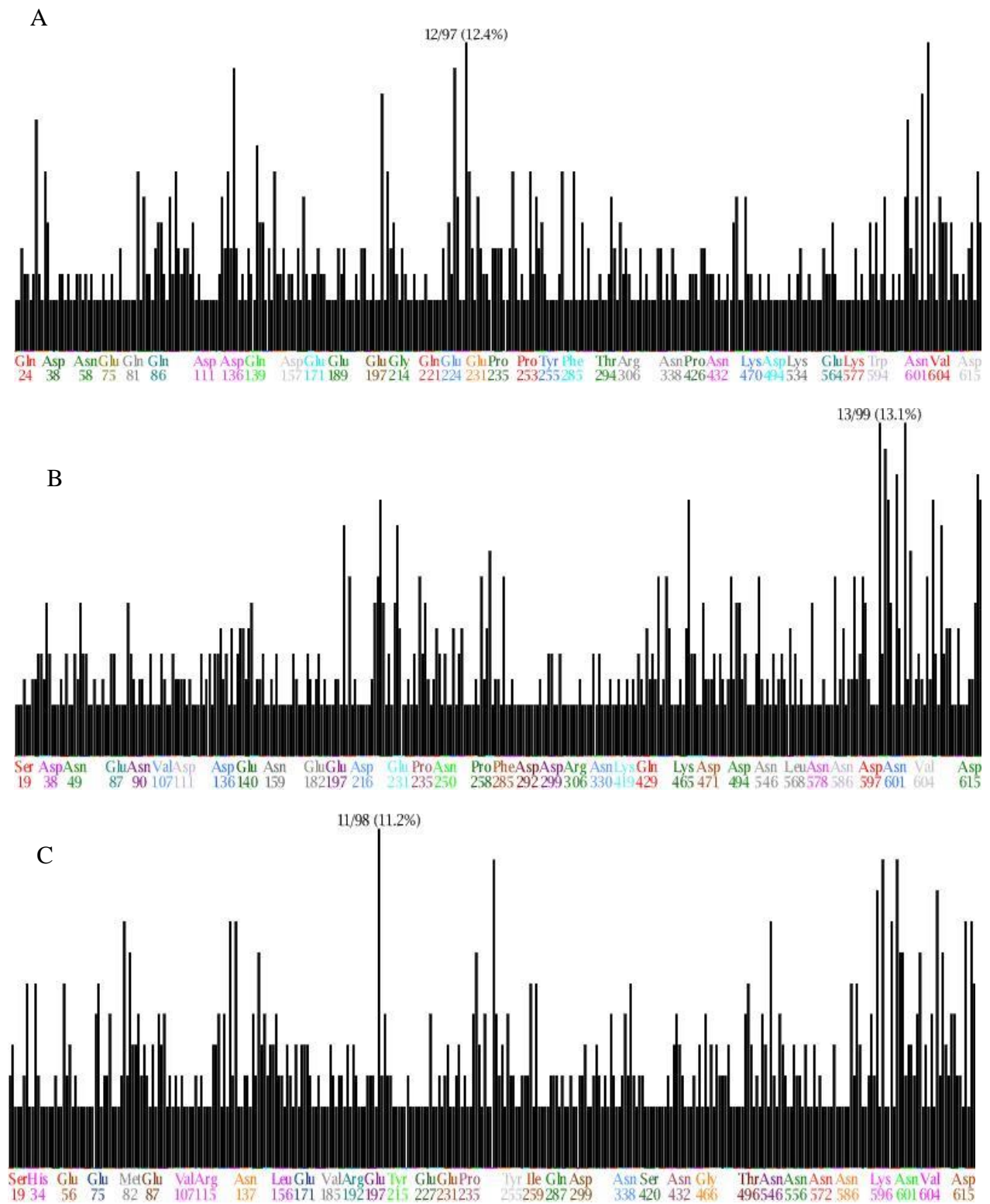

**Supplement Figure S3.** PLIF for the Egyptian strains-*hACE2* docking (in population form).

Supplement: Supplementary file 1 [file pathogens-11-00834-s001.zip › Supplement Figure S3.pdf]
